# A Microtubule Interactome: Complexes with Roles in Cell Cycle and Mitosis

**DOI:** 10.1371/journal.pbio.0060098

**Published:** 2008-04-22

**Authors:** Julian R Hughes, Ana M Meireles, Katherine H Fisher, Angel Garcia, Philip R Antrobus, Alan Wainman, Nicole Zitzmann, Charlotte Deane, Hiroyuki Ohkura, James G Wakefield

**Affiliations:** 1 Department of Zoology, University of Oxford, Oxford, United Kingdom; 2 Wellcome Trust Centre for Cell Biology, University of Edinburgh, Edinburgh, United Kingdom; 3 Programa Doutoral em Biologia Experimental e Biomedicina, Center for Neuroscience and Cell Biology, University of Coimbra, Coimbra, Portugal; 4 Life Sciences Interface/Doctoral Training Centre, University of Oxford, Oxford, United Kingdom; 5 Department of Statistics, University of Oxford, Oxford, United Kingdom; 6 Department of Biochemistry, University of Oxford, Oxford, United Kingdom; University of Oxford, United Kingdom

## Abstract

The microtubule (MT) cytoskeleton is required for many aspects of cell function, including the transport of intracellular materials, the maintenance of cell polarity, and the regulation of mitosis. These functions are coordinated by MT-associated proteins (MAPs), which work in concert with each other, binding MTs and altering their properties. We have used a MT cosedimentation assay, combined with 1D and 2D PAGE and mass spectrometry, to identify over 250 MAPs from early *Drosophila* embryos. We have taken two complementary approaches to analyse the cellular function of novel MAPs isolated using this approach. First, we have carried out an RNA interference (RNAi) screen, identifying 21 previously uncharacterised genes involved in MT organisation. Second, we have undertaken a bioinformatics analysis based on binary protein interaction data to produce putative interaction networks of MAPs. By combining both approaches, we have identified and validated MAP complexes with potentially important roles in cell cycle regulation and mitosis. This study therefore demonstrates that biologically relevant data can be harvested using such a multidisciplinary approach, and identifies new MAPs, many of which appear to be important in cell division.

## Author Summary

The microtubule (MT) cytoskeleton is crucial for many aspects of cell function, including cell division. These functions are coordinated by MT-associated proteins (MAPs), which work in concert with each other, binding MTs and altering their properties. Although some MAPs have been identified, it is likely that many more exist. We have used the *Drosophila* embryo to identify novel MAPs. Our proteomic analysis identified over 250 proteins with the ability to bind MTs, approximately 100 of which had not previously been studied. We used RNA interference (RNAi) to deplete each of these novel proteins and found 21 new MAPs that regulate MT organisation. We also used a bioinformatics-based approach to identify putative MAP complexes. Finally, we focussed on two proteins, which we predicted to interact together with MTs during mitosis. We showed that these proteins do form a complex in embryos, colocalise to the MT organising centre (the centrosome) in cells, and function to regulate the number of centrosomes in a cell. This study therefore demonstrates the validity of using a multidisciplinary approach, and identifies new MAPs, many of which appear to be important in cell division.

## Abstract 3

The microtubule cytoskeleton is crucial for many aspects of cell function. A new multidisciplinary study identifies microtubule-associated proteins that are important in cell division.

## Introduction

The ability of a cell to grow, divide, and respond to environmental or developmental cues is orchestrated on many levels. In the postgenomic era, it is recognised that one such key organisational step lies in the formation and regulation of multiprotein complexes. The capacity of one protein to bind to and modify the function of another, through such diverse mechanisms as covalent modification, steric hindrance, protein bridging, or restriction of subcellular location, provides a level of control that individual proteins cannot achieve alone [1]. Recent high-throughput postgenomic technologies have opened the way to identify proteins and their interacting partners. Of these techniques, three complementary approaches are most widely used: yeast two-hybrid (Y2H) analyses, which allow the identification of potential binary protein interactions [2,3]; cellular proteomic purification techniques, coupled with mass spectrometry, which can be used to isolate intact protein complexes [4,5]; and RNA interference (RNAi) analysis, which provides functional information on individual proteins and their known interactors [6,7].

The organisation of the microtubule (MT) cytoskeleton by MT-associated proteins (MAPs) provides one example of how protein–protein interactions regulate function. MTs play diverse and important roles in many cellular processes. These dynamic protein polymers form from dimers of two related proteins, α- and β-tubulin, that exist in a soluble, cytosolic pool and that incorporate into existing MTs with predetermined polarity [8]. In most animal cells, MTs are nucleated from and anchored at an organising centre, the centrosome, which resides juxtaposed to the nuclear envelope. The intrinsic polarity of the MT can therefore be translated into subcellular positional information, allowing the efficient transport of materials from one location to another [9,10]. In addition, the ability of MTs to grow and shrink dynamically provides a single cell with the capacity to form multiple populations of MTs possessing different functional properties at a single point in time [11,12]. This elaborate temporal and spatial organisation of MT function within the cell is regulated, in a large part, by the proteins that interact with MTs, so-called MAPs. Although the term was originally applied to structural proteins isolated from axons, which bind to and stabilise MTs [13], MAPs can equally describe any protein that associates with MTs in vitro or in vivo, including MT motors and their cargoes [14], proteins that bind MT ends [15], or those that associate with MTs in a cell cycle–dependent manner [16,17].

Many MAPs elicit their effects as part of multiprotein complexes [18–21]. In addition, due to the ability of many MAPs to directly or indirectly affect MT dynamics during cell division, proteins that associate with MTs have been widely explored as potential anticancer targets [22]. We have taken a combinatorial approach to identify MAP complexes with roles in cell cycle regulation and mitosis, from the fruit fly Drosophila melanogaster. *Drosophila* embryos are an ideal tissue from which to isolate MTs and associated MAPs of this nature [23–26]. These syncytial embryos contain sufficient quantities of individual proteins to undergo multiple mitoses prior to zygotic transcription [27]. In addition, large quantities can be obtained with relative ease, and extracts made from this tissue possess high levels of mitotic kinase activity [28].

In this study, we initially combined a MAP purification technique based on MT cosedimentation [16] with mass spectrometry (MS). Our analysis identified 270 MAPs from early *Drosophila* embryos, 83 of which are encoded by previously uncharacterised genes. By subsequently combining a secondary RNAi screen with a bioinformatics analysis based primarily on Y2H data, we have identified a MAP complex containing the Skp1 homolog, SkpA, and a novel protein we term SkAP. We confirm that SkpA and SkAP associate with each other in *Drosophila* embryos, localise to centrosomes, and show that both proteins regulate centrosome duplication in vivo. Our approach is therefore able to identify novel MAP complexes with important roles in mitosis and cell cycle regulation.

## Results and Discussion

### Over 250 Proteins Associate with MTs in Early *Drosophila* Embryos

**Figure 1:**
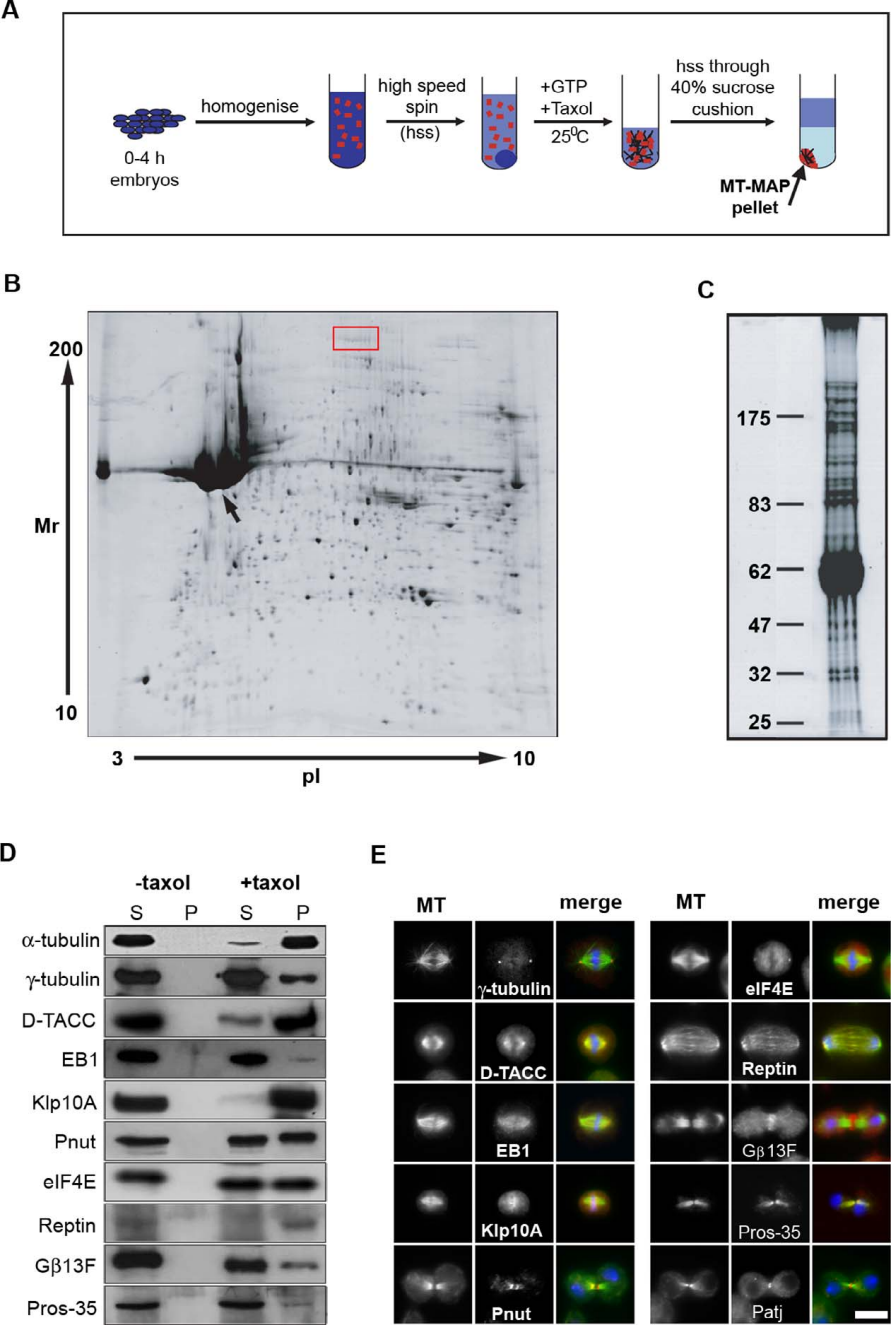
The MT Cosedimentation Assay, and Validation of MAPs (A) A representation of the methodology behind the assay (see text for details). Only MTs and their associated proteins pellet through the sucrose cushion; all other proteins remain in the supernatant. (B) 2D gel electrophoresis of the MT pellet. Approximately 880 features are visible in the 2D gel. Tubulin migrates as distinct features (e.g., arrow), which were avoided when choosing spots for mass spectrometry. In addition, we avoided cutting more than one feature from sets that clustered together in the same region of the gel (e.g., red box). (C) 1D gel electrophoresis of the MT pellet. Many distinct bands of varying molecular weight are seen. (D and E) Validation of the MAP hits. Antibodies were obtained to a selection of previously characterised proteins and tested for their ability to bind MTs in a cosedimentation assay (D) and to localise to MTs in S2 cells (E). Scale bar indicates 10 μm.

In order to identify MAPs from early *Drosophila* embryos, we made use of a well-characterised and previously described MT cosedimentation assay [16,25]. In this assay, a cytoplasmic extract of 0–4-h-old embryos is incubated at 25 °C in the presence of GTP to promote the polymerisation of endogenous tubulin. Subsequent addition of Taxol drives further MT polymerisation by lowering the critical concentration of MT growth, and irreversibly stabilises the MTs formed. By centrifuging this MT-enriched extract through a dense cushion of sucrose at 4 °C, MTs and MAPs pellet together, while other proteins remain in the supernatant (Figure 1A). The MT-MAP pellet is then solubilised and analysed by 2D or 1D gel electrophoresis (Figure 1B and 1C).

The 2D gradient gel analysis of the MT-MAP pellet identified approximately 880 features (Figure 1B). To ensure that the features present in the MT-MAP pellet were consistent between experiments, we repeated the cosedimentation assay on three separate occasions. In addition, we undertook a control experiment in which Taxol was excluded from the extract. Any MTs initially formed in this control extract depolymerise prior to centrifugation. Therefore, proteins present in the control pellet constitute contaminants. Such a control pellet contained approximately 200-fold less protein than the MT-MAP pellet, and identified only approximately 80 features (Materials and Methods; Figure S1A). Using customised image analysis software to compare individual experiments (Materials and Methods), and to exclude features present on the control gel, we identified 592 common MT-MAP features. As a complementary approach, we also analysed the MT-MAP pellet using 1D PAGE (Figure 1C). Although 2D PAGE provides much greater resolution of lower molecular weight proteins than 1D PAGE, it is not suitable for resolving high molecular weight proteins. In agreement with previous studies, we found many distinct MAP bands ranging in size from 25 kDa to greater than 250 kDa (Figure 1C). No features were visible by 1D gel electrophoresis when similar proportions of the control pellet were analysed (Figure S1B).

To determine the identities of the proteins cosedimenting with MTs from early embryo extracts, we used MS. Given the high number of features identified using 2D PAGE, we chose to analyse a total of 300 features on the basis of spot intensity. To minimise the likelihood of identifying posttranslationally modified forms of the same protein, we avoided cutting more than one spot from a cluster in the same region of the gel; one phosphorylation usually induces a pI shift of 0.2 units of pH towards the acidic end of the gel with almost no detectable change of molecular weight (e.g., red box, Figure 1B). In addition, we avoided the large tubulin features present at approximately 50 kDa (arrow, Figure 1B). In the 1D gel experiment, the lane was excised and cut into 31 blocks. Following reduction, alkylation, trypsin digestion, and peptide extraction of spots or blocks, the 2D and 1D protein mixtures were analysed using liquid chromatography–tandem mass spectrometry (LC-MSMS). Raw spectra were processed, and the peak lists searched in-house using Mascot Daemon 2.1. MS analysis of the 2D PAGE MT-MAP pellet identified 239 proteins, whereas 113 proteins were identified from the 1D PAGE gel blocks. Searching against a randomised database, we estimate the false-positive rate for the combined 2D gel spots and 1D gel LC-MSMS experiment to be 2.4%. By combining the two approaches, and after removal of redundant and low-scoring hits, we identified 270 potential MAPs from early *Drosophila* embryos (Table S1).

To initially validate the results of the cosedimentation assay and the MS, we chose ten MAPs for which antibodies were readily available and confirmed their ability to cosediment with MTs using Western blotting (Figure 1D). Additionally, we analysed their localisation in *Drosophila* S2 cells (Figure 1E). Whereas some of these were proteins already known to bind or localise to MT populations (e.g., D-TACC [25] and Klp10A [29]), others had not previously been shown to associate with MTs (e.g., eIF-4E, Reptin, and Gβ13F). We were able to confirm that nine of the ten proteins both bound to MTs in vitro and localised to MT structures in vivo (Figure 1D and 1E). In addition, although antibodies against the remaining protein, Patj, failed to recognise any specific bands on Western blots, they recognised central spindle and midbody MTs in S2 cells (Figure 1E). Therefore, we can be confident that our proteomic approach is able to identify previously unidentified MAPs.

### Proteins That Associate with MTs Have Diverse Cellular Functions

**Figure 2:**
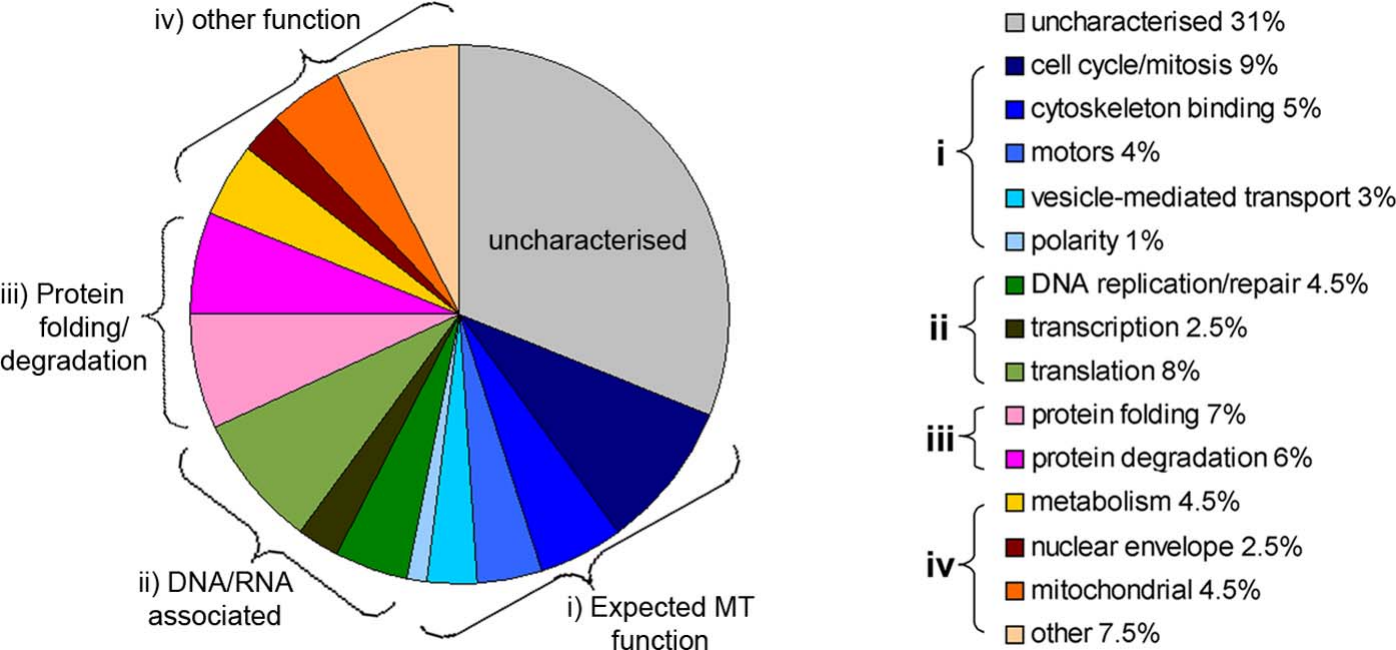
Functional Classification of 270 Drosophila Embryonic MAPs A pie chart showing the classification of identified MAPs according to Gene Ontologies, assisted by manual data mining of references in Flybase. A total of 31% of proteins were completely uncharacterised according to these sources. The remaining proteins were grouped by primary GO function and were then encompassed into four main categories: MT related (i), DNA/RNA associated (ii), protein folding/degradation (iii), and other (iv).

Although our approach did not detect some proteins previously shown to bind MTs in the early *Drosophila* embryo (e.g., CP60, Asp, and DmINCENP [30–32]), it identified many more proteins with the ability to bind MTs than anticipated [16]. To classify the 270 proteins on a functional basis, we first characterised the MAPs according to the Gene Ontology (GO) database [33] supported by additional manual data mining of previously published work (Figure 2; Table S2).

A total of 61 MAPs (22%) grouped into GOs that would be expected for MT-based functions, such as those involved in mitosis, cytoskeletal binding, vesicle-mediated transport, cell polarity, and MT motors (Figure 2; Table S2). Interestingly, although 126 MAPs (47%) do not have a primary ontology suggesting MT binding, there is evidence that many of these do, in fact, function in association with MTs. For example, four proteins classed within the “Translation” GO, eIF4E, Cup, Mat31B, and Aubergine have all been shown to be involved in the regulation of Oskar ribonucleoprotein (RNP) translation [34]. Components of this complex, which is essential for posterior patterning in the *Drosophila* oocyte, are localised in a MT-dependent manner, so it is not perhaps surprising that they associate with MTs in the early embryo. Some proteins, classed with “Other” functions, have also been shown to have roles in MT organisation during mitosis, although this is not described as their primary ontology (Table S2). For example, Calmodulin has recently been shown to directly bind the MAP Asp during mitosis, and RNAi against Calmodulin leads to defects in spindle morphology [35].

We also identified a number of MAPs for which there are currently no data suggesting an association with MTs in *Drosophila*, but that possess homologs that have been shown to localise to MT populations. For example, we identified many components of the nuclear pore complex. Of these, Ran was placed within the ontology “Mitosis/Cell Cycle” due to its clear role in chromatin-mediated MT nucleation [36]. However, other subunits, such as Pendulin (Importin α), Karyopherin β, fs(2) Ketel (Importin β), Nup358 (RanBP2), and Nup 214/CAN were placed within the “Nuclear Envelope” category. Nonetheless, homologs of these proteins have been shown to be components of the human mitotic spindle and to be involved in MT organisation and kinetochore function during mitosis [37–39]. Together with the localisation and biochemical data provided in Figure 1D and 1E, this analysis suggests that many other characterised proteins, which do not as yet possess ontologies linked to MTs, may also bind MTs in vivo. Importantly, it also demonstrates that our biochemical isolation of MAPs can preserve interactions between subunits of known protein complexes.

### RNAi Analysis of Novel MAPs

A total of 31% of MAPs identified in our cosedimentation assay were previously uncharacterised proteins. To investigate their cellular function, we used RNAi to deplete each of them from *Drosophila* S2 cells and examined MT organisation both in mitosis and interphase (Tables S3 and S4). Spindle abnormalities in mitotic cells were classified into one of the following categories: monopolar, bipolar with no asters, bipolar with one aster, tripolar, multipolar, or others; whereas interphase MT organisation was categorised into four classes: extended (normal), bundled, compact, and curved (Table S4). In total, from the 83 genes tested, 21 reproducibly gave a statistically significant difference from their control when treated with double-stranded RNA (dsRNA) (*p* < 0.05; Table 1, hits, and Table S4), 15 of which were scored with very high confidence (*p* < 0.01; Table 1, strong hits, and Table S4).

**Table 1:**
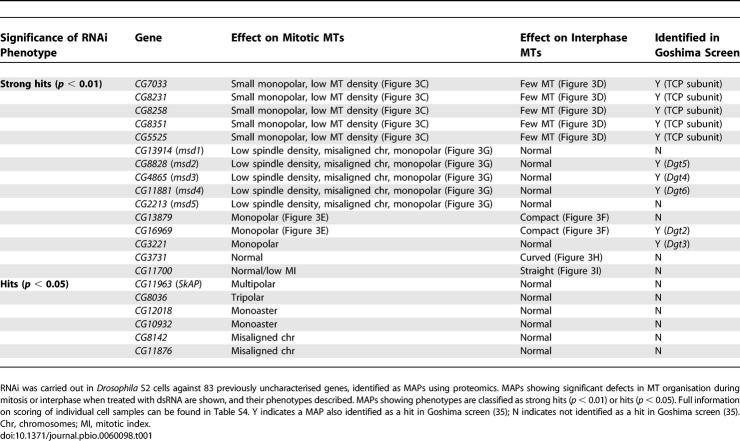
Table of Previously Uncharacterised MAPs Showing RNAi Phenotypes

**Figure 3:**
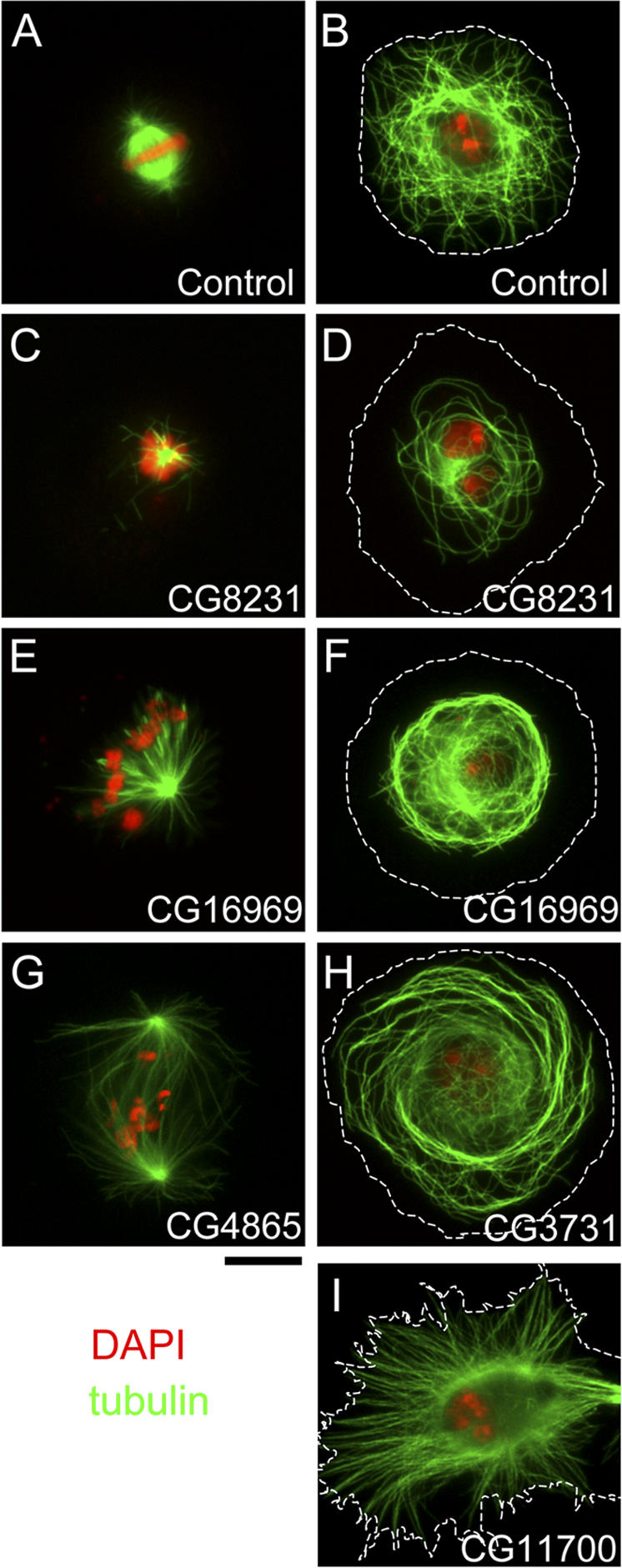
RNAi Phenotypes Associated with Previously Uncharacterised MAPs S2 cells were fixed with cold methanol and immunostained for α-tubulin (green), phospho-Histone H3 (unpublished data), and DNA (red), 5 d after being treated with dsRNA corresponding to each gene. A representative image of the phenotype observed in each class of gene identified in Table 1 is shown. (A, C, E, and G) mitotic cells. (B, D, F, H, and I) interphase cells. Dotted lines represent outlines of interphase cells defined by phase contrast observation. Scale bar indicates 10 μm.

Among the strong hits, five genes (*CG7033, CG8231, CG8258, CG8351*, and *CG5525*) showed monopolar spindles with reduced MT density in addition to generally low numbers of MTs in interphase cells, in relation to wild-type cells (Figure 3A–3D; unpublished data). Homology searches indicated that these genes encode proteins with a high similarity to the five subunits of TCP, a chaperonin complex for tubulins and actin [40] (unpublished data). The identification of all the *Drosophila* proteins with a high similarity to TCP subunits not only demonstrates the reliability of our RNAi analysis, but also provides further evidence to suggest that cosedimentation of one component of a complex correlates with cosedimentation of additional subunits.

RNAi against three other genes (*CG13879*, *CG16969*, and *CG3221*) showed monopolar spindles without apparent reduction of MT density (Figure 3E; unpublished data). In addition, although cells treated with dsRNA against *CG3221* showed no defect in interphase MT organisation (unpublished data), MT organisation in interphase was abnormal in both *CG13879* and *CG16969* dsRNA-treated cells (Figure 3F; unpublished data). In these cells, MTs stayed compacted around the nucleus, in contrast to control cells in which plus ends of interphase MTs extend to the cell periphery. A further five strong hits (*CG13914*, *CG4865*, *CG8828*, *CG11881*, and *CG2213*) led to large bipolar spindles with reduced MT density, misaligned chromosomes, and an increase in monopolar spindles (Figure 3G; unpublished data). Interestingly, neither astral nor interphase MT organisation was affected. We have termed these five genes *msd*1–5 (*mitotic spindle density* 1–5). Finally, two other genes (*CG3731* and *CG11700*) showed altered organisation of interphase MTs without significant abnormalities of the mitotic spindle (Figure 3H and 3I).

Very recently, a *Drosophila* genome-wide RNAi screen in which S2 cells were examined for defects in mitotic spindle morphology has been reported [35]. Although methodology and scoring of hits differed between that screen and ours, we found significant overlap between the studies. Of the 83 uncharacterised MAPs identified here, the Goshima screen positively identified ten with RNAi phenotypes, all of which we independently recorded as strong hits (Tables 1 and S4). These include the five subunits of the TCP complex, three of our *msd* genes (*CG8828/msd2*, *CG4865/msd3*, and *CG11881/msd4*, described in the Goshima screen as *Dgt5*, *Dgt4*, and *Dgt6*, respectively), *CG16969* (*Dgt2*), and *CG3221* (*Dgt3*). Additionally, we identified another five strong hits, three of which had mitotic phenotypes (*CG13914/msd1*, *CG2213/msd5*, and *CG13879*), and two which appear to affect only interphase MTs (*CG3731* and *CG11700*). We undertook RNAi against 83 genes, and identified 19 that possess defects in mitotic spindle organisation, 13 of which we classify as strong hits (corresponding to a 16% hit rate). In contrast, the Goshima screen undertook RNAi against 14,425 genes and identified 205 genes (corresponding to a 1.4% hit rate). Thus, it is clear that our targeted RNAi screen, based on the ability of proteins to physically associate with MTs, substantially increases the proportion of genes identified as having roles in spindle organisation, in comparison to a genome-wide RNAi screen.

### Bioinformatics Analysis of Binary MAP Interactions

Although our RNAi analysis identified additional individual proteins involved in mitotic MT organisation, it did not provide information regarding how these proteins work in concert with others to elicit their cellular functions. One of the most powerful technologies used for mapping binary protein interactions is the Y2H system, and screens using this technique have been carried out in many organisms, including yeast, *Drosophila*, and humans [2,41,42]. We took advantage of the GRID database [43] which, when supplemented with the small number of functional interactions stored in Flybase, gives an approximate *Drosophila* interactome of 24,700 interactions. Computer algorithms were constructed to identify binary interactions within the MAP dataset.

**Figure 4:**
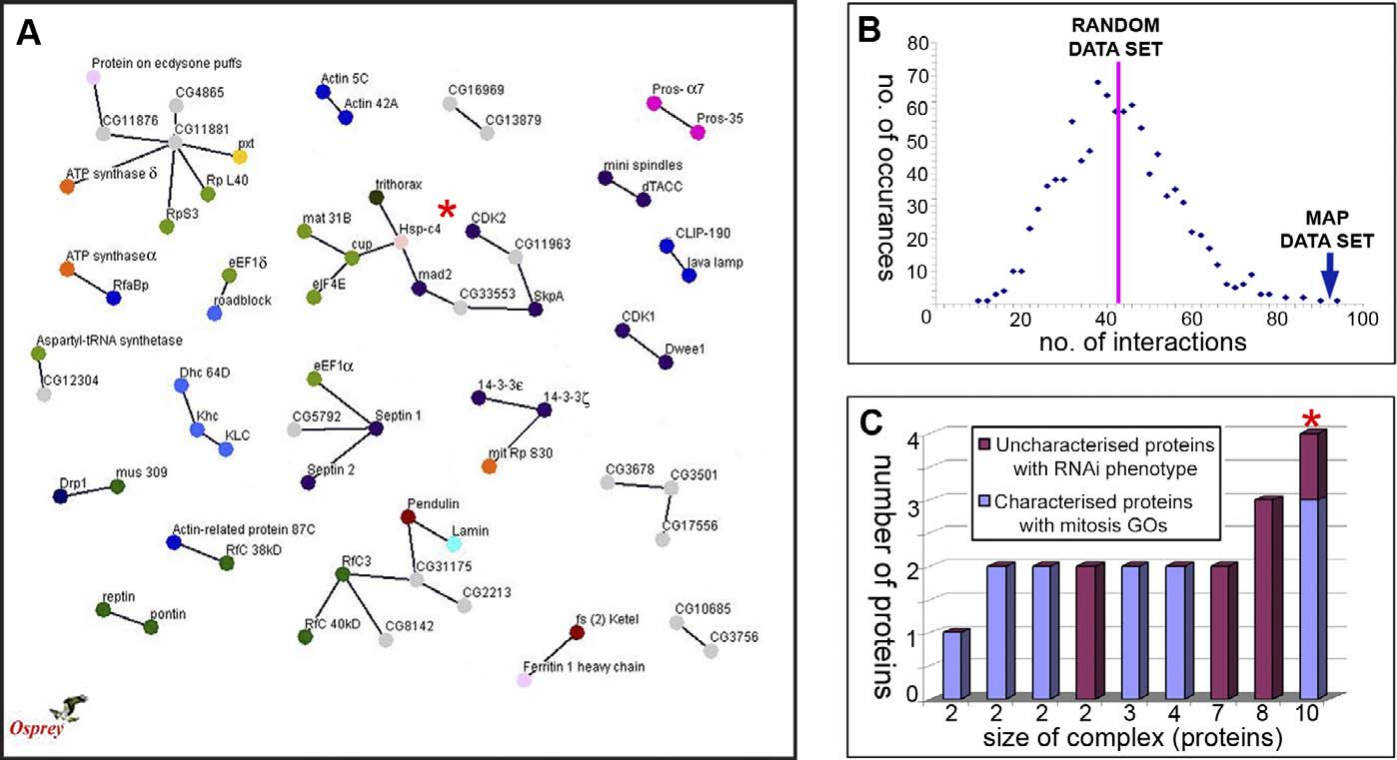
Pairwise Interactions of Identified MAPs (A) A total of 66 of the 270 MAPS show direct pairwise interactions with another MAP, gathered from GRID and Flybase; displayed here as an interaction network. Proteins are indicated by circular nodes and interactions by lines, or edges. (B) The distribution of interactions within randomly selected sets of proteins. The average number of interactions is 42.71 (s.d. ± 13.09; 1,000 repeats). The MAP set shows 92 interactions, 3.77 standard deviations away from the mean of the distribution. (C) A bar chart representing the size and compositions of complexes in (A). Any complex containing either characterised mitotic/cell cycle proteins, or uncharacterised proteins showing an RNAi phenotype are represented here. The largest putative complex (indicated by an asterisk [*]) contains both classes of proteins.

Of our 270 MAPs, in-house computer algorithms identified interaction data for 216. An analysis of the binary protein interactions between these proteins identified 66 MAPs, contributing to 92 interactions (counting both “A–B” and “B–A”) within 21 putative complexes (Figure 4A). As all of these proteins were originally isolated on the basis of their association with MTs, we would expect binary interactions to occur between the MAPs with a higher frequency than between a set of randomly selected proteins. To test this hypothesis, computer algorithms were devised to randomly select a set of 216 *Drosophila* proteins obtained from Flybase, and count the pairwise interactions of that set. This analysis was repeated 1,000 times to obtain a distribution of interactions (Figure 4B). On average, a random set of 216 proteins showed 42.7 binary interactions (standard deviation [s.d.] ± 13.1). The 92 interactions within our MAP dataset falls 3.8 standard deviations away from this mean. We therefore conclude that the MAPs isolated in this study do indeed interact with one another more often than a set of random, unrelated proteins (*p* < 0.002), suggesting that many of the interactions described using Y2H approaches represent valid, functional interactions.

To investigate the existence of MAP complexes that function in cell cycle regulation or mitosis in vivo, we combined the information regarding protein function obtained from the GOs of previously characterised proteins and that obtained from our targeted RNAi screen, with the binary MAP interactions highlighted by the bioinformatics analysis above. The combined data are represented as a bar chart in Figure 4C. Many of the putative complexes obtained from the bioinformatics analysis included individual proteins shown to have a mitotic/cell cycle–related function (blue), or proteins encoded by previously uncharacterised genes that showed defects in MT organisation in our RNAi analysis (dark red). However, one putative complex included both: three proteins with known mitotic/cell cycle function, two of which directly interacted with a protein encoded by a gene that gave a significant defect in our RNAi analysis (Figure 4A and 4C; asterisk). We therefore decided to validate our approach by concentrating on the protein encoded by this previously uncharacterised gene, *CG11963*, and its interacting partners.

### Verification of a MAP Complex with a Role in Regulating Centrosome Number

*CG11963* encodes a predicted 55-kDa protein, with homology to a human mitochondrial protein, Succinate-CoA ligase (Figure S2) [44]. However, depletion of CG11963 in our RNAi analysis consistently led to a statistically significant effect on mitotic spindle organisation during mitosis in S2 cells (Tables 1 and S4). In addition, our analysis of the extant interaction data placed the MAP encoded by *CG11963* as able to directly interact with SkpA and Cdc2c (CDK2) (Figure 4A).

**Figure 5:**
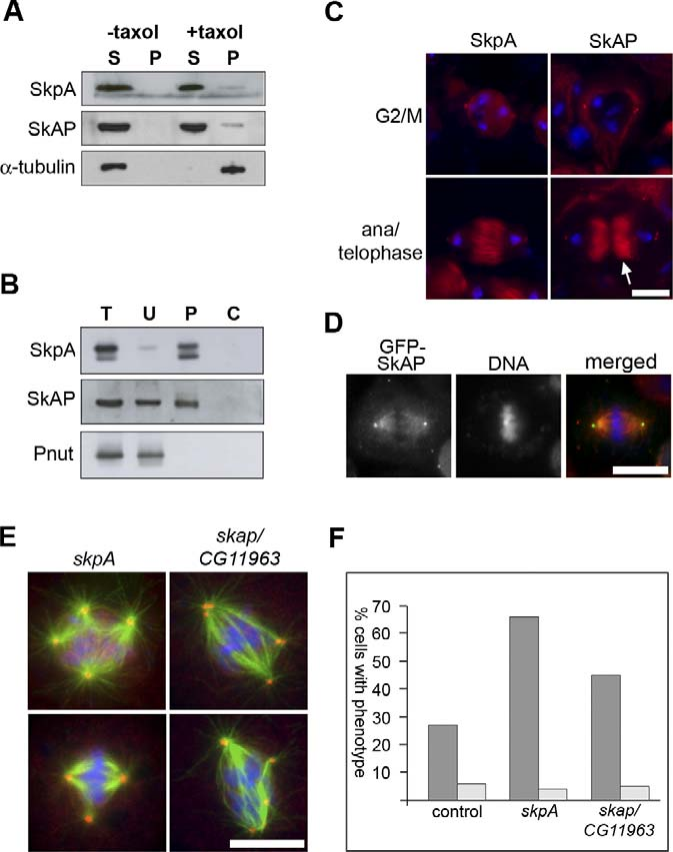
SkpA and SkAP Are Centrosomal MAPs That Form a Complex In Vivo, and Regulate Centrosome Number (A) An embryonic MT cosedimentation assay probed with antibodies to SkpA and SkAP. A fraction of both proteins are present in the MT pellet in the presence of Taxol. α-Tubulin is shown as a control. (B) Immobilised anti-SkpA antibodies were used to precipitate SkpA from 0–4-h embryo extracts. SkAP, but not a control protein, Pnut, coprecipitates with SkpA. C, control precipitate; P, bound precipitate; T, total embryo extract; U, unbound supernatant. (C) Localisation of SkpA and SkAP in meiotic spermatocytes. Both SkpA and SkAP are present at centrosomes throughout the cell cycle. DNA is shown in blue, and either SkpA or SkAP is shown in red. SkpA is additionally present on the nuclear envelope and on the central spindle MTs during anaphase and telophase. In addition to centrosomes, SkAP is found on mitochondria, which aggregate around the central spindle during telophase (arrow). (D) An S2 cell in metaphase, expressing GFP-SkAP. The fusion protein accumulates at centrosomes. Merged; DNA (blue), MTs (red), GFP-SkAP (green). (E) S2 cells treated with dsRNA against *skpA* or *skap/CG11963*. Cells were fixed with methanol and stained to visualise MTs (green), DNA (blue), and centrosomes (red). RNAi against either gene results in supernumerary centrosomes. (F) A bar chart representing the percentage of mitotic cells showing an increase in centrosome number (i.e., more than two centrosomes per cell; dark grey), and cells showing defects in cytokinesis (i.e., binucleate cells; light grey) when treated with control dsRNA, or dsRNA against *skpA* or *skap/CG11963*. No increase in binucleate cells is seen, demonstrating that the increase in centrosome number is not due to failure of cytokinesis. Scale bar indicates 10 μm.

To validate the existence of this putative complex in vivo, we produced and affinity purified antibodies against all three proteins. Although antibodies against CDK2 failed to recognise specific bands on Western blots, antibodies against SkpA and CG11963 each recognised bands of the predicted M_r_ in both embryo extracts and S2 cells (Figure S3A and S3B). We therefore refer to the gene product of *CG11963* as SkAP (SkpA Associated Protein). To confirm that SkpA and SkAP are bone fide MAPs, we performed a MT cosedimentation assay using 0–4-h *Drosophila* embryo extracts, and probed the samples by Western blotting. As expected, a fraction of both SkpA and SkAP were present in the Taxol-stabilised MT pellet (Figure 5A). Next, we immunoprecipitated SkpA from 0–4-h embryo extracts and looked for coprecipitation of SkAP. Immunodepletion of SkpA from extracts led to coprecipitation of approximately half the endogenous SkAP, while having no effect on control proteins present in the extract (Figures 5B and S3C).

Previous studies on the vertebrate homolog of SkpA, Skp1, have shown the protein to localise to the centrosome and the midbody [45–47]. In agreement, our affinity-purified anti-SkpA antibodies recognised centrosomes and central spindle MTs in both male meiotic cells and larval neuroblasts (Figures 5C and S3D). In addition, SkpA was present on the nuclear envelope prior to meiosis (Figure 5C). Similarly, anti-SkAP antibodies recognised centrosomes in these tissues (Figures 5C and S3E), as well as staining mitochondria, which line up along the length of the central spindle during meiotic anaphase and telophase [48] (arrow, Figure 5C). To further confirm the localisation of SkAP, we expressed a GFP-SkAP fusion protein in S2 cells, and found it to accumulate at centrosomes (Figure 5D). Thus, both SkpA and SkAP colocalise at centrosomes in vivo.

Finally, we investigated whether SkpA and SkAP fulfil similar functions in cells. Our initial RNAi analysis had shown that reduction of *skap/CG11963* led to a significant defect in spindle morphology (Tables 1 and S4). In addition, perturbation of Skp1/SkpA function has previously been shown to result in a mitotic phenotype (that of supernumerary centrosomes) in a variety of organisms, including *Drosophila* [46,49,50]. We therefore compared the phenotypes of S2 cells in which either *skpA* or *skap/CG11963* had been knocked down, staining cells to visualise MTs, DNA, and centrosomes (Figure 5E and 5F). We found that cells treated with dsRNA to either *skap/CG11963* or *skpA* had a clear supernumerary centrosome phenotype (45% of *skap* dsRNA-treated cells and 66% of *skpA* dsRNA-treated cells contained more than two centrosomes, in comparison to 27% in control-treated cells). Interestingly, the proportion of binucleated cells was similar between control, *skap* dsRNA-treated, and *skpA* dsRNA-treated cells, suggesting the supernumerary centrosomes arise through an increase in centrosome duplication, rather than failures in cytokinesis (Figure 5F; Table S4). Taken together, our results show that *Drosophila* SkpA and a previously uncharacterised protein SkAP, are centrosomal MAPs that associate in vivo and have a role in regulating the number of centrosomes in cells.

### Conclusions

We began this study by undertaking a biochemical purification and proteomic analysis in order to identify proteins that associate with the MT cytoskeleton. Our initial purification of MAPs from *Drosophila* embryo extracts identified many more proteins with the ability to bind MTs than anticipated and provided a wealth of possible routes of further study. The subsequent RNAi screen, and the investigation of binary protein interaction data were used as functional sieves, focussing our studies on putative MAP complexes with roles in cell cycle regulation and cell division. The biochemical and cytological data relating to SkpA and SkAP validate combining these various approaches; these proteins do, indeed, form a complex in embryos, and their similar localisations and phenotypes upon RNAi treatment strongly suggest that they function together in the cell.

In the postgenomic era, assigning function to the many thousands of uncharacterised genes for which there is little or no experimental data solicits a challenge that no single scientific discipline can meet. However, by combining biochemistry, proteomics, and functional RNAi, with bioinformatics, statistics, and targeted data mining, it is clear that useful biological data can be acquired. We believe that this interdisciplinary-style approach will increasingly contribute to the biological knowledge base, and greatly facilitate the transition from genomic to functional information.

## Materials and Methods

### Fly work and embryo collection.

All flies were reared according to standard procedures and maintained at 25 °C. The 0–4-h-old embryos were collected on apple juice/Agar plates from collection chambers containing OrR *Drosophila* stocks. Embryos were dechorionated using bleach, washed in PBS, flash frozen in liquid nitrogen. and stored at −80 °C in batches.

### MT cosedimentation assay.

We modified a standard protocol for a *Drosophila* embryonic MT cosedimentation assay, described in [25] and originally adapted from [16]. Briefly, 2 g of frozen embryos were homogenised in 3 ml of C buffer (50 mM HEPES [pH 7.4], 50 mM KCl, 1 mM MgCl_2_, 1 mM EGTA, 0.1% NP-40, protease inhibitors [Sigma]). Extracts were clarified by an initial centrifugation at 15,000 *g* for 10 min, followed by two consecutive high-speed spins at 100,000 *g*; one for 45 min and the second for 15 min. Clarified supernatant was transferred to fresh tubes between each spin. GTP and DTT were each added to the supernatant to a final concentration of 1 mM before incubation at 25 °C for 5 min. The supernatant was then divided into two equal fractions. One was placed on ice, and to the other, Taxol was added to 10 μM (final concentration), before returning to 25 °C for a further 15 min. The supernatants were layered onto a two-volume cushion of C buffer + 40% sucrose, before centrifuging at 100,000 *g* for 30 min at 4 °C. The supernatant was removed by aspiration, and the top surface of the sucrose cushion was washed twice with C buffer. The remaining sucrose cushion was then removed by aspiration, and the pellet washed once with C buffer before being resuspended in protein sample buffer (5 M urea, 2 M thiourea, 2 mM tributyl-phosphine, 65 mM DTT, 65 mM CHAPS, 0.15 M NDSB-256, 1 mM sodium vanadate, 0.1 mM sodium fluoride, and 1 mM benzamidine).

### 1D and 2D PAGE and mass spectrometric analysis.

Carrier ampholytes were added to the MT-MAP sample at 0.9 % v/v Servalyte 3–10, 0.45 % (v/v) Servalyte 2–4 and 9–11 prior to IEF on 3–10 nonlinear pH gradient gels. The 2D gel electrophoresis, and internal calibration of the 2D gel images with regard to pI and molecular weight were carried out as described previously [51]. Following electrophoresis, the gels were fixed in 40% v/v ethanol/10% v/v acetic acid and stained with the fluorescent dye OGT MP17 as described previously [52]. The 16-bit monochrome fluorescence images were obtained at 200-μm resolution by scanning gels with an Apollo II linear fluorescence scanner, and images were processed with a custom version of Melanie II (Bio-Rad Laboratories).

In-gel digestion of features selected for mass spectrometric analysis and the corresponding peptide extraction were carried out as described previously [50]. Mass spectrometric analysis was carried out using a Q-TOF 1 (Micromass) coupled to a CapLC (Waters). Tryptic peptides were concentrated and desalted on a 300-μm id/5-mm C18 precolumn and resolved on a 75-μm id/25-cm C18 PepMap analytical column (LC packings). Peptides were eluted to the mass spectrometer using a 45-min 5%–95% acetonitrile gradient containing 0.1% formic acid at a flow rate of 200 nl/min. Spectra were acquired in positive mode with a cone voltage of 40 V and a capillary voltage of 3,300 V. The MS to MS/MS switching was controlled in an automatic data-dependent fashion with a 1-s survey scan followed by three 1-s MS/MS scans of the most intense ions. Precursor ions selected for MS/MS were excluded from further fragmentation for 2 min. Spectra were processed using ProteinLynx Global server 2.1.5 and searched against the SWISS-PROT, MSDB, and NCBI databases using the MASCOT search engine (Matrix science). Searches were restricted to the *Drosophila* taxonomy, allowing carbamidomethyl cysteine as a fixed modification and oxidised methionine as a potential variable modifications. Data were searched allowing 0.5 Da error on all spectra and up to two missed tryptic cleavage sites to accommodate calibration drift and incomplete digestion. A standard score, based upon a number of criteria including number and size of peptides matched, was assigned to each spectra. Proteins identified with a score of greater than 30 were considered significant, whereas all lower-scoring proteins were either included or discarded after inspection of individual spectra. This resulted in the inclusion of seven additional proteins with scores of between 26.87 and 29.36 (see Table S1). Where peptides from the same 2D feature or 1D gel block resulted in two possible identities of similar scores, both possibilities were included in our final count and are differentiated by a numerical subfix (see Figure S1). Data were checked for consistent error distribution, and false-positive rates were determined by searching against a randomized database.

### Cell culture, RNA interference, and transfection.

S2 cells were treated with dsRNA corresponding to each gene or a negative control (bacterial β-lactamase) as previously described (Table S3) [53]. Aliquots of cells were removed 3 and 5 d after dsRNA treatment, and adhered on coverslips coated with concanavalin A (Sigma). The cells were then fixed with −20 °C methanol and immunostained for DNA, α-tubulin, and a mitotic marker, phospho-H3 (Ser^10^). As even untreated S2 cells show abnormalities to some extent, all scoring and statistical analysis compared each gene with the negative control (β-lactamase dsRNA) in the same experiment. The mitotic index (the frequency of phospho-H3–positive cells) was calculated for each sample (*n* = ∼500 cells per sample). All mitotic cells with spindle abnormalities were classified into one of the following categories: monopolar, bipolar with no asters, bipolar with one aster, tripolar, multipolar, or others (*n* = ∼60 mitotic cells per sample). Abnormal chromosome distribution was also scored but only when the spindle was bipolar. Interphase microtubule organisation was categorised into four classes: extended (normal), bundled, compact, and curved. Any other abnormalities were noted separately (Table S4).

The χ-square test was used for statistical analysis. The frequency of abnormalities for each gene was individually compared to that of the control on the same experiment either in each individual category or categories combined. Genes which reproducibly give *p*-values lower than 0.05 were considered hits, and ones which give *p*-values lower than 0.01 were considered strong hits.

### Bioinformatics analysis.

MS identities were received in the form of database accession numbers. In-house Perl script was used to match identities to Celera Gene names (CG number) for further use. Databases used were SwissProt: http://www.uniprot.org, NCBI: http://www.ncbi.nlm.nih.gov, and MSDB: http://www.matrixscience.com. Redundant and incorrect hits (matches to Drosophila pseudoobscura) were removed. Proteins encoded by genes were classified according to their function at http://www.geneontology.org [33] supported by manual data mining of references in Flybase (http://www.flybase.org). Uncharacterised proteins were those that have not been investigated previously. Protein–protein interaction data were downloaded from http://www.thebiogrid.org [43] (April 1st, 2007, release) and supplemented with interactions stored in http://www.flybase.org. Redundancy and self interactions were removed, giving 24,700 pairwise interactions, consisting mainly of Y2H interactions. All networks were visualised using Osprey Network Visualisation Tool (v1.2.1) [54]. To determine whether the MAP set contained more direct pairwise interactions than a group of randomly selected proteins, in-house Perl script was used to construct a random set of *Drosophila* proteins and count the pairwise interactions within that set. A total of 1,000 repeats were run to achieve and plot a distribution of the data.

### Antibody production and purification.

Full-length *Drosophila skpA* and *CG11963/skap* were amplified using cDNA obtained from 0–4-h embryos, and subcloned into pMal-c2x (Invitrogen) using standard procedures. Purified MBP-SkpA and MBP-SkAP were then used to generate polyclonal rabbit antisera by Eurogentec (Seraing). Antibodies were affinity purified from the resultant rabbit antisera using a column of the appropriate His-tagged fusion protein immobilised onto Affigel-15 (BioRad).

### Immunostaining and microscopy.

*Drosophila* S2 cells were adhered on coverslips coated with concanavalin A and fixed with either 4% formaldehyde for 10 min, or with −20 °C methanol for 15 min, prior to processing for immunofluorescence. To visualise SkpA and SkAP localisation, testes and larval neuroblasts were fixed according to [55] and [56], respectively. Samples were processed for immunofluorescence by incubating in blocking buffer (0.1% PBST + 3% BSA) for 30 min, prior to incubation with the appropriate primary and secondary antibodies.

To monitor localisation of SkAP in vivo, full-length CG11963 was cloned into the Gateway expression vector pAGW (*Drosophila* Genome Resource Center) via pENTR/D/TOPO (Invitrogen), and transfected into *Drosophila* S2 cells using cellfectin (Invitrogen) as per manufacturer's instructions. After 48 h, cells were placed on concanavalin A–coated coverslips, fixed with 4% formaldehyde, and costained for DNA and α-tubulin.

The following antibodies were used at 1:500: mouse anti–α-tubulin (DM1A clone; Sigma), rat anti–α-tubulin (Jackson Laboratories), mouse anti–γ-tubulin (Sigma), rabbit anti–D-TACC (a gift from Jordan Raff), rabbit anti-EB1 (a gift from Ron Vale), rabbit anti-Klp10A (a gift from David Sharp), rabbit anti-Pnut (to be described elsewhere), rabbit anti-eIF4E (a gift from Akira Nakamura), rabbit anti-Gβ13F (a gift from Fumio Matsuzaki), rabbit anti–Pros-35 (a gift from Peter Kloetzel), rabbit anti-Pontin (a gift from Jacques Pradel), rabbit anti-Patj (a gift from Hugo Bellen), guinea pig anti-SkpA (a gift from Bob Duronio), anti–phospho-Histone H3 Ser^10^ (Upstate Biotech), and rabbit anti-SkpA and rabbit anti-SkAP (described above). Appropriate Alexa 488, Cy3, and Cy5 secondary antibodies were obtained from Molecular Probes or Jackson Laboratories. DNA was visualized with Vectashield containing DAPI (Vectorlabs).

Preparations were examined under oil at 25 °C with a Nikon Eclipse TE2000-U with Nikon Plan APO VC 60× 1.4N/A objective, with 1.5× integrated zoom, using a Hamamatsu c8484–056 camera. Pictures were captured using IPlab software, converted to TIFF files, pseudocoloured, and merged in Adobe Photoshop CS2. Levels of individual channels were adjusted where applicable to maximise pixel range.

### Immunoprecipitation and Western blotting.

Clarified extracts of 0–4-h *Drosophila* embryos homogenised in C buffer, were incubated with affinity-purified anti-SkpA or anti-Pnut antibodies immobilised using the Profound Co-immunoprecipitation kit (Pierce), or with control beads in the absence of antibodies, according to manufacturer's instructions. Immunoprecipitated samples were resuspended in Pierce sample buffer and subjected to standard 1D SDS-PAGE and Western blotting. Blotted membranes were probed with affinity-purified antibodies to SkpA, SkAP, and Pnut at 1:1,000. MT cosedimentation assays were analysed by Western blotting and probed with appropriate antibodies (1:1,000; see “Immunostaining and Microscopy” in Materials and Methods for antibodies used).

## Supporting Information

Figure S1 2D and 1D Polyacrylamide Gels of a Control MT Cosedimentation Assay A MT cosedimentation assay was performed in the absence of Taxol and the “pellet” solubilised in protein sample buffer. When proportions of the pellet, similar to those used for the MS of the pellet in the presence of Taxol, were subjected to 2D gel electrophoresis, approximately 80 features were visible (A).
No features were visible in the control pellet when 1D gel electrophoresis was used (B). (S) control supernatant, (P) control pellet. The control 2D gel was superimposed onto the MT cosedimentation gel using a custom version of Melanie II software (BioRad). Any coinciding features were avoided when choosing spots for mass spectrometry.
(1.36 MB TIF)

Figure S2 CG11963/SkAP Shares Homology to Succinate-CoA Ligase (A) Diagrammatic representation of CG11963/SkAP, showing a putative mitochondrial targeting sequence at the N-terminus (orange) (mitoprot prediction; http://ihg.gsf.de/ihg/mitoprot.html), and the conserved ATP-grasp (blue) and CoA-ligase domains (green) found in Succinate-CoA ligase family members. Note the presence of a highly charged C-terminal extension in CG11963/SkAP (red).
(B) Alignment of CG11963/SkAP protein sequence with Succinate CoA ligase family members using ClustalW. Black stars indicate conserved residues. Orange residues indicate predicted mitochondrial targeting sequence. Red stars indicate residues conserved from mammals through to Escherichia coli within the nucleotide binding domain [44].
(2.27 MB TIF)

Figure S3 Characterisation of Anti-SkpA and Anti-SkAP Antibodies (A) Full-length Western blots of *Drosophila* embryo extracts probed with affinity-purified rabbit anti-SkpA and anti-SkAP antibodies. The antibodies recognise bands of the predicted molecular weight. In addition, the SkpA antibody recognises a band of slightly lower molecular weight and at lower intensity.
(B) The 0–4-h embryo extract treated with λ phosphatase buffer (Con) or phosphatase buffer in the presence of λ phosphatase (Ptase) for 30 min at 37 °C, prior to Western blot analysis using the anti-SkpA antibody. The bands recognised by the antibodies do not resolve, suggesting they are not differentially phosphorylated forms of SkpA.
(C) A control immunoprecipitation to show the specificity of the anti-SkpA immunoprecipitation described in Figure 5. Immobilised anti-Pnut antibodies were used to precipitate Pnut from 0–4-h embryo extracts. Neither SkAP nor SkpA coprecipitate with Pnut. C, control precipitate; P, bound precipitate; T, total embryo extract; U, unbound supernatant.
(D and E) Localisation of SkpA (D) and SkAP (E) in larval neuroblasts. Cells were fixed according to [53] and stained to visualise DNA (blue), MTs (green), and either SkpA or SkAP (red). Both proteins localise to centrosomes throughout the cell cycle. Scale bar indicates 10 μm.
(1.41 MB TIF)

Table S1 List of 270 Putative MAPs, Including Mass Spectrometry Peptide Sequence Identification The table includes all 270 proteins identified in the MT cosedimentation assay, listing the CG number, synonyms, SWISS-PROT–calculated molecular weight(s), the experiment in which it was identified (i.e., 1D or 2D analysis), and the peptide sequences and scores associated with the positive identification. Proteins identified with a score of greater than 30 were considered significant, whereas all lower-scoring proteins were either included or discarded after inspection of individual spectra. This resulted in the inclusion of seven additional proteins with scores of between 26.87 and 29.36. Each individual hit was been assigned a number and grouped into functional classifications, by GO, for ease of cross reference (Figure 2; Table S2). In a small number of cases, a peptide, or set of peptides, matched to more than one possible protein. In the table, these proteins have been assigned a shared number, but are differentiated by a letter. Therefore, although 270 potential MAPs were identified, these are numbered from 1–257. Where duplicated peptide sequences span more than one functional grouping, a star is shown next to the number.
(620 KB DOC)

Table S2 Table of 270 Hits, Classified in Functional Groups, According to Gene Ontology (GO) All 270 MAPs were classified into functional groups according to GO (Figure 2). Each number assigned relates to those numbers given in Table S1. Where a protein possesses more than one GO, the primary functional GO based on mutational analysis was used. Each GO code and associated descriptions are listed to show the justification for assignment of a particular functional group.
(351 KB DOC)

Table S3 List of Primers Used for Generation of dsRNA The table includes all the primers used for dsRNA generation. CG numbers are indicated along side with a number that relates to those given in Table S1. More than one set of primers for genes are indicated when the phenotype was rechecked or when more than one transcript was known to exist.
(139 KB DOC)

Table S4 Raw Data of Scored RNAi Phenotypes for Strong Hits and Hits The initial RNAi data collection for previously uncharacterised MAPs showing a phenotype based on the categories stated. Only the data for strong hits and hits are shown. All comparisons were made in relation to the negative control (β-lactamase dsRNA) present in a particular experiment. Each RNAi experiment was subsequently repeated for both strong hits and hits, in order to confirm the phenotype (data not shown).
(255 KB DOC)

## Abbreviations

dsRNA: double-stranded RNA
GO: gene ontology
MS: mass spectrometry
MAP: microtubule-associated protein
MT: microtubule
RNAi: RNA interference
Y2H: yeast two-hybrid
