# Supplementary figures and images for: A Microtubule Interactome: Complexes with Roles in Cell Cycle and Mitosis
Source: PLoS Biol. 2008 Apr 22;6(4):e98. doi: 10.1371/journal.pbio.0060098 (PMC2323305; doi:10.1371/journal.pbio.0060098)

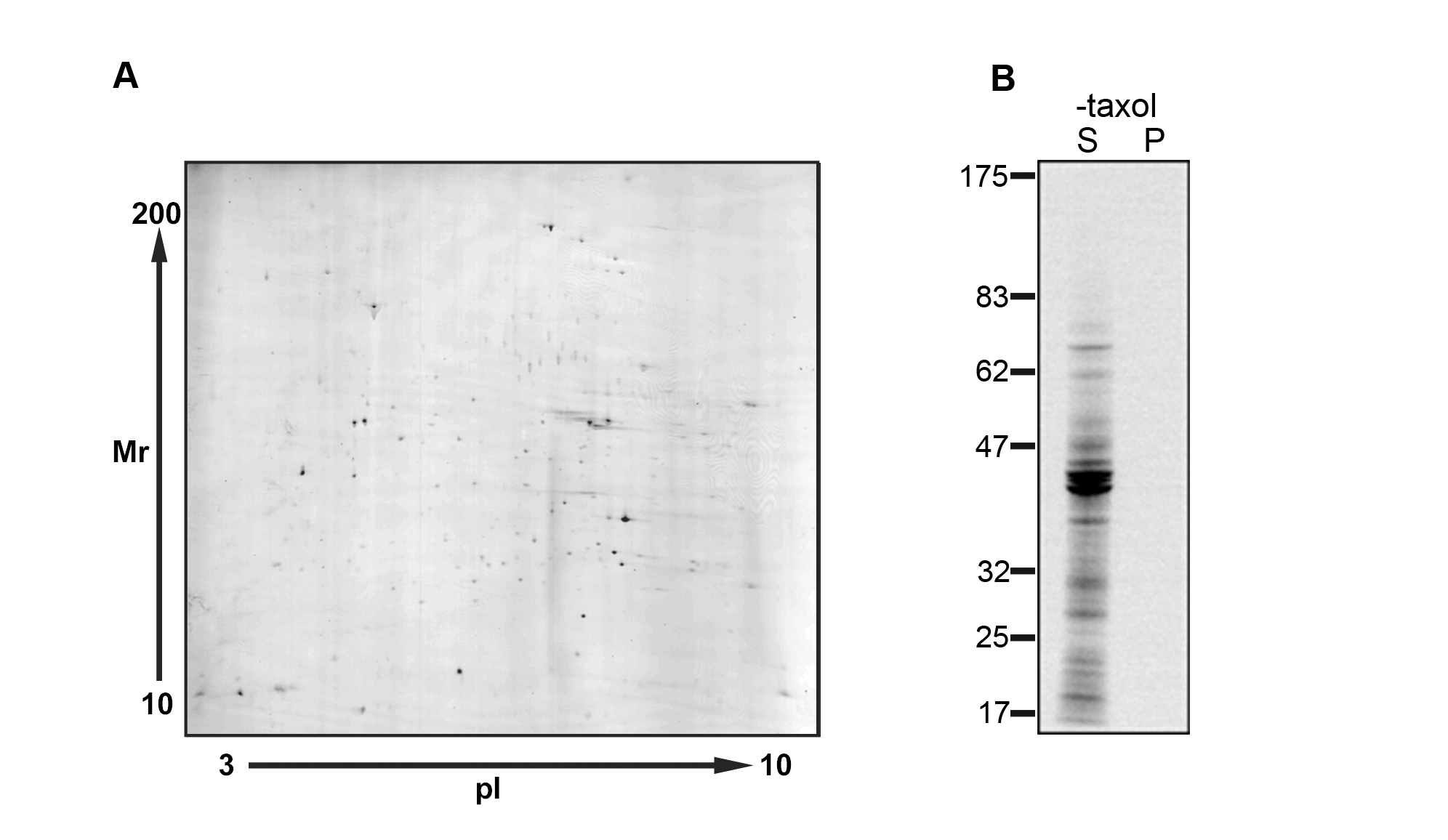

Supplement: Figure S1 — A MT cosedimentation assay was performed in the absence of Taxol and the “pellet” solubilised in protein sample buffer. When proportions of the pellet, similar to those used for the MS of the pellet in the presence of Taxol, were subjected to 2D gel electrophoresis, approximately 80 features were visible (A). No features were visible in the control pellet when 1D gel electrophoresis was used (B). (S) control supernatant, (P) control pellet. The control 2D gel was superimposed onto the MT cosedimentation gel using a custom version of Melanie II software (BioRad). Any coinciding features were avoided when choosing spots for mass spectrometry. (1.36 MB TIF) [file pbio.0060098.sg001.tif]

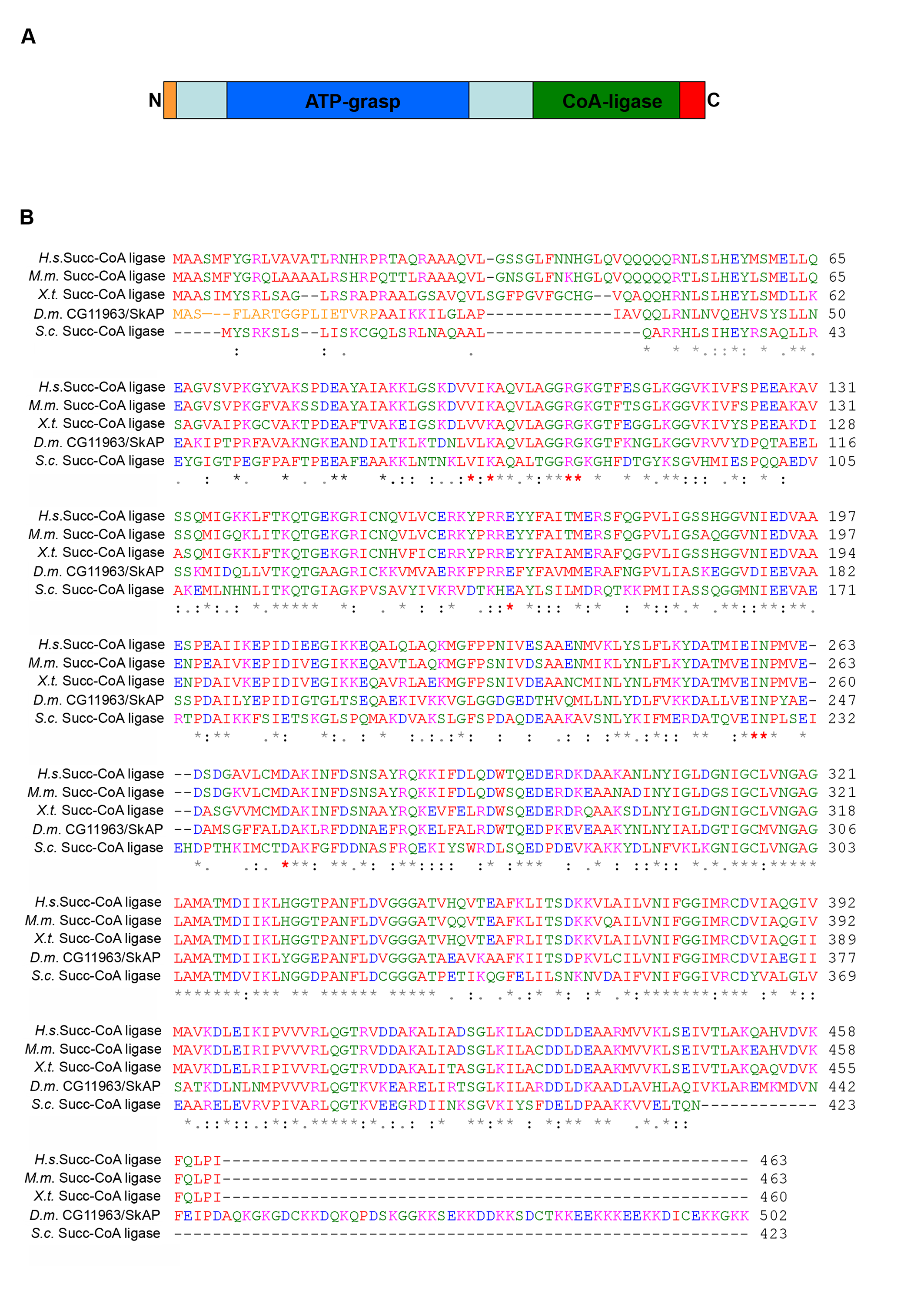

Supplement: Figure S2 — (A) Diagrammatic representation of CG11963/SkAP, showing a putative mitochondrial targeting sequence at the N-terminus (orange) (mitoprot prediction; http://ihg.gsf.de/ihg/mitoprot.html), and the conserved ATP-grasp (blue) and CoA-ligase domains (green) found in Succinate-CoA ligase family members. Note the presence of a highly charged C-terminal extension in CG11963/SkAP (red). (B) Alignment of CG11963/SkAP protein sequence with Succinate CoA ligase family members using ClustalW. Black stars indicate conserved residues. Orange residues indicate predicted mitochondrial targeting sequence. Red stars indicate residues conserved from mammals through to Escherichia coli within the nucleotide binding domain [44]. (2.27 MB TIF) [file pbio.0060098.sg002.tif]

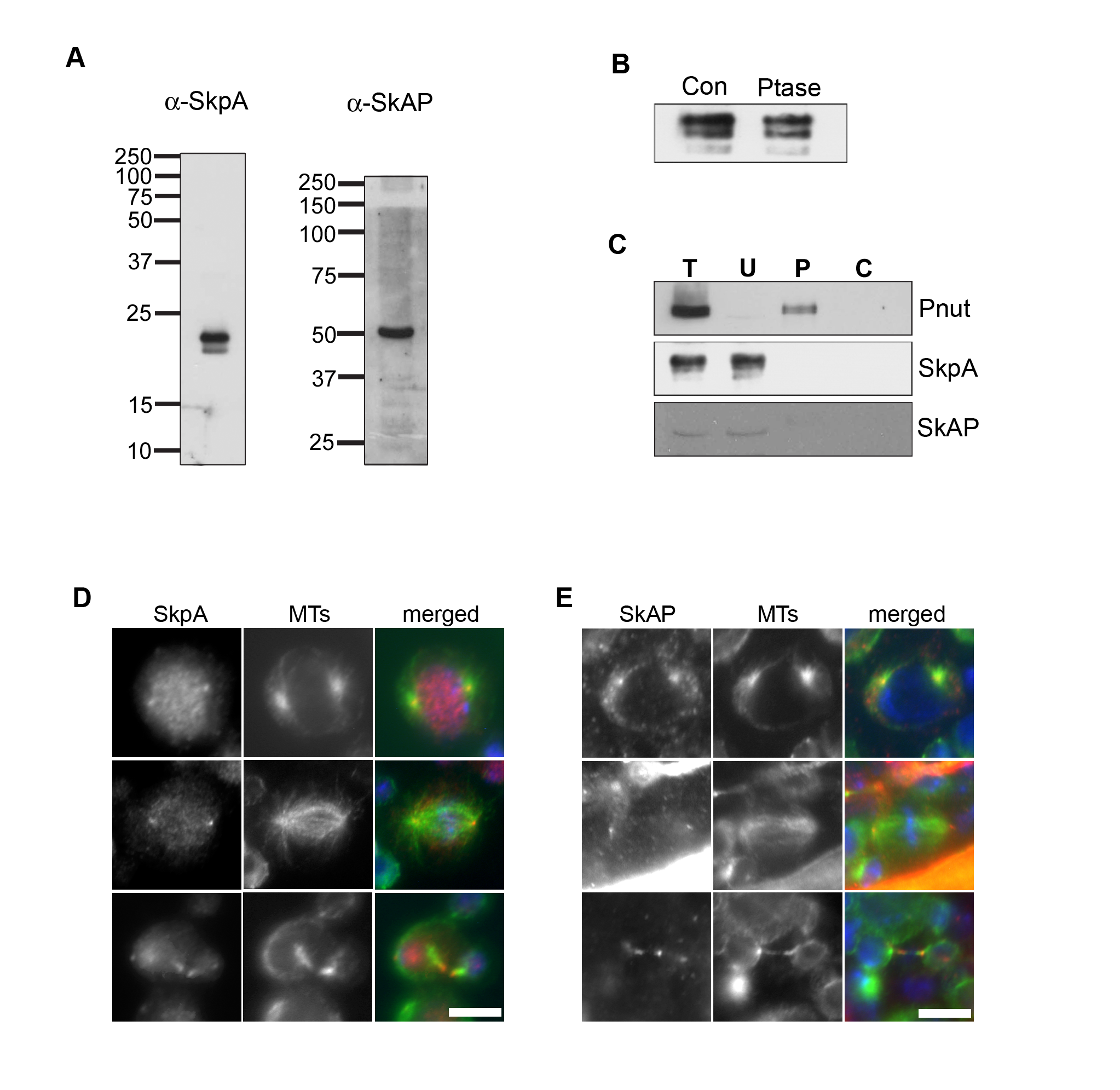

Supplement: Figure S3 — (A) Full-length Western blots of Drosophila embryo extracts probed with affinity-purified rabbit anti-SkpA and anti-SkAP antibodies. The antibodies recognise bands of the predicted molecular weight. In addition, the SkpA antibody recognises a band of slightly lower molecular weight and at lower intensity. (B) The 0–4-h embryo extract treated with λ phosphatase buffer (Con) or phosphatase buffer in the presence of λ phosphatase (Ptase) for 30 min at 37 °C, prior to Western blot analysis using the anti-SkpA antibody. The bands recognised by the antibodies do not resolve, suggesting they are not differentially phosphorylated forms of SkpA. (C) A control immunoprecipitation to show the specificity of the anti-SkpA immunoprecipitation described in Figure 5. Immobilised anti-Pnut antibodies were used to precipitate Pnut from 0–4-h embryo extracts. Neither SkAP nor SkpA coprecipitate with Pnut. C, control precipitate; P, bound precipitate; T, total embryo extract; U, unbound supernatant. (D and E) Localisation of SkpA (D) and SkAP (E) in larval neuroblasts. Cells were fixed according to [53] and stained to visualise DNA (blue), MTs (green), and either SkpA or SkAP (red). Both proteins localise to centrosomes throughout the cell cycle. Scale bar indicates 10 μm. (1.41 MB TIF) [file pbio.0060098.sg003.tif]
