# Supplementary material for: A Microtubule Interactome: Complexes with Roles in Cell Cycle and Mitosis
Source: PLoS Biol. 2008 Apr 22;6(4):e98. doi: 10.1371/journal.pbio.0060098 (PMC2323305; doi:10.1371/journal.pbio.0060098)
Supplement: Table S1 — The table includes all 270 proteins identified in the MT cosedimentation assay, listing the CG number, synonyms, SWISS-PROT–calculated molecular weight(s), the experiment in which it was identified (i.e., 1D or 2D analysis), and the peptide sequences and scores associated with the positive identification. Proteins identified with a score of greater than 30 were considered significant, whereas all lower-scoring proteins were either included or discarded after inspection of individual spectra. This resulted in the inclusion of seven additional proteins with scores of between 26.87 and 29.36. Each individual hit was been assigned a number and grouped into functional classifications, by GO, for ease of cross reference (Figure 2; Table S2). In a small number of cases, a peptide, or set of peptides, matched to more than one possible protein. In the table, these proteins have been assigned a shared number, but are differentiated by a letter. Therefore, although 270 potential MAPs were identified, these are numbered from 1–257. Where duplicated peptide sequences span more than one functional grouping, a star is shown next to the number. (620 KB DOC) [file pbio.0060098.st001.doc]

Supplementary Table S1. **List of 270 putative MAPs, including mass spectrometry peptide sequence identification.** The table includes all 270 proteins identified in the MT co-sedimentation assay, listing the CG number, synonyms, molecular weight as recorded in the mass spectrometry (mascot) database, the experiment in which it was identified (i.e. 1D or 2D analysis) and the peptide sequences associated with the positive identification. Proteins identified with a score of >30 were considered significant, whereas all lower-scoring proteins were either included or discarded after inspection of individual spectra. This resulted in the inclusion of 7 additional proteins with scores of between 26.87 and 29.36. Each individual hit was been assigned a number and grouped into functional classifications, by gene ontology, for ease of cross reference (Figure 2; Supplementary Table S2). In a small number of cases, a peptide, or set of peptides, matched to more than one possible protein. In the Table, these proteins have been assigned a shared number, but are differentiated by a letter. Therefore, although 270 potential MAPs were identified, these are numbered from 1-257. Where duplicated peptide sequences span more than one functional grouping, a star is shown next to the number.

| Number | ORF | Synonyms | Mw | 2D/1D | Peptides | Score |  |
| --- | --- | --- | --- | --- | --- | --- | --- |
| MITOSIS/CELL CYCLE | |  |  |  |  |  |  |
| 1 | CG1404 | Ran | 24708 | 2D | 3 peptides matched NLQYYDISAK (0) KKNLQYYDISAK (2) FNVWDTAGQEK (0) | 89.89 |  |
| 2 | CG16983 | SkpA | 18596 | 2D | 2 peptides matched DFSPAEEEQVR (0) DFSPAEEEQVRKENEWCEEK (2) | 56.24 |  |
| 3 | CG17498 | Mad2 | 23404 | 2D | 3 peptides matched IQNEIRDVMR (1) + 1 Oxidation (M) GIYPAEDFNNTQQYGLTILMSKDPK (1) + 1 Oxidation (M) SFSTGLHKVDTVVNYKMST (2) + 1 Oxidation (M) | 58.62 |  |
| 4 | CG31196 | 14-3-3-ε | 29172 | Both | 1D: 4 peptides matched VASMDVELTVEER (0) + 1 Oxidation (M) NLLSVAYK (0) AAFDDAIAELDTLSEESYK (0) AAFDDAIAELDTLSEESYKDSTLIMQLLR (1) + 1 Oxidation (M)  2D: 5 peptides matched DAAENSLIAYK (0) DICSDILNVLEK (0) YLAEFATGSDRKDAAENSLIAYK (2) LAEQAERYDEMVEAMKK (2) + 2 Oxidation (M) VASMDVELTVEER (0) + 1 Oxidation (M) | 1D: 122.47  2D: 161.37 | |
| 5 | CG17870 | 14-3-3ζ | 28227 | 1D | 1 peptides matched SVTETGVELSNEER (0) | 31.65 |  |
| 6 | CG8705 | Peanut | 60143 | 2D | 8 peptides matched SRVPFAVVGANTIIEQDGKK (2) IYDFPATLEDAAEEAK (0) YEEYLTAESR (0) SRVPFAVVGANTIIEQDGK (1) IKIYDFPATLEDAAEEAK (1) LSELGLVDGK (0) THLQDLKDVTNNVHYENYR (1) QKPMEIAGYVGFANLPNQVYRK (1) + 1 Oxidation (M) | 302.04 |  |
| 7 | CG1403 | Septin 1 | 41131 | 2D | 4 peptides matched STLVNSLFLTDLYPER (0) LEASTVEIEER (0) GFSSIETPGYVGFANLPNQVHR (0) EAVPFAVCGANTLLEVK (0) | 177.72 |  |
| 8 | CG4173 | Septin 2 | 48474 | 2D | 2Da: 1 peptides matched STLMDTLFNTSFESTPSPHTLPSVK (0) + 1 Oxidation (M)  2Db: 1 peptides matched VNIIPVIAK (0) | 2Da:23.98  2Db: 39.76 | |
| 9 | CG2916 | Septin 5 | 48473 | 2D | 2 peptides matched VNIIPVIAK (0) STLMDTLFNTSFGSTPSPHNLPNVK (0) + 1 Oxidation (M) | 40.76 |  |
| 10 | CG3265 | EB1 | 43654, 32488 | Both | 1D: 5 peptides matched TNLEHEYIQNFK (0) GPSATTRPAMTSAVKPTVSK (0) + 1 Oxidation (M) EGAPMGFGSGAVK (0) + 1 Oxidation (M) KNDVSNSVNNQQIEEMSNQVMDMR (1) + 3 Oxidation (M) INLEGLEKERDFYFSK (2)  2D: 9 peptides matched FFDANYDGR (0) EGAPMGFGSGAVKSLPGTAASGVSSSYR (1) + 1 Oxidation (M) SLPGTAASGVSSSYRR (1) Multi-Hit TNLEHEYIQNFKILQAGFKK (2) LRDIEILCQEADDAEAHPIIQK (1) IEELCTGAAYCQFMDMLFPNSVPVKR (1) + 2 Oxidation (M) MSVDKIIPVDKLIK (2) + 1 Oxidation (M) TNLEHEYIQNFK (0) Multi-Hit GPSATTRPAMTSAVKPTVSK (0 | 1D: 155.2  2D: 259.57 | |
| 11 | CG5363 | cdc2 | 29570 | Both | 1D: 1 peptides matched NLDANGIDLIQK (0)  2D: 5 peptides matched IGEGTYGVVYKGR (1) NLDANGIDLIQK (0) IRLESDDEGVPSTAIR (1) VADFGLGR (0) DLKPQNLLIDK (0) | 1D: 122.47  2D: 232 | |
| 12 | CG4488 | Wee 1 | 68808 | 2D | 6 peptides matched NEILMNKLR (1) Multi-Hit SKLQLGLELTVEK (1) ALNEVWAHAVLGK (0) LSSGEEQQLDSK (0) DGKVPILPSLSRDFNELIAQMMHPYPDK (2) + 1 Oxidation (M) MAFRQSEHEMSVTSLDSSVELR (1) + 2 Oxidation (M) | 28.66 |  |
| 13 | CG10498 | cdc2c | 35888 | 2D | 4 peptides matched SLFPGDSEIDQLYR (0) NVQHVDHVALPVDPNAGSASR (0) DLKPQNLLVDTAGK (0) ISAKDALQHAYFR (1) | 123.3 |  |
| 14 | CG6235 | twins/aar | 56966 | 2D | 7 peptides matched SFFSEIISSISDVK (0) NPVHFLLSTNDK (0) DSSIMTGSYNNFFR (0) + 1 Oxidation (M) QIPLLVEASPR (0) QFEEPENPTNR (0) DVTLEASRDIIKPK (1) EENGLIRDPQNVTALR (1) | 264.71 |  |
| 15 | CG7425 | meiotic 68 / UbcD1 | 16678 | 2D | 1 peptides matched SQWSPALTISK (0) | 39.22 |  |
| 16 | CG7581 | Bub3 | 37415 | 2D | 8 peptides matched EGYVMSSIEGR (0) + 1 Oxidation (M) FYDVPANQLR (0) VYSMSVIDEK (0) + 1 Oxidation (M) LNNPPEDLISAVK (0) SNQYMAASSWDGTLR (0) LFPNKEGYVMSSIEGR (1) + 1 Oxidation (M) VAVEYLDHDPEVQR (0) LFDVNTQAESIIGAHEEPIR (0) | 269.27 |  |
| 17 | CG9193 | mus 209 | 28830 | 1D | 1 peptides matched FSASGDVGTANIK (0) | 29.36 |  |
| 18 | CG9765 | D-TACC | 132522 | 2D | 7 peptides matched LNGVIEAYEK (0) SNEESLLAER (0) ICEELIYGKGQGGSS (1) ICEELIYGK (0) AIAELISEKEQQAQLHER (1) EGELREEALIKR (2) TFDNSNTNTEDKTHNYNDMDELEKK (2) + 1 Oxidation (M) | 319.26 |  |
| 19 | CG4254 | twinstar | 17153 | 1D | 1 peptides matched YIQATDLSEASR (0) 8.10% sequence coverage | 33.94 |  |
| 20 | CG10212 | SMC2 | 112750 | 1D | 3 peptides matched SLVALSLVLAMLK (0) + 1 Oxidation (M) SVTLEGDVVDPHGTVSGGAAPK (0) AATEVNTNFSGIFSSLLPGAEAK (0) | 84.21 |  |
| 21 | CG5000 | mini spindles | 226933 | 1D | 11 peptides matched IVSACVAATTLALR (0) LAAVEQLLGEISGFDAK (0) VDIAPQITEALLK (0) SIIEFGFQLQPK (0) GLEAALIFVENSGLAGR (0) NLFPGFLHALGDNK (0) NGAAAADVLSAFAEATKLEYVVGK (1) MVVDSNALAQEKGLEAALIFVENSGLAGR (1) + 1 Oxidation (M) VQSEAFNWVNR (0) LAAVEQLLGEISGFDAKQAGISQILIR (1) IVSACVAATTLALREFGHK (1) | 33.25 |  |
| 22 | CG18242 CG1915 | D-titin | 54075.2065784 | 2D | 14 peptides matched IQVVQEK (0) LIEQDIPK (0) KVKKPTGTVEK (2) CDVQNNVWMKVSDFNK (1) VLKSKVSEEKPK (2) SEEVQEEAKIVK (1) KIQGPNMEVTK (1) VTPSPQKNQYK (1) LKVEWYWNGRPLKAGSR (2) LTVAVKEFIPEKPEEKPFEIVVLEETVESK (1) EEEAPENVTLKNIPQKPQEVEEEVELKQKPK (2) VSEEKPKSK (1) VTGTRPLDVYWLKNGMKIQPSIK (2) FKMTSEFGFVTMDMIEVYAR (1) + 1 Oxidation (M) | 37.91 |  |
| 23 | CG2210 | abnormal wing discs | 17038, 19208, 17169 | 2D | 2 peptides matched QMLGATNPADSLPGTIR (0) + 1 Oxidation (M) TFIMVKPDGVQR (0) + 1 Oxidation (M) | 92.81 |  |
| 24 | CG3210 | dynamin related protein 1 (Drp1) | 82566 | Both | 1D: 2 peptides matched NIETTELCGGAR (0) IVQHCGNEVQQEMLR (0) + 1 Oxidation (M)  2D: 7 peptides matched NIETTELCGGAR (0) SDKAETLLNESDHIAVR (1) TDSDPYSQINLGQR (0) MGYIFHETFGR (0) + 1 Oxidation (M) HIDDQMKDEAAFLQR (1) + 1 Oxidation (M) SSVIESVVGR (0) DIMDQKHIDDQMKDEAAFLQR (2) + 2 Oxidation (M) | 1D: 62.52  2D: 317.52 | |
| 25 | CG17566 | γ-tubulin at 37C | 51296 | 2D | 13 peptides matched CADSVVVLDNTALNR (0) ESMFQDDLTELDIAR (0) + 1 Oxidation (M) DVFFYQADDNHYIPR (0) VINNIMTSPYSK (0) + 1 Oxidation (M) ALAQYDKLR (1) LTKCADSVVVLDNTALNR (1) LYNQENVFLSK (0) AVLIDLEPR (0) VSGLMMANHTGISSLFKR (1) + 2 Oxidation (M) LCLEHGISPDGVLEDFATDGQDRK (1) AVLIDLEPRVINNIMTSPYSK (1) + 1 Oxidation (M) VQEEVFDILDR (0) QCFVSILNIIQGEVDPSQVHK (0) | 489.47 |  |
| CYTOSKELETON BINDING | | |  |  |  |  |  |
| 26 | CG5020 | CLIP-190 | 89324, 189064 | Both | 1D: 19 peptides matched IAEQLEEEQR (0) SQFSMQDLLR (0) + 1 Oxidation (M) LTTYPLAGAQTPTSPLAK (0) YGVFVPIAK (0) IVELESALGNER (0) SHNEIQDKLEQAQQKER (2) SSTPVKPILATPK (0) Multi-Hit IAEQLEEEQRLR (1) SNIPTPATSGTGIPQPSK (0) ELALLKEENEKQAQEAQAEFTR (2) DLDREDAQNQALQLQK (1) VQTLETLPMDFTKPHAFDALTK (0) + 1 Oxidation (M) Multi-Hit MASEDAQKIADLK (1) + 1 Oxidation (M) Multi-Hit LNESNVQLENK (0) SFEESIKNLQEEVTK (1) SGKGDEVESLDIETSLAK (1) VLEEKLQAATSQLDAQQATNKELQELLVK (2) ATSDSLESERVNKSDECEILQTEVR (2) LVSATPSLQSILPPDLPSDDGALQEEIAQLQEK (0)  2D: 6 peptides matched IVELESALGNER (0) YGVFVPIAK (0) SQFSMQDLLR (0) + 1 Oxidation (M) SNIPTPATSGTGIPQPSK (0) SSTPVKPILATPK (0) SNIPTPATSGTGIPQPSKMKAPSSFGSTGSVSK (2) + 1 Oxidation (M) | 1D: 453.87  2D: 180.4 | |
| 27 | CG6384 | CP190 | 121679 | 1D | 3 peptides matched VDNWGVFFLQK (0) IMPNTPAAPTEK (0) + 1 Oxidation (M) SLLYTFAECALCNQSYR (0) | 29.2 |  |
| 28 | CG5981 | stathmin | 29581 | 2D | 3 peptides matched AQAQDLDGQQSAIASSG (0) GGLSYEVILAEPAPNVAVPK (0) VNNTVDTEATEIRCQEK (1) | 54.42 |  |
| 29a | CG4027 | Actin 5C | 41821 | Both | 1D: 4 peptides matched SYELPDGQVITIGNER (0) KDLYANTVLSGGTTMYPGIADR (1) + 1 Oxidation (M) HQGVMVGMGQKDSYVGDEAQSKR (2) + 2 Oxidation (M) EITALAPSTMK (0) + 1 Oxidation (M)  2D: 9 peptides matched SYELPDGQVITIGNER (0) KDLYANTVLSGGTTMYPGIADR (1) + 1 Oxidation (M) QEYDESGPSIVHR (0) HQGVMVGMGQKDSYVGDEAQSKR (2) + 1 Oxidation (M) Multi-Hit AVFPSIVGRPR (0) DLYANTVLSGGTTMYPGIADR (0) + 1 Oxidation (M) VAPEEHPVLLTEAPLNPK (0) SYELPDGQVITIGNERFR (1) TTGIVLDSGDGVSHTVPIYEGYALPHAILR (0) | 1D: 80.19  2D: 386.32 | |
| 29b | CG12051 | Actin 42A | 41823 | 1D | 4 peptides matched SYELPDGQVITIGNER (0) KDLYANTVLSGGTTMYPGIADR (1) + 1 Oxidation (M) HQGVMVGMGQKDSYVGDEAQSKR (2) + 2 Oxidation (M) EITALAPSTMK (0) + 1 Oxidation (M) | 80.19 |  |
| 30 | CG10067 | Actin 57A | 40258 | 1D | 3 peptides matched SYELPDGQVITIGNER (0) HQGVMVGMGQKDSYVGDEAQSKR (2) + 2 Oxidation (M) MCKAGFAGDDAPRAVFPSIVGRPR (2) + 1 Oxidation (M) | 49.1 |  |
| 31 | CG10540 | F-actin capping protein α | 32759 | 2D | 1 peptides matched ESVVVSNEQQVAK (0) | 66.14 |  |
| 32 | CG6174 | Actin-related protein 87C | 42719 | Both | 1D: 1 peptides matched EQLATFTEDHPVLLTEAPLNPR (0)  2D: 2 peptides matched VMAGALEGDIFVGPKAEEHR (1) + 1 Oxidation (M) Multi-Hit IFEIGPAR (0) | 1D: 30.4  2D: 91.29 | |
| 33 | CG54125 | myosin III | 131847 | 2D | 4 peptides matched TGKMSGAVFNMYMLEK (1) + 2 Oxidation (M) QEFPQEFHAK (0) FEIYEEIAQGVNAK (0) RGTCIGSPCWMAPEVVSAMESR (1) + 1 Oxidation (M) Multi-Hit | 36.19 |  |
| 34 | CG5336 | Ced-12 | 82976 | Both | 1D: 2 peptides matched LLQDVNSPVMR (0) + 1 Oxidation (M) MIEDGGQTNEDILK (0) + 1 Oxidation (M)  2D: 3 peptides matched QNAIALLNALFVK (0) LLQDVNSPVMR (0) + 1 Oxidation (M) ATAEDFSTTFSVVREQIQR (1) | 1D: 59.89  2D: 76.24 | |
| 35 | CG6450 | lava lamp | 202847 | 2D | 5 peptides matched QKLAELQTVKHSK (2) Multi-Hit DAELQDANLVSK (0) Multi-Hit QDMDVQMYHAR (0) + 1 Oxidation (M) RERQEADQEVFQLGQK (2) ELPQSQQSTQGEATSDIMQKMQK (1) + 1 Oxidation (M) | 41.92 |  |
| 36 | CG10966 | receptor-degeneration-A | 160142 | 1D | 3 peptides matched NNAASGSGGGGAGGGAGGGGGK (0) RDICVMLVAAGAHLDTLDSGGNTPMMVAFNKNANEIATYLESK (2) + 3 Oxidation (M) Multi-Hit QVFDLTQGGPK (0) | 32.09 |  |
| 37 | CG2331 | TER94 | 88859 | 1D | 5 peptides matched SAAPCVLFFDELDSIAK (0) LAGESESNLR (0) SAAPCVLFFDELDSIAKAR (1) EIDIGIPDATGRLEVLR (1) EKMDLIDLEDDKIDAEVLASLAVTMENFR (2) + 2 Oxidation (M) | 140.52 |  |
| 38 | CG11064 | Rfa-bp | 372676 | Both | 1D: 5 peptides matched GLVNKDFDEIVK (1) ELYIAVGNLVAK (0) IVNHLIENVQLTETYK (0) QSDSETLLELAAFPHPNK (0) VQYQGKALSGNFK (1)  2D: 6 peptides matched TILSDLVDSTGDYVK (0) SLSGNTVAALSECASTGR (0) ELYIAVGNLVAK (0) SVDKETLNQAASQLLPNAPK (1) QSDSETLLELAAFPHPNK (0) SLIFQRPETYTSK (0) | 1D: 226.88  2D: 210.61 | |
| MOTORS | |  |  |  |  |  |  |
| 39 | CG7507 | Dynein heavy chain 64C | 530184 | 1D | 10 peptides matched DVLNLVSEGIGLIWESYK (0) TLLKALER (1) Multi-Hit MVWRVSPAHKK (2) FLSDPQVQALYVQR (0) MYESYEYVK (0) + 1 Oxidation (M) LALESICLLLGENATDWKSIR (1) ETKDLVEQLERSIAAYK (2) FYFVGDEDLLEIIGNSK (0) QTNHHRSMLSELVRGIIPK (2) EELRSLEEQADVNLASAKETK (2) | 29.06 |  |
| 40 | CG18000 | Dynein intermediate chain (short wing) | 71457 | 1D | 1 peptides matched DVAPAITPLEIK (0) | 41.26 |  |
| 41 | CG10751 | Dynein light chain (roadblock) | 10823 | 2D | 3 peptides matched GVVGTIVVNNEGIPVK (0) DLDPSNDMTFLR (0) SVVRDLDPSNDMTFLR (1) + 1 Oxidation (M) | 150.25 |  |
| 42 | CG7765 | Kinesin heavy chain | 110399 | 1D | 2 peptides matched FVSSPEDVFEVIEEGKSNR (1) RITEMLTNLLRDLGEVGQAIAPGESSIDLK (2) | 27.57 |  |
| 43 | CG5433 | Kinesin light chain | 58044 | 2D | 9 peptides matched  YTEAEILYK (0)  EAANLLNDALSIR (0)  HNMSPTPPSQFANQTSGYEIPAR (0) + 1 Oxidation (M)  TLGENHPAVAATLNNLAVLYGK (0)  QGMFEAAETLEDCAMR (0) + 2 Oxidation (M)  ALDIYESKLGPDDPNVAK (1)  YTEAEILYKQVLTR (1)  AIKEDLDFSEEKNAKP (2)  TDPVVELFPDEENEDRHNMSPTPPSQFANQTSGYEIPAR (1) + 1 Oxidation (M) | 278.21 | |
| 44 | CG8590 | Klp3A | 135812 | 1D | 32 peptides matched GLLALGNVINALGSGQAAGYIPYR (0) Multi-Hit IIMPGLTELVVTSAQQVTDHLIR (0) + 1 Oxidation (M) Multi-Hit ILASLEEELEMR (0) + 1 Oxidation (M) LQLLEQEISDLR (0) EGVNINKGLLALGNVINALGSGQAAGYIPYR (1) LLQDSLGGNSITLMIACVSPADYNVAETLSTLR (0) + 1 Oxidation (M) FNLVDLAGSER (0) AQLLDAQQQEDAASKR (1) TTLDELRAQLLDAQQQEDAASKR (2) Multi-Hit TSDPAEAAEQAR (0) AVAATAMNETSSR (0) IQNLSTEIR (0) AVINNHYHLLQQEKTSDPAEAAEQAR (1) NQILEELLSSR (0) Multi-Hit SHAIFTLTLVATKLDGK (1) AHIAEQAHDKLR (1) AYEEKVSVLIR (1) AVHDIFTAIAEMQSEFR (0) + 1 Oxidation (M) Multi-Hit EALQQELDKLR (1) LLQSQHEEQMLAQQR (0) + 1 Oxidation (M) ILASLEEELEMRNAQISDLQQKVCPTDLDSR (2) + 1 Oxidation (M) TTLDELRAQLLDAQQQEDAASK (1) DLMDQLR (0) + 1 Oxidation (M) MSSSLISAVGAAGLGAIPCEESLAGSMANAAEIQR (0) + 2 Oxidation (M) EKIQNLSTEIR (1) VRPLVQSELDRGCR (1) VRPLVQSELDR (0) KLQQELHQSLLDLTEKEMR (2) + 1 Oxidation (M) Multi-Hit ILASLEEELEMRNAQISDLQQK (1) + 1 Oxidation (M) NKPVVNLDPHAAEVNMLKDVIQK (1) + 1 Oxidation (M) SLAEGVQSLGESR (0) IIMPGLTELVVTSAQQVTDHLIRGSAGR (1) + 1 Oxidation (M) | 1254.19 |  |
| 45 | CG6392 | CENP-meta | 257994 | 2D | 6 peptides matched ESSMSESLLR (0) QSDATEPICLK (0) Multi-Hit TSITELQSQVSDLNAELENHLR (0) Multi-Hit VLKDKLAEEER (2) ENQAKFYAELQETKDR (2) ANQMKLDQSEPGLK (1) + 1 Oxidation (M) | 38.33 |  |
| 46 | CG1453 | Klp10A | 88671 | Both | 1D: 20 peptides matched NPTQSAIGGNLTSR (0) FSFIDLAGNER (0) TIFEGGMATCFAYGQTGSGK (0) + 1 Oxidation (M) KDMLIVHEPR (1) + 1 Oxidation (M) VVDGVEEVLK (0) MEGAEINKSLLALKECIR (2) + 1 Oxidation (M) TCMIAMISPGLSSCEHTLNTLR (0) + 2 Oxidation (M) GKEVELDAILTLNPELMQDTVEQHAAPEPK (1) + 1 Oxidation (M) EIDVISVPR (0) KQATAPMNLSRNPTQSAIGGNLTSR (2) + 1 Oxidation (M) SHAVFQIVLRPQGSTK (0) TDGRVHMAVVAVINQSGK (1) + 1 Oxidation (M) VHMAVVAVINQSGK (0) + 1 Oxidation (M) IASAVPNNTLPNPSAAASAGPAAQGVATAATTQGAGGASTR (0) VLEDGKQQVQVVGLTEKVVDGVEEVLK (2) ALYNLTNYVDYDQDSYCKR (1) EIDVISVPRKDMLIVHEPR (2) + 1 Oxidation (M) GESMFSQLLDIAIQCR (0) + 1 Oxidation (M) EVELDAILTLNPELMQDTVEQHAAPEPK (0) + 1 Oxidation (M) IASAVPNNTLPNPSAAASAGPAAQGVATAATTQGAGGASTRR (1)  2D: 5 peptides matched TCMIAMISPGLSSCEHTLNTLR (0) + 2 Oxidation (M) SHAVFQIVLRPQGSTK (0) KQATAPMNLSRNPTQSAIGGNLTSR (2) + 1 Oxidation (M) NPTQSAIGGNLTSR (0) TIFEGGMATCFAYGQTGSGK (0) + 1 Oxidation (M) | 1D: 559.4  2D: 163.46 | |
| 47 | CG9191 | Klp61F | 121163 | 1D | 9 peptides matched SNQNIQVYVR (0) SAEVVDVVGPR (0) SSWEDDSDIGIIPR (0) ETVNINQSLLTLGR (0) KLEQLGAMSLPDAEELQNLQEELANER (1) + 1 Oxidation (M) NLNQKYEKETNENVGSVR (2) ALAQQEDALLESMMMQMEQIKNLR (1) + 4 Oxidation (M) EIQTNLQVIEENNQR (0) ISYLELYNEELCDLLSTDDTTKIR (1) | 282.49 |  |
| 48 | CG10923 | Klp67A | 92350 | 1D | 4 peptides matched MAEMNPLAVPVALR (0) + 2 Oxidation (M) LVSDLISDQNVR (0) LSMIDLAGSER (0) + 1 Oxidation (M) FANYMSTLTSQMEK (0) + 2 Oxidation (M) | 109.21 |  |
| 49 | CG12298 | subito / KIF20A | 71386 | 1D | 5 peptides matched GLTSVFVTSSEEALR (0) NINTSLMVLGR (0) + 1 Oxidation (M) LAMIVTVTPLDKYYEENLNVLNFASIAK (1) + 1 Oxidation (M) VSYCGFMEFSK (0) + 1 Oxidation (M) MSTCEGGDYTKELEDENVR (1) + 1 Oxidation (M) | 109.32 |  |
| VESICLE-MEDIATED TRANSPORT | | |  |  |  |  |  |
| 50 | CG9543 | ε COP | 34619 | 2D | 5 peptides matched QQNEEDTNSVLFDAR (0) EGNSTPLQALR (0) IVASDIKEGNSTPLQALR (1) LVHEAFEQPSR (0) LVHEAFEQPSRTEELLEK (1) | 120.91 |  |
| 51 | CG8014 | Receptor mediated endocytosis 8 | 272547 | 2D | 3 peptides matched ILDILIK (0) IGDYYIRLILEKDDWPQNLVK (2) DGQKPERQGPITYSELK (1) | 37.09 |  |
| 52 | CG4422 | GDP dissociation inhibitor | 49900 | 2D | 2 peptides matched DGLSTQIIIPQK (0) KSDIYVSLVSSTHQVAAK (1) | 63.59 |  |
| 53 | CG8309 | Tango7 | 44086 | 2D | 3 peptides matched QCEQVLEVFSGVDQLK (0) EGLTSVSSAQLDLAR (0) IIGVCDVCFK (0) | 178.93 |  |
| 54 | CG10686 | trailer hitch | 69339 | 1D | 1 peptides matched GGGSGTDFYNQQR (0) | 44.98 |  |
| 55 | CG8983 | ERp60 | 55370 | 2D | 4 peptides matched MDATANDVPPEFNVR (0) + 1 Oxidation (M) YSVSGYPTLK (0) AAEIVKDDDPPIKLAK (2) TQDSVKDFQNPLITAYYSVDYQKNPK (2) | 153.95 |  |
| 56 | CG9057 | Lipid storage droplet-2 | 38229 | Both | 1D: 7 peptides matched GNINDYLSSLIAALK (0) SSENDMPVPASEDPVLHTVQTVGR (0) + 1 Oxidation (M) SKVIDVVQPHLER (1) LDRPIAYVDQTLVK (0) AVTTAAPFVTK (0) DTPQEIYNQAK (0) DLLPHLESLER (0)  2D: 9 peptides matched AASLKDLAWQKANEVLATQYGSLAVNGVDTTTALAER (2) ANEVLATQYGSLAVNGVDTTTALAER (0) VFEWALTAAEDCVTR (0) SKVIDVVQPHLER (1) AVTTAAPFVTK (0) DTPQEIYNQAK (0) APIIKDTPQEIYNQAK (1) AVTTAAPFVTKLDRPIAYVDQTLVK (1) Multi-Hit IIKLPVVNAAWDKSQDVYGK (2) | 1D: 286.71  2D: 380.79 |  |
| 57 | CG5474 | Signal sequence Receptor β | 21206 | 2D | 2 peptides matched YTIFNVGSGAATK (0) LVDSGFHPEAFDVVGGQPTAVVDR (0) | 125.92 |  |
|  |  |  |  |  |  |  |  |
| POLARITY | |  |  |  |  |  |  |
| 58 | CG12047 | Mud | 286527 | 2D | 7 peptides matched ETVDRLLTREYLSQAIANVAVVYR (2) EHENAELKEK (1) ECDVQSVCVEK (0) NINLENMLSQIADK (0) + 1 Oxidation (M) LSNQIVRLNEK (1) DAAKSASELEALTAQNAK (1) LTESQAKLEMQVAELQVELENK (1) Multi-Hit | 33.03 |  |
| 59 | CG10545 | Gβ13F | 37132 | 2D | 7 peptides matched KAACDTSLLQAATSLEPIGR (1) TFVSGACDASAK (0) AACDTSLLQAATSLEPIGR (0) Multi-Hit ADQELAMYSHDNIICGITSVAFSK (0) + 1 Oxidation (M) LIVWDSHTTNK (0) ELPGHGGYLSCCR (0) Multi-Hit IYAMHWGNDSR (0) | 337.11 |  |
| 60 | CG12021 | PatJ | 92851 | 2D | 2 peptides matched FEQLQQAIAANDK (0) TILPGGVADKDGR (1) | 30.51 |  |
| DNA REPLICATION / REPAIR | | |  |  |  |  |  |
| 61 | CG6258 | RfC 38kD subunit | 40815 | 1D | 9 peptides matched APFTANQEIPDLDWQVFLR (0) TVVIDLIK (0) ALLMLEAAK (0) + 1 Oxidation (M) SETMTFTTPSNR (0) + 1 Oxidation (M) VAAPNETEIVSILQNTCKR (1) VIVISEGDELTK (0) ETASQIISEQTPAKLEK (1) LYELLTQGVPPNLIFR (0) FLSELDMTDDF (0) + 1 Oxidation (M) | 212.28 |  |
| 62 | CG14999 | RfC 40kD subunit | 37173 | Both | 1D: 4 peptides matched IIDGINSLLQLTALLAK (0) LSVFATQGNAPNIIIAGPPGVGK (0) IIEPIQSR (0) EAVLELNASNER (0)  2D: 4 peptides matched FKEIVGNEDTVAR (1) TTTIQCLAR (0) EIVGNEDTVAR (0) EAVLELNASNER (0) | 1D: 213.96  2D: 181.21 | |
| 63 | CG5313 | RfC 3 | 37407 | Both | 1D: 8 peptides matched SMVLELNASDDR (0) + 1 Oxidation (M) ALLSGSSLEDSFK (0) GQILNFASTR (0) IIEAEAVQITEDGKR (1) LIILDEADAMTNDAQNALR (0) + 1 Oxidation (M) YRPSGLDDLISHEEIISTITR (0) FAPLSQDQMMPR (0) + 2 Oxidation (M) LIILDEADAMTNDAQNALRR (1) + 1 Oxidation (M)  2D:13 peptides matched IIEAEAVQITEDGKR (1) Multi-Hit ALLSGSSLEDSFK (0) GQILNFASTR (0) SMVLELNASDDRGIGIVR (1) + 1 Oxidation (M) QLPHLLFYGPPGTGK (0) LEKIIEAEAVQITEDGKR (2) FCVICNYLSK (0) LIILDEADAMTNDAQNALR (0) + 1 Oxidation (M) FAPLSQDQMMPR (0) + 2 Oxidation (M) Multi-Hit TIFCDTFK (0) FRFAPLSQDQMMPR (1) + 2 Oxidation (M) LIILDEADAMTNDAQNALRR (1) + 1 Oxidation (M) ALLSGSSLEDSFKTVESAK (1) | 1D: 363.01  2D: 567.02 | |
| 64 | CG1119 | RfC 140 | 111349 | 1D | 4 peptides matched DKLNDVPAVTLK (1) SLVNYCYDLR (0) LDYAPFLLDNIVRPLAK (0) ISPAKVEEIIAATNNDIR (1) | 75.44 |  |
| 65 | CG9633 | Replication protein A70 | 66624 | Both | 1D: 2 peptides matched IGEPVTYENAAK (0) YNLVPISDVSGMENK (0) + 1 Oxidation (M)  2D: 7 peptides matched VLIISELTVVNPGAEVK (0) LFSMDLMDESGEIR (0) + 2 Oxidation (M) AAVDTIGICKEVGELQSFVAR (1) AEQIFSALNFTSHIFK (0) IGEPVTYENAAK (0) IMHGEVVDAPVLQILAIK (0) + 1 Oxidation (M) YNLVPISDVSGMENK (0) + 1 Oxidation (M) | 1D: 29.94  2D: 265.05 | |
| 66 | CG4978 | Mcm7 | 81284 | 2D | 2 peptides matched VSILAAANPAFGR (0) MAQPGDHIVVSGVFLPLMR (0) + 2 Oxidation (M) | 36.79 |  |
| 67 | CG5949 | DNA polymerase δ | 124905 | 1D | 1 peptides matched VTGVPLESLLTR (0) | 34.26 |  |
| 68 | CG6146 | Topoisomerase 1 | 111903 | 1D | 5 peptides matched HKSSSKDK (2) Multi-Hit MKKEPEPAVSPGK (2) + 1 Oxidation (M) HKSSSGHHK (1) Multi-Hit EREHKSSNSSSSSK (2) SSSSHKSSSSSSSSK (1) | 35.36 |  |
| 69 | CG4003 | Pontin | 50159 | 2D | 13 peptides matched TISNVVIGLK (0) AQTEGLQLEENAFTR (0) SLESPIAPIVIFATNR (0) GLGLDEVGAAVHSAAGLVGQK (0) Multi-Hit EAAGIVVDLIK (0) QLKLDPSIFDALQK (1) NQISKDDIEDVHSLFLDAKR (2) GTTDIVSPHGIPLDLLDR (0) EAAGIVVDLIKSKK (2) TLLYSTADMEQIIK (0) + 1 Oxidation (M) YAVQLLTPAHQMCK (0) + 1 Oxidation (M) IRETKEVYEGEVTELTPVETENPMGGYGK (2) + 1 Oxidation (M) EVYEGEVTELTPVETENPMGGYGK (0) + 1 Oxidation (M) | 583.74 |  |
| 70 | CG9750 | Reptin | 53541 | 2D | 5 peptides matched GLGLDDVLEAR (0) VYSLFLDENR (0) ALESDMAPVVVMATNR (0) + 2 Oxidation (M) FVQCPEGELQK (0) ILKEYQDDYMFSEITEEVERDPAAGGGAK (2) + 1 Oxidation (M) | 246.62 |  |
| 71 | CG2905 | Nipped|Tra1 | 436617 | 2D | 11 peptides matched AGNETVRETHQTALIGMLVASATK (1) + 1 Oxidation (M) Multi-Hit QALDVLTPAMPLR (0) Multi-Hit KELLIAARHIFATDLR (2) LSEVQAR (0) HETGVSFRAVIK (1) EKIINIIFKVMESDK (2) + 1 Oxidation (M) LIEGSEDRYSDGNCMEHSVNIVNSAVDIIMTRFNK (2) + 1 Oxidation (M) SFDIIRETTVQGK (1) GLLLAQIGR (0) NLMIEASSPYR (0) + 1 Oxidation (M) QHVKTIITMMLK (1) | 33.42 |  |
| 72 | CG8169 | pms2 | 101283 | 2D | 4 peptides matched GEALSSLCALSDMVIQTRHK (1) Multi-Hit RSIFEFKTDAMALDK (2) AMFASRACRK (2) YHTSKIREFVDLLGVETFGFR (2) | 45.49 |  |
|  |  |  |  |  |  |  |  |
| TRANSCRIPTION | |  |  |  |  |  |  |
| 73 | CG17228 | prospero | 153569 | 2D | 5 peptides matched SGSGSGSHSSMASDGSLR (0) + 1 Oxidation (M) MSGTDLEGLADVLK (0) KEEQQQQIQR (1) SGSGSGSHSSMASDGSLRR (1) EFYYIQMEKYAR (1) + 1 Oxidation (M) | 31.29 |  |
| 74 | CG7008 | tudor SN | 103099 | 1D | 4 peptides matched LHADFQSNPPIAGSYTPK (0) EGRPTAEQQTLIELEDQAR (0) LAALPPAFSSEKPYATEYALALVALPTDNEDKEEALR (1) VQGSNATVLYIDYGNKETLPTNR (1) | 58.61 |  |
| 75 | CG3644 | bicaudal | 17738 | 2D | 4 peptides matched TITEMVPGILTQLGPQDINQLKK (1) + 1 Oxidation (M) TITEMVPGILTQLGPQDINQLK (0) + 1 Oxidation (M) Multi-Hit LSVNTIPGIEEVNIIKNDGTVIHFNNPK (1) AQASLPTNTFAITGHGENK (0) | 135.71 |  |
| 76 | CG4204 | Elongin B | 13257 | 2D | 2 peptides matched AQAPAQLGLTFR (0) TTIFTDAKENTTVAELKR (2) | 41.56 |  |
| 77 | CG7885 | RNAP II 33kD subunit | 31243 | 2D | 1 peptides matched IGLIPLISDDVVER (0) | 34.31 |  |
| 78 | CG8651 | trithorax | 364664 | 2D | 5 peptides matched IPIVSIMKK (1) + 1 Oxidation (M) KEEQRTVSQEQEQSK (2) NSSQVQMLK (0) AFGAPMDEDDEGGVTFRR (1) Multi-Hit LLEEGAISTKKPLSLGDSK (1) | 42.58 |  |
| 79 | CG3696 | kismet | 573636 | 2D | 10 peptides matched FNKIPQR (1) MEVDDEVGKSDK (1) Multi-Hit LGGNKAPQLK (1) EALDESTNLNKDK (1) DAATEKAAATEK (1) Multi-Hit MDGNAQNMFHGNHQGDPYYRYDAAAAAAAAAAANPRAMGPPGGYPPR (2) NLILSAGKMVLIDKLLPK (2) + 1 Oxidation (M) KGTTSANIPNLMNTMMELRK (2) + 1 Oxidation (M) EETIIEVELTNIQK (0) FKTEGESKK (2) | 45.64 |  |
|  |  |  |  |  |  |  |  |
| TRANSLATION | |  |  |  |  |  |  |
| 80 | CG9946 | elF2α | 38645 | Both | 1D: 2 peptides matched GLELSTEELPIR (0) INLIAPPLYVMTTSTTK (0) + 1 Oxidation (M)  2D: 8 peptides matched GLELSTEELPIR (0) AEAENAQVAGDDDEEDGADQEGMQFDPEKEFNHK (1) + 1 Oxidation (M) INLIAPPLYVMTTSTTK (0) + 1 Oxidation (M) Multi-Hit QSVTDPTVFDECNLEPETK (0) EVLLSNIK (0) AKTSEYDGEFKVIMAPK (2) + 1 Oxidation (M) VSPEDVEKCTER (1) INLIAPPLYVMTTSTTKK (1) Multi-Hit | 1D: 76.09  2D: 231.12 | |
| 81 | CG4153 | eIF2β | 35217 | 2D | 10 peptides matched LFFLQCESCGSR (0) SPETILQKDTR (1) Multi-Hit ELDELFADQADDDK (0) TSFANFMDIAK (0) + 1 Oxidation (M) Multi-Hit KTSFANFMDIAK (1) + 1 Oxidation (M) VFEIILDKNPDMAAGR (1) + 1 Oxidation (M) FVMRPPQVLR (0) + 1 Oxidation (M) VFEIILDKNPDMAAGRKPK (2) + 1 Oxidation (M) YIKEYVTCHTCR (1) RVFEIILDKNPDMAAGR (2) + 1 Oxidation (M) | 334.02 |  |
| 82 | CG8882 | eIF3-S2 / Trip1 | 36159 | 2D | 5 peptides matched NADSSLSEQEPTLR (0) LFDSESLMCLK (0) + 1 Oxidation (M) SYASGGEDGFVR (0) IWDVEYGTVIASIPTK (0) VVDSGTDHSAGINDMQLSK (0) + 1 Oxidation (M) | 169.63 |  |
| 83 | CG9075 | eIF4A | 45878 | 2D | 8 peptides matched LFVLDEADEMLSR (0) + 1 Oxidation (M) Multi-Hit KGVAINFITDDDRR (2) GFKDQIQDVFK (1) GIYGYGFEKPSAIQQR (0) Multi-Hit ILESGCHVVVGTPGR (0) Multi-Hit CFMRDPVSILVK (1) + 1 Oxidation (M) KVDQLTQEMSIHNFTVSAMHGDMEQR (1) + 3 Oxidation (M) VVMALGEYMKVHSHACIGGTNVR (1) + 2 Oxidation (M) | 324.09 |  |
| 84 | CG4035 | eIF4E | 27827, 29223 | Both | 1D: 2 peptides matched ISIWTADGNNEEAALEIGHK (0) TSAPSTEQGRPEPPTSAAAPAEAK (0)  2D: 4 peptides matched ISIWTADGNNEEAALEIGHK (0) TSAPSTEQGRPEPPTSAAAPAEAK (0) NNSLQYQLHKDTMVK (1) + 1 Oxidation (M) QGSNVKSIYTL (1) | 1D: 89.98  2D: 128.24 | |
| 85 | CG4429 | eIF4H /RNA-bp 2 | 38263, 34904, 35061 | 2D | 1 peptides matched TIAAPINAVAETK (0) | 43.15 |  |
| 86 | CG8280 | eEF1α 48D | 50306, 38797 | Both | 1D: 6 peptides matched IGGIGTVPVGR (0) VETGVLKPGTVVVFAPANITTEVK (0) SGDAAIVNLVPSKPLCVEAFQEFPPLGR (0) ALRLPLQDVYK (1) TLIDALDAILPPARPTDK (0) EGNADGKTLIDALDAILPPARPTDK (1)  2D: 11 peptides matched TLIDALDAILPPARPTDK (0) ALRLPLQDVYK (1) Multi-Hit IGGIGTVPVGR (0) EHALLAFTLGVK (0) YEEIKKEVSSYIK (2) VETGVLKPGTVVVFAPANITTEVK (0) ALRLPLQDVYKIGGIGTVPVGR (2) Multi-Hit EGNADGKTLIDALDAILPPARPTDK (1) TIEKFEKEAQEMGK (2) + 1 Oxidation (M) LPLQDVYKIGGIGTVPVGR (1) KEGNADGKTLIDALDAILPPARPTDK (2) | 1D: 213.49  2D: 432.21 | |
| 87 | CG11901 | eEF1γ | 48967 | 2D | 15 peptides matched KAAEPAEELDAADEALAAEPK (1) AAEPAEELDAADEALAAEPK (0) GTFNFDDFKR (1) QEAEAVLQQLNQK (0) ALIAAQYSGAQVK (0) VKGTLYTYPENFR (1) Multi-Hit NSTAKQEAEAVLQQLNQK (1) LVTQYFSWSGTDKDGR (1) VADNFKFGETNK (1) LQDATFLAGER (0) EAPKKAAEPAEELDAADEALAAEPK (2) VFMSCNLITGMFQR (0) + 2 Oxidation (M) SAFGNVNRWFVTILNQK (1) VADNFKFGETNKSAEFLK (2) VKGTLYTYPENFRAYK (2) | 593.33 |  |
| 88 | CG4912 | eEF1δ | 28934 | 2D | 1 peptides matched ITQDGLLWGASK (0) | 27.12 |  |
| 89 | CG2238 | eEF2B | 94327 | 1D | 2 peptides matched AYLPVNESFGFTADLR (0) ALPMPDGLPEDIDNGDVSAKDEFKAR (2) + 1 Oxidation (M) | 26.87 |  |
| 90 | CG12141 | Lysyl-tRNA synthetase | 64660 | 2D | 4 peptides matched SSAAEEEEISPNEYFKLR (1) YLDLILNNNVR (0) SYKSEADFEIDTSKLR (2) LAMFLTDSNNIKEVLLFPAMKPEDANR (1) + 2 Oxidation (M) | 64.9 |  |
| 91 | CG10506 | Glutaminyl-tRNA synthetase | 87508 | Both | 1D: 2 peptides matched AININFGYAAAHDGVCYLR (0) IGFFSVDPDTSANHLVFNR (0)  2D: 8 peptides matched IGFFSVDPDTSANHLVFNR (0) LVVLEPLKVTIK (1) VDAALEYLLK (0) LFTLTALR (0) RLVVLEPLKVTIK (2) AAIDVEIFDLLGPK (0) AGDDLIAKFQALGMSEQK (1) + 1 Oxidation (M) MKVTLEEGKMDPVAYR (2) + 2 Oxidation (M) | 1D: 28.76  2D: 294.17 | |
| 92 | CG3821 | Aspartyl-tRNA synthetase | 59041 | 2D | 5 peptides matched VVMLYLGLDNIR (0) + 1 Oxidation (M) EAGVETGDEEDLSTPNEKLLGR (1) FLEPPLILQFADGVAMLR (0) + 1 Oxidation (M) Multi-Hit AQLPLQIEDASRPENADDAEGLNIR (0) EIESVGQQYKVDAFKFLEPPLILQFADGVAMLR (2) + 1 Oxidation (M) | 177.6 |  |
| 93 | CG14792 | Stubarista (Ribosomal protein 40 / Laminin Receptor) | 30228 | 2D | 12 peptides matched LLVVTDPNTDHQPIMEASYVNIPVIAFTNTDSPLR (0) + 1 Oxidation (M) RADGVNILNLGK (1) ADGVNILNLGK (0) YIDIAIPCNNK (0) AVLKFAKYTDTTPIAGR (2) Multi-Hit FAKYTDTTPIAGR (1) FTPGAFTNQIQPAFR (0) ADGVNILNLGKTWEKLQLAAR (2) MLVATTHLGSENVNFQMEQYVYKR (1) + 2 Oxidation (M) FTPGAFTNQIQPAFREPR (1) Multi-Hit AIVAIDNPSDIFVISSRPIGQRAVLK (1) YTDTTPIAGRFTPGAFTNQIQPAFREPR (2) | 532.91 |  |
| 94 | CG5119 | PAbp | 69925 | 1D | 4 peptides matched ALDTMNFDLVR (0) + 1 Oxidation (M) IAFSPYGNITSAK (0) NLDDTIDDDRLR (1) NFTEDFDDEKLKEFFEPYGK (2) | 131.02 |  |
| 95 | CG6779 | Ribosomal protein S3 | 35717, 27471 | Both | 1D: 1 peptides matched ELAEDGYSGVEVR (0)  2D: 3 peptides matched ELAEDGYSGVEVR (0) GLCAIAQAESLR (0) FVDGLMIHSGDPCNDYVETATR (0) + 1 Oxidation (M) | 1D: 48.14  2D: 141.85 | |
| 96 | CG7014 | Ribosomal protein S5b | 25434 | 2D | 2 peptides matched VNQAIWLICTGAR (0) TVAECLADELINAAK (0) | 88.32 |  |
| 97 | CG14206 | Ribosomal protein S10b | 17878 | 1D | 1 peptides matched GDVGPGAGEVEFR (0) | 64.29 |  |
| 98 | CG4916 | maternal expression at 31B | 51945 | 1D | 2 peptides matched DPQILLFSATFPLTVK (0) TGAYCIPVLEQIDPTKDYIQALVMVPTR (1) + 1 Oxidation (M) | 59.14 |  |
| 99 | CG6137 | aubergine | 98559 | 1D | 6 peptides matched AVQDSPYVLELVTK (0) GGNDQAIMIIPELAR (0) + 1 Oxidation (M) ETQEFVQMCIR (0) + 1 Oxidation (M) TCVDRPVPSQVVTLK (0) AVGSVQSTDAEQFQVLNLILRR (1) MQLWPGYQTSIR (0) + 1 Oxidation (M) | 225.67 |  |
| 100 | CG11181 | cup | 127338 | 1D | 3 peptides matched ADTQLLLLR (0) EAVPEQQSSQVQQK (0) VMSGFLVVSKPK (0) + 1 Oxidation (M) | 82.32 |  |
| PROTEIN FOLDING | |  |  |  |  |  |  |
| 101 | CG10578 | Hsp-40 / DnaJ-like | 37027 | 2D | 8 peptides matched QALCGALVSVPTLQGSR (0) ITFPQEGDSAPNK (0) INGLGLPVPK (0) DIFDNYGEDGLK (0) ITFPQEGDSAPNKTPADIVFIIR (1) IQVNPNHEIIKPTTTR (0) Multi-Hit GGQPGPDGGGQPGAYTYQFHGDPR (0) EIAEAYEVLSDK (0) | 302.51 |  |
| 102 | CG4183 | Hsp-26 | 22994 | 2D | 7 peptides matched SPIYELGLGLHPHSR (0) VVDDSILVEGK (0) IIQIQQVGPAHLNVK (0) Multi-Hit SINGCPCASPICPSSPAGQVLALRR (1) YVLPLGTQQRR (1) AEQVVSQLSSDGVLTVSIPKPQAVEDK (0) SPIYELGLGLHPHSRYVLPLGTQQR (1) | 224.59 |  |
| 103 | CG8937 | Hsp-c1 | 70686 | Both | 1D: 4 peptides matched MKETAEAYLGK (1) + 1 Oxidation (M) VFAPEEISAMVLGK (0) + 1 Oxidation (M) VTHAVVTVPAYFNDAQR (0) DNHLLGKFDLTGIPPAPR (1)  2D: 3 peptides matched SINPDEAVAYGAAVQAAILHGDK (0) TTPSYVAFTESERLIGDAAK (1) IINEPTAAAIAYGLDK (0) | 1D: 167.19  2D: 99.96 | |
| 104 | CG1579 / CG4147 | Hsp-c3 | 72260 |  | 1D: 4 peptides matched MKETAEAYLGK (1) + 1 Oxidation (M) VFAPEEISAMVLGK (0) + 1 Oxidation (M) VTHAVVTVPAYFNDAQR (0) DNHLLGKFDLTGIPPAPR (1)  2D: 13 peptides matched DLEAIVQPVIAK (0) NQLTTNPENTVFDAK (0) AKFEELNLDLFR (1) EWSDTNVQHDIK (0) DNHLLGKFDLTGIPPAPR (1) IINEPTAAAIAYGLDKK (1) IINEPTAAAIAYGLDK (0) NELESYAYSLK (0) VTHAVVTVPAYFNDAQR (0) VFAPEEISAMVLGK (0) + 1 Oxidation (M) KVTHAVVTVPAYFNDAQR (1) SQVFSTASDNQHTVTIQVYEGERPMTK (0) + 1 Oxidation (M) Multi-Hit VLEDADMNKKDVHEIVLVGGSTR (2) + 1 Oxidation (M) | 1D: 167.19  2D: 517.77 | |
| 105 | CG4264 | Hsp-c4 | 71131 | Both | 1D: 7 peptides matched MKETAEAYLGK (1) + 1 Oxidation (M) NQVAMNPTQTIFDAK (0) + 1 Oxidation (M) LVTHFVQEFK (0) SVIHDIVLVGGSTR (0) STAGDTHLGGEDFDNR (0) TFFPEEISSMVLTK (0) + 1 Oxidation (M) TVTNAVITVPAYFNDSQR (0)  2D: 22 peptides matched FEELNADLFR (0) IINEPTAAAIAYGLDKK (1) Multi-Hit NQVAMNPTQTIFDAKR (1) ARFEELNADLFR (1) DAGTIAGLNVLR (0) SVIHDIVLVGGSTR (0) TFFPEEISSMVLTK (0) + 1 Oxidation (M) Multi-Hit LLQDLFNGK (0) LLQDLFNGKELNK (1) NGLESYCFNMKATLDEDNLKTK (2) + 1 Oxidation (M) DNNLLGKFELSGIPPAPR (1) LIGDAAKNQVAMNPTQTIFDAKR (2) + 1 Oxidation (M) Multi-Hit TTPSYVAFTDTERLIGDAAK (1) QKELEGVCNPIITK (1) STAGDTHLGGEDFDNR (0) KFDDAAVQSDMKHWPFEVVSADGKPK (2) + 1 Oxidation (M) TVTNAVITVPAYFNDSQR (0) STAGDTHLGGEDFDNRLVTHFVQEFKR (2) ISDSDRTTILDKCNETIK (2) NQVAMNPTQTIFDAK (0) + 1 Oxidation (M) LIGDAAKNQVAMNPTQTIFDAK (1) + 1 Oxidation (M) MKETAEAYLGKTVTNAVITVPAYFNDSQR (2) + 1 Oxidation (M) | 1D: 217.02  2D: 823.45 | |
| 106 | CG8542 | Hsp-c5 | 74066 | 2D | 7 peptides matched SKLESLVGDLIKR (2) ALSDAEVSKSEIGEVLLVGGMTR (1) + 1 Oxidation (M) SKLESLVGDLIK (1) NAVVTVPAYFNDSQR (0) QATKDAGQIAGLNVLR (1) DAGQIAGLNVLR (0) QAVTNSANTFYATKR (1) | 348.06 |  |
| 107 | CG4463 | Hsp-23 | 20629 | 2D | 5 peptides matched NPYLALVGPMEQQLR (0) + 1 Oxidation (M) QRNPYLALVGPMEQQLR (1) + 1 Oxidation (M) MSMVPFYEPYYCQR (0) + 2 Oxidation (M) IVQIQQVGPAHLNVKENPKEAVEQDNGNDK (2) VQDNSVLVEGNHEEREDDHGFITR (1) | 101.91 |  |
| 108 | CG4466 | Hsp-27 | 23616, 81865 | 2D | 13 peptides matched RTPFDLFENQK (1) SDLVNNLGTIAK (0) QLEINPDHPIVETLR (0) Multi-Hit YESLTDPSKLDSGK (1) TPFDLFENQK (0) HSQFIGYPIK (0) FHTSASGDDFCSLADYVSR (0) YESLTDPSKLDSGKELYIK (2) ELISNASDALDK (0) TAGTLTIIDTGIGMTK (0) + 1 Oxidation (M) TMELIEELTEDKENYKK (2) + 1 Oxidation (M) HVYFITGESKDQVSNSAFVERVK (2)a QLVSVTKEGLELPEDESEKK (2) | 527.47 |  |
| 109 | CG12101 | Hsp-60 | 60808 | 2D | 4 peptides matched AAVEEGIVPGGGTALLR (0) LEDVKVSDLGQVGEVVITKDDTLLLK (2) EDGAPAMPGMGGMGGMGGMGGMGGMM (0) + 3 Oxidation (M) GYISPYFINSSKGAK (1) | 30.14 |  |
| 110a | CG5436 | Hsp-68 | 69743 | 2D | 2 peptides matched STAGDTHLGGEDFDNR (0) IINEPTAAALAYGLDK (0) | 84.83 |  |
| 110b | CG31366 | Hsp-70Aa | 70159 | 1D | 4 peptides matched VEIIANDQGNR (0) STAGDTHLGGEDFDNR (0) Multi-Hit GVPQIEVTFDLDANGILNVSAKEMSTGKAK (2) IINEPTAAALAYGLDK (0) | 69.76 |  |
| 110c | CG18743 | Hsp-70Ab | 70159 | 1D | 4 peptides matched VEIIANDQGNR (0) STAGDTHLGGEDFDNR (0) Multi-Hit GVPQIEVTFDLDANGILNVSAKEMSTGKAK (2) IINEPTAAALAYGLDK (0) | 69.76 |  |
| 110d | CG31449 | Hsp-70Ba | 70195 | 1D | 4 peptides matched VEIIANDQGNR (0) STAGDTHLGGEDFDNR (0) Multi-Hit GVPQIEVTFDLDANGILNVSAKEMSTGKAK (2) IINEPTAAALAYGLDK (0) | 69.76 |  |
| 110e | CG31359 | Hsp-70Bb | 70195 | 1D | 4 peptides matched VEIIANDQGNR (0) STAGDTHLGGEDFDNR (0) Multi-Hit GVPQIEVTFDLDANGILNVSAKEMSTGKAK (2) IINEPTAAALAYGLDK (0) | 69.76 |  |
| 110f | CG6489 | Hsp-70Bc | 70195 | 1D | 4 peptides matched VEIIANDQGNR (0) STAGDTHLGGEDFDNR (0) Multi-Hit GVPQIEVTFDLDANGILNVSAKEMSTGKAK (2) IINEPTAAALAYGLDK (0) | 69.76 |  |
| 110g | CG5834 | Hsp70Bbb | 70253 | 1D | 4 peptides matched VEIIANDQGNR (0) STAGDTHLGGEDFDNR (0) Multi-Hit GVPQIEVTFDLDANGILNVSAKEMSTGKAK (2) IINEPTAAALAYGLDK (0) | 69.76 |  |
| 111 | CG5374 | Tcp1-like | 59556 | 2D | 20 peptides matched MLVDDIGDVTVTNDGATILR (0) SGKSYADACAAGELDG (1) MGVQVLINDPDKLEAIR (1) + 1 Oxidation (M) Multi-Hit QKIHPTSIISGYR (1) TLSVNAAKDATDLVAKLR (2) TSMSSKIIGADAEFFSAMVVDAAQSVK (1) + 2 Oxidation (M) IACLDFSLQKTK (1) Multi-Hit TQNVMAALSISNIVK (0) GPNDFYCDEMER (0) + 1 Oxidation (M) ICDDELILIKGTK (1) ESVLIPGYALNCTIASQQMPK (0) + 1 Oxidation (M) Multi-Hit MKMGVQVLINDPDKLEAIR (2) + 2 Oxidation (M) DSLINIAK (0) SARESVLIPGYALNCTIASQQMPK (1) + 1 Oxidation (M) QSGASVRTQNVMAALSISNIVKSSLGPVGLDK (2) + 1 Oxidation (M) IIGADAEFFSAMVVDAAQSVK (0) + 1 Oxidation (M) Multi-Hit YISEHLTAPVDELGRDSLINIAK (1) MGVQVLINDPDKLEAIRAR (2) + 1 Oxidation (M) SLLVIPKTLSVNAAKDATDLVAK (2) IHPTSIISGYR (0) | 724.43 |  |
| 112 | CG8439 | Cct-5 | 59278 | 2D | 22 peptides matched IADGFELAAQCAIK (0) WVGGPEIELIAIATGGR (0) EADQLSTLEQYAFR (0) MILKIDDVR (1) + 1 Oxidation (M) ITGTDAIKTHIMAAR (1) QMAEMAVDAVLNVADIEKK (1) + 2 Oxidation (M) NKEPLIQIAMTTLGSK (1) Multi-Hit QQILLSTQLVK (0) NVKLAILTCPFEPPKPK (1) QLDAIAQPFPVDPK (0) KQQILLSTQLVK (1) SSLGVDCMLSGDSDMK (0) + 2 Oxidation (M) IVPRFEELTPEKLGVAGLVR (2) HKLDVTSAEDYR (1) DVNFELIKIETK (1) ALREYEQEKFTQMVK (2) Multi-Hit GLDKIMVSPDGDVTVTNDGATIMK (1) + 2 Oxidation (M) IVYGGGAAEISCSLAVAK (0) TKHKLDVTSAEDYR (2) IMVSPDGDVTVTNDGATIMK (0) + 2 Oxidation (M) QLDAIAQPFPVDPKNKEPLIQIAMTTLGSK (2) + 1 Oxidation (M) SHNVVESLHSKKQQILLSTQLVK (2) | 887.19 |  |
| 113 | CG8977 | Cctγ | 59394 | 2D | 16 peptides matched GPYTAVAHALEIIPR (0) LVAGGGAVEMAASQLLTR (0) + 1 Oxidation (M) Multi-Hit TAVETAILLLR (0) MLQIEEEFVQR (0) + 1 Oxidation (M) Multi-Hit YAKVEKIPGGAIEESCVLK (2) GASKDILNETERNLQDALHVAR (2) Multi-Hit MLMDPMGGIVMTNDGNAILR (0) + 4 Oxidation (M) Multi-Hit DILNETERNLQDALHVAR (1) KGESQTNVEIIGEQDFTR (1) GVSDLAQHYLLK (0) MLMDPMGGIVMTNDGNAILREITVQHPAAK (1) + 4 Oxidation (M) TAVETAILLLRIDDIVSGSK (1) ACGATIVNRTEELTEKDVGTGAGLFEVK (2) FIGKWSDLAVK (1) NLQDALHVAR (0) HASHTGDGVCAWGIDGESGEIVDMNVK (0) + 1 Oxidation (M) | 682.46 |  |
| PROTEIN DEGRADATION | |  |  |  |  |  |  |
| 114 | CG1519 | Pros-α7 | 27675 | 2D | 3 peptides matched IEPSGSSFGYFACASGK (0) VTGGLHLINPSELTEK (0) VFQIDYASK (0) | 172.68 |  |
| 115 | CG3422 | Pros-28.1 | 27973 | 2D | 2 peptides matched MLDTDVITDYVK (0) + 1 Oxidation (M) AVTIFSPDGHLLQVEYAQEAVR (0) | 84.28 |  |
| 116 | CG9327 | Pros 29 | 29411 | 2D | 10 peptides matched LNDNMVCSVAGITSDANVLTSELR (0) + 1 Oxidation (M) LTLADAKDLAIK (1) IYRLNDNMVCSVAGITSDANVLTSELR (1) + 1 Oxidation (M) STNKLLDSAIPSEKIYR (2) LTPEKVEMATLQR (1) + 1 Oxidation (M) VDNKTVYSVLEKPDVEK (1) YQFSYGEVIPCEQLVSHLCDIK (0) TVYSVLEKPDVEKLIEK (1) STNKLLDSAIPSEK (1) VLSMTLDTTKLTPEKVEMATLQR (2) + 2 Oxidation (M) | 362.12 |  |
| 117 | CG4904 | Pros-35 | 31058 | 2D | 5 peptides matched GPHIYQVTPSATFFNCK (0) YLRSECLNYKHSYDTTYPVSR (2) NQYDSDVTVWSPQGR (0) LHQVEYAMEAVK (0) + 1 Oxidation (M) DQPFTILSNKDSAK (1) | 120.71 |  |
| 118 | CG1489 | Pros-45 / Rpt6 | 45857 | 2D | 5 peptides matched EHAPSIIFMDEIDSIGSSR (0) + 1 Oxidation (M) GVLLYGPPGTGK (0) IAELMPGASGAEVK (0) + 1 Oxidation (M) GVCTEAGMYALR (0) + 1 Oxidation (M) FVVDLDKNIDINDVTPNCR (1) | 181.63 |  |
| 119 | CG3455 | Rpt4b | 44824 | 2D | 11 peptides matched VALDMTTLTIMR (0) + 2 Oxidation (M) VVSSAIVDKYIGESAR (1) HGEIDYEAIVK (0) NVCTEAGLFAIR (0) TVTATPMPDNLR (0) Multi-Hit LSDNFNGADLR (0) FSEGTSADREIQR (1) IEIPLPNEQAR (0) DHQPCIIFMDEIDAIGGR (0) + 1 Oxidation (M) LIREMFNYAR (1) + 1 Oxidation (M) TLMELLNQMDGFDSLGQVK (0) + 2 Oxidation (M) | 376.95 |  |
| 120 | CG2241 | Rpt6b | 45169 | 2D | 3 peptides matched GVLLYGPPGTGK (0) GVCTEAGMYALR (0) + 1 Oxidation (M) IAEEMPGASGAEVK (0) | 92.07 |  |
| 121 | CG10149 | Pros 44.5 / Rpn6 | 47263 | 2D | 4 peptides matched IMLGQSDDVNQLVSGK (0) + 1 Oxidation (M) IKEQGILQQGELYKQEGK (2) NLLVEVQLLESK (0) VQVAHVAESIQLPMPQVEK (0) + 1 Oxidation (M) | 149.94 |  |
| 122 | CG18174 | Rpn11 | 34400 | 2D | 5 peptides matched LINPNMLVLGQEPR (0) Multi-Hit AVAVVVDPIQSVK (0) VVIDAFR (0) SLEDEEKMTPEQLAIK (1) + 1 Oxidation (M) QTTSNLGHLQKPSVQALIHGLNR (0) | 199.64 |  |
| 123a* | CG11624 | Ubiquitin p / Ubi63E | 85798 | 1D | 2 peptides matched TITLEVEPSDTIENVK (0) TLSDYNIQKESTLHLVLR (1) | 56.91 |  |
| 123b* | CG2960 | Ubiquitin f / Ribosomal protein L40 | 14729 | 1D | 2 peptides matched TITLEVEPSDTIENVK (0) TLSDYNIQKESTLHLVLR (1) | 66.98 |  |
| 123c* | CG5271 | Ubiquitin m / Ribosomal protein S27A | 17939 | 1D | 2 peptides matched TITLEVEPSDTIENVK (0) TLSDYNIQKESTLHLVLR (1) | 66.98 |  |
| 124 | CG4265 | Ubiquitin c-terminal hydrolase | 25850 | 2D | 1 peptides matched AFILLFPCSETYEK (0) | 45.88 |  |
| 125 | CG1548 | cathepsin D | 42472 | 2D | 7 peptides matched GGCQVIADTGTSLIAAPLEEATSINQK (0) VVFDTGSSNLWVPSKK (1) MDAASIGDLQLCK (0) + 1 Oxidation (M) Multi-Hit FDGILGLGYNSISVDK (0) YYTEFDMGNDR (0) + 1 Oxidation (M) VVFDTGSSNLWVPSK (0) TFELEGKDYILR (1) | 190.96 |  |
| 126 | CG8947 | 26/29kD-proteinase | 62129 | 2D | 7 peptides matched IDYYGGMVKTYQLAGEGQYGTLLK (1) + 1 Oxidation (M) Multi-Hit KNIYTLWVR (1) TYQLAGEGQYGTLLKLAPITTK (1) Multi-Hit TYQLAGEGQYGTLLK (0) NIYTLWVR (0) SRIDYYGGMVK (1) MPIPVRYEMR (1) Multi-Hit | 286.22 |  |
| 127a* | CG7486 | caspase / dredd | 13903 | 2D | 1 peptides matched IGELVESER (0) | 47.98 |  |
|  |  |  |  |  |  |  |  |
| METABOLISM | |  |  |  |  |  |  |
| 127b* | CG2140 | Cyt-b5 | 15153 | 2D | 1 peptides matched IGELVESER (0) | 50.8 |  |
| 128 | CG7113 | scully | 26904 | 2D | 13 peptides matched LSAGLMGANEPNQDGQR (0) Multi-Hit NAVSLVTGGASGLGR (0) AAVVGMTLPIAR (0) Multi-Hit QGASVILADLPSSK (0) VININTVGTFNVIR (0) LDLTVNCAGTATAVK (0) QGASVILADLPSSKGNEVAK (1) VVFVPVDVTSEK (0) ELGDKVVFVPVDVTSEK (1) VVFVPVDVTSEKDVSAALQTAK (1) ICTIAPGLFNTPMLAALPEKVR (1) + 1 Oxidation (M) NVAHRLEDFQR (1) ELGDKVVFVPVDVTSEKDVSAALQTAK (2) | 203.54, 575.55 | |
| 129 | CG8893 | GAP Dehydrogenase 2 | 33770, 35369, 35383 | Both | 1D: 3 peptides matched VPTPNVSVVDLTVR (0) GAAQNIIPASTGAAK (0) IGINGFGRIGR (1)  2D: 2 peptides matched VVSNASCTTNCLAPLAK (0) GAAQNIIPASTGAAK (0) | 1D: 22.19  2D: 74.78 | |
| 130 | CG14476 | Glucosidase II | 105731 | 2D | 3 peptides matched VLFGIPEHADSFILK (0) STSGTDPYRLYNLDVFEYVVDSK (1) TPPPAAHFMSESGIVDAFIMLGPKPMDTFK (0) + 3 Oxidation (M) | 39.47 |  |
| 131 | CG1633 | Thioredoxin Peroxidase 1 | 21737 | 2D | 11 peptides matched DYGVLDEETGIPFR (0) QITVNDLPVGR (0) PQLQKPAPAFAGTAVVNGVFK (0) PQLQKPAPAFAGTAVVNGVFKDIK (1) Multi-Hit KQGGLGSMDIPLLADKSMK (2) QGGLGSMDIPLLADKSMK (1) + 2 Oxidation (M) QITVNDLPVGRSVEETLR (1) LVQAFQYTDKYGEVCPANWKPGQK (1) TMVADPTKSKEYFETTS (2) + 1 Oxidation (M) Multi-Hit INCEVIGCSTDSQFTHLAWINTPR (0) KINCEVIGCSTDSQFTHLAWINTPR (1) | 392.9 |  |
| 132 | CG3593 | rudimentary-like | 53419 | 2D | 1 peptides matched VGINSPVYFDLR (0) | 39.51 |  |
| 133 | CG3481 | Alcohol Dehydrogenase | 27629, 14497, 27513 | 2D | 7 peptides matched TVDVLINGAGILDDHQIER (0) Multi-Hit AAVVNFTSSLAK (0) NVIFVAGLGGIGLDTSKELLKR (2) LLAHPTQPSLACAENFVK (0) LAPITGVTAYTVNPGITR (0) NLVILDRIENPAAIAELK (1) Multi-Hit DLKNLVILDRIENPAAIAELK (2) Multi-Hit | 265.36 |  |
| 134 | CG4581 | Thiolase | 50641 | 2D | 22 peptides matched VSGVDQIVDKDNGIR (1) LRPAFVKPYGTVTAANASFLTDGASACIIMTEEK (0) + 1 Oxidation (M) VSGVDQIVDK (0) ELIDYIVYGSVIQEVK (0) HSLLSLLQK (0) TPFLTSGTTYSK (0) GYFTDLVPFKVSGVDQIVDKDNGIR (2) NSQNIVLVDGVR (0) SEQDEYALR (0) DFLFVSQDPVNQLLLGPAYGIPK (0) Multi-Hit GYFTDLVPFK (0) QKNSQNIVLVDGVR (1) ALDSDWFCK (0) EAALAAGFSNK (0) WNNWGGSLSIGHPFAATGVR (0) LGVVAACAAGGQGVAMLIER (0) Multi-Hit LGVVAACAAGGQGVAMLIERYPGATAD (1) + 1 Oxidation (M) Multi-Hit NSQNIVLVDGVRTPFLTSGTTYSK (1) NRLDKELIDYIVYGSVIQEVK (2) LALLSTFRPDFLAPELPAVAEFSSGETMGHSADR (0) + 1 Oxidation (M) ALDSDWFCKTYLGLNEK (1) VGTPDLSKWNNWGGSLSIGHPFAATGVR (1) | 904.24 |  |
| 135 | CG7660 | Dpxt | 93000 | Both | 1D: 4 peptides matched VTHFVDASPVYGSSDEASR (0) LSLVPSPDCQLSYGK (0) FLFRGDNPFGLDLAAINIQR (1) FKQGDRYYYEYDNGINPGAFNPLQLQEIR (2)  2D: 22 peptides matched VAGALHELNPSASDETLFQEAR (0) EQVPVEDFITNNVPIK (0) VTHFVDASPVYGSSDEASR (0) RSGFPTILSPAVLDEARR (2) RSGFPTILSPAVLDEAR (1) MLPPAYEDGIWTPR (0) + 1 Oxidation (M) TPDDIDLWVGGLLEK (0) TLYSQPMQQVDSSISQGLSR (0) + 1 Oxidation (M) QLTKVTHFVDASPVYGSSDEASR (1) Multi-Hit SGFPTILSPAVLDEAR (0) LLCDNSDRLTLQAVPLAAFVR (1) SLWGAAGQPMER (0) + 1 Oxidation (M) LHSFEQFPIEIAQKLSR (1) LEDGSLVQCCSPEGK (0) LTLQAVPLAAFVR (0) FLFRGDNPFGLDLAAINIQR (1) TLLSDVDRPHPK (0) LVPLHQGYSHDYNVNVNPAITNEFSGAAYR (0) QEHGRIDEVVNIPDVMFNPSR (1) + 1 Oxidation (M) Multi-Hit SMDGTCNNPEPQRSLWGAAGQPMER (1) + 2 Oxidation (M) EFYDDMLR (0) + 1 Oxidation (M) ADHPGNQMIGCDDPNLPSVNLEAWRA (1) + 1 Oxidation (M) | 1D: 35.55  2D: 871.15 | |
| 136 | CG9681 | PGRP-SB1 | 20997 | Both | 1D: 4 peptides matched SIGIVFIGNFER (0) NFSDIGYNFIVAGDGKVYEGR (1) GYLKDNYTLFGHR (1) DLIELAK (0)  2D: 4 peptides matched ATSCPGDALYNEIK (0) SIGIVFIGNFER (0) GYLKDNYTLFGHR (1) NFSDIGYNFIVAGDGK (0) | 1D: 149.97  2D: 143.16 | |
| 137 | CG11661 | Nc73EF | 112585 | 1D | 14 peptides matched LAVVANPSHLEAVDPVVQGK (0) LNTLANVCR (0) IIPDNGPAGQNPSNVK (0) FLQMSSDDPDYFPPESDEFGVR (0) + 1 Oxidation (M) ATGFEAFLAK (0) VYYDLTK (0) SVAAKYENICEEAFALAK (1) GHNIAHLDPLEINTPELPGNSSTK (0) NGHNEIDEPMFTQPLMYQK (0) + 2 Oxidation (M) SPFSEMSEGSEFQR (0) + 1 Oxidation (M) VADWALGEAMAFGSLLK (0) + 1 Oxidation (M) FSSPPPNAAEFVIHK (0) VVNAPIFHVNADDPEAVMHVCK (0) + 1 Oxidation (M) QLHDINWIVANCSTPANYYHILR (0) | 370.86 |  |
| 138 | CG7642 | rosy | 84769 | 2D | 4 peptides matched ESEESIYFGAAVSLMEIDALLRK (1) LRRPVRSMLDR (2) LEVASLVDGK (0) NVIEPNEVLVGIHFQK (0) | 37.97 |  |
| NUCLEAR ENVELOPE | | |  |  |  |  |  |
| 139 | CG4799 | Pendulin / Importin α2 | 57821 | 1D | 3 peptides matched IQAVVDSDAVPR (0) LLQMDEPSIIVPALR (0) + 1 Oxidation (M) EAAWTVSNITAGNQK (0) | 149.65 |  |
| 140 | CG2637 | fs(2)Ketel / Importin β | 98695 | 1D | 2 peptides matched LYPLLDDAIITQFLAEGKR (1) GALQFLTPVLVEK (0) | 74.5 |  |
| 141 | CG1059 | Karyopherin β3 | 123561 | 1D | 11 peptides matched FIIQNANSDDLR (0) EIENISPTENAISAFAK (0) IVSIIAESFCTK (0) TAAAESLPYLLDCAK (0) YAACNAIGQMSTDFAQTFEKK (1) + 1 Oxidation (M) LLIEMTENCPK (0) + 1 Oxidation (M) LAARNEEDYDDGVEEELAEQDDTDVYILSK (1) VIVTEPEPDVQSELLNSLAK (0) FHSQVIPGLLSLLDDVENPR (0) IFSSVPSIFGNQEAQYIDLIKQMLAK (1) + 1 Oxidation (M) IVLPLVMNALPVMLGHADWK (0) + 2 Oxidation (M) | 341.18 |  |
| 142 | CG3820 | Nup214 / CAN | 175175 | 1D | 1 peptides matched LQNLTSNQR (0) | 37.45 |  |
| 143 | CG11856 | Nup358 / RanBP2 | 296385 | 1D | 4 peptides matched IGQYEQFLDEVR (0) EVYDSSNLGNR (0) SALPLLLLGYQVRPIDDSSTNQWIK (0) VRDDAVGHNMIATCYSR (1) + 1 Oxidation (M) | 115 |  |
| 144 | CG9710 | NudC | 37791 | 2D | 3 peptides matched QQLLDSAGGEPSASNR (0) TEESVWVLQDSK (0) DGISKPIEKVDDESDKSELGK (2) | 128.77 |  |
| 145 | CG6944 | Lamin | 71300 | 2D | 4 peptides matched TILLNSEGEAVANLDR (0) NIFEAELLETR (0) AVVDESEDHSVADYYVSASAK (0) VRIDALNANINELEQANADLNAR (1) | 183.8 |  |
|  |  |  |  |  |  |  |  |
| MITOCHONDRIA | |  |  |  |  |  |  |
| 146 | CG10691 | lethal (2) 37Cc | 30383 | Both | 1D: 3 peptides matched IGQMGLGVAVLGGVVNSALYNVEGGHR (0) + 1 Oxidation (M) SFGEAGDGLVELR (0) LASIISAEGDAEAAGLLAK (0)  2D: 8 peptides matched AVVAQFDAGELITQR (0) SFGEAGDGLVELR (0) NVPVITGSKDLQNVNITLR (1) LASIISAEGDAEAAGLLAK (0) ILYRPIPDQLPK (0) Multi-Hit AVVAQFDAGELITQREMVSQR (1) + 1 Oxidation (M) SRGVAYLPSGQSTLLNLPSTIAQ (1) GVAYLPSGQSTLLNLPSTIAQ (0) | 1D: 86.59  2D: 401.27 | |
| 147 | CG2151 | Thioredoxin Reductase-1 | 64322 | 2D | 2 peptides matched GLGYEPTVMVR (0) + 1 Oxidation (M) GLVDDLNLPNAGVTVQK (0) | 31.14 |  |
| 148 | CG3283 | Succinate Dehydrogenase | 33740 | 2D | 1 peptides matched DLVPDMNNFYEQYR (0) + 1 Oxidation (M) | 31.26 |  |
| 149 | CG4169 | Ubiquinol-Cytochrome c  Reductase | 45415 | 1D | 2 peptides matched TVVNQLNAVSTEER (0) LAVGAIGHLANVPYASDLA (0) | 83 |  |
| 150 | CG4600 | yippee interacting protein 2 | 43597 | 2D | 17 peptides matched YKEIGGGSPILK (1) Multi-Hit YVNPLTENTLAEIEKDKPER (1) Multi-Hit RNDVVILFTAHSLPLK (1) Multi-Hit LGPWIAQR (0) EIGGGSPILK (0) TAILMLNMGGPTHTDQVHDYLLR (0) + 2 Oxidation (M) Multi-Hit VVLFSQYPQYSCATSGSSFNSIFTHYR (0) YVNPLTENTLAEIEK (0) VGPLPWLAPATDDAIKGYVK (1) NDVVILFTAHSLPLK (0) IRDELAKFVETK (2) AATPNDHPLFIDALTNVVADHLK (0) Multi-Hit EVGVEEIR (0) IMTDRDMIQLPVQSR (1) + 2 Oxidation (M) ISPETAPHKHYVGFR (1) RAATPNDHPLFIDALTNVVADHLK (1) SNNLPSDIKWSIIDRWGTHPLLIK (2) | 688.39 |  |
| 151 | CG6439 | Isocitrate Dehydrogenase | 40381 | Both | 1D: 9 peptides matched HTNIDTVIIR (0) LRNDLDLYANVVHVR (1) TKDLGGQSTTQDFTR (1) LGDGLFLR (0) HINLPTYGEIIQNAINK (0) FAFDYATK (0) DLGGQSTTQDFTR (0) HINLPTYGEIIQNAINKVLNDGK (1) GVLATPDYSNVGDLQTLNMK (0) + 1 Oxidation (M)  2D: 8 peptides matched DLGGQSTTQDFTR (0) LGDGLFLR (0) GVLATPDYSNVGDLQTLNMK (0) + 1 Oxidation (M) LRNDLDLYANVVHVR (1) FAFDYATK (0) SCEEVSRLYPR (1) Multi-Hit NVANPTAMLLCGVK (0) + 1 Oxidation (M) IAKFAFDYATK (1) | 1D: 344.72  2D: 315.0 | |
| 152 | CG6647 | Porin | 30550 | Both | 1D: 7 peptides matched VNNASQVGLGYQQK (0) YQLDDDASVR (0) DGVTLTLSTLVDGK (0) ADSDVNIDLK (0) APPSYSDLGK (0) TSSGIEFNTAGHSNQESGK (0) DFVLHTAVNDGQEFSGSIFQR (0)  2D: 9 peptides matched DGVTLTLSTLVDGK (0) AKVNNASQVGLGYQQKLR (2) YQLDDDASVR (0) DIFSKGYNFGLWKLDLK (2) GYNFGLWKLDLK (1) DGVTLTLSTLVDGKNFNAGGHK (1) LRDGVTLTLSTLVDGKNFNAGGHK (2) LTTNNFALGYTTK (0) LRDGVTLTLSTLVDGK (1) | 1D: 243.17  2D: 266.48 | |
| 153 | CG7361 | Phosphodiesterase 9 | 24872 | 2D | 2 peptides matched KGPAPLNLEVPTHEFPNEGLLVVG (1) IRKGPAPLNLEVPTHEFPNEGLLVVG (2) | 35.3 |  |
| 154 | CG8470 | mitochondrial ribosomal protein S30 | 65107 | 2D | 5 peptides matched GTKPLPLYAEISASGK (0) MVQYTGAPYLALR (0) + 1 Oxidation (M) GNENNFGLLSFQSR (0) SFGEQDQLEALHSLAIK (0) TVLEDLPSETDKK (1) | 147.31 |  |
| 155 | CG2098 | Ferrochelatase | 43597 | 2D | 8 peptides matched YVNPLTENTLAEIEKDKPER (1) Multi-Hit LGPWIAQR (0) RNDVVILFTAHSLPLK (1) VGPLPWLAPATDDAIKGYVK (1) TAILMLNMGGPTHTDQVHDYLLR (0) + 2 Oxidation (M) Multi-Hit AATPNDHPLFIDALTNVVADHLKSQQAVNPK (1) IMTDRDMIQLPVQSR (1) + 2 Oxidation (M) AATPNDHPLFIDALTNVVADHLK (0) Multi-Hit | 298.07 |  |
| 156 | CG3612 | ATP synthase α / bellwether | 59421 | 2D | 20 peptides matched TALAIDTIINQK (0) VVDALGNAIDGK (0) HALIIYDDLSK (0) SAEISNILEER (0) GIRPAINVGLSVSR (0) SAEISNILEERILGVAPK (1) TGAIVDVPVGDELLGR (0) QVAGSMKLELAQYR (1) + 1 Oxidation (M) VLSIGDGIAR (0) ILGVAPKADLEETGRVLSIGDGIAR (2) Multi-Hit ADLEETGRVLSIGDGIAR (1) HALIIYDDLSKQAVAYR (1) AMKQVAGSMKLELAQYR (2) + 2 Oxidation (M) GMALNLEPDNVGVVVFGNDKLIK (1) + 1 Oxidation (M) SAEISNILEERILGVAPKADLEETGR (2) QGQYVPMAIEDQVAVIYCGVR (0) + 1 Oxidation (M) EVAAFAQFGSDLDAATQQLLNR (0) TSEQALLDTIAK (0) HALIIYDDLSKQAVAYRQMSLLLR (2) + 1 Oxidation (M) RPPGREAYPGDVFYLHSR (1) | 727.13 |  |
| 157 | CG6030 | ATP synthase δ | 20200 | 2D | 6 peptides matched LVPVAGLVDSFQK (0) IAQSSINWSALAERVPANQK (1) IQNYQKEIAHLK (1) AVLANPECPPQIDWANYK (0) SLLPYDQMTMEDYR (0) + 2 Oxidation (M) AVLANPECPPQIDWANYKK (1) | 156.72 |  |
|  |  |  |  |  |  |  |  |
| OTHER |  |  |  |  |  |  |  |
| 158 | CG10844 | Ryanodine Receptor | 579487 | 1D | 12 peptides matched SSFKTATRDIK (2) EMQNMAER (0) + 1 Oxidation (M) FGVHMLSPANIK (0) + 1 Oxidation (M) YGDTTVIVQHCETSLWLSYKSYETKK (2) KEEASIATTTFCLRQEK (2) SFPVNYWDKFVK (1) GYPDLGWDPVEGER (0) CSSLFTQFITALETLQSNRR (1) GASQGMFVGCFVDTATGIIR (0) GSLTRNITFETDMSAALDEMQR (1) + 2 Oxidation (M) NTKAEGLGVGSEGAAGEKNMHDAEFTCALFR (2) HPRLKPYNMLSEYER (1) | 37.83 |  |
| 159 | CG2985 | Yolk protein 1 | 48711 | 2D | 6 peptides matched TVAPLNEMIQR (0) + 1 Oxidation (M) AYMGIDTAHDLEGDYILQVNPK (0) + 1 Oxidation (M) NAPAQKQSSYHGVHQAWNTNQDSKDYQ (2) SGDVDFYPNGPAAGVPGASNVVEAAMR (0) + 1 Oxidation (M) Multi-Hit SFPAVPANSLQQYKQNDGFGKR (2) Multi-Hit SGDVDFYPNGPAAGVPGASNVVEAAMRATR (1) + 1 Oxidation (M) | 159.93 |  |
| 160 | CG2979 | Yolk protein 2 | 49660 | Both | 1D: 2 peptides matched LVELTNTVNVPQEIIHLIGSGPAAHVAGVAGR (0) GDADFVDAIHTSAYGMGTSQR (0) + 1 Oxidation (M)  2D: 8 peptides matched RITALDPTKIYGKPEER (2) LANVDFFPNGPSTGVPGADNVVEATMR (0) + 1 Oxidation (M) QQKFGDDEVTIFIQGLPETNTQVQK (1) NFPSVAASSYQEYKQNK (1) YFAESVRPGNER (0) LTGLARGDADFVDAIHTSAYGMGTSQR (1) + 1 Oxidation (M) LANVDFFPNGPSTGVPGADNVVEATMRATR (1) + 1 Oxidation (M) GDADFVDAIHTSAYGMGTSQR (0) + 1 Oxidation (M) | 1D: 28.71  2D: 149.12 | |
| 161 | CG11129 | Yolk protein 3 | 52173 | Both | 1D: 11 peptides matched AYMGLQIDYDLR (0) + 1 Oxidation (M) AQPGFGEDEVTIVLTGLPK (0) LIQAYVQK (0) AASGDLIIIDLGSTLTNFKR (1) YNLQQLQK (0) YAMLDVLNTGAMIGQTLIDLTNK (0) + 2 Oxidation (M) Multi-Hit GVPQEIIHLIGQGISAHVAGAAGNK (0) NFPAVPANSLK (0) GDADFVDAIHTSTFAMGTPIR (0) + 1 Oxidation (M) YFAESVRPGSER (0) Multi-Hit SPAHKQAAYHGMHHAQN (1)  2D: 24 peptides matched AQPGFGEDEVTIVLTGLPK (0) AASGDLIIIDLGSTLTNFKR (1) Multi-Hit AASGDLIIIDLGSTLTNFK (0) LNNYVETAKAQPGFGEDEVTIVLTGLPK (1) YAMLDVLNTGAMIGQTLIDLTNK (0) + 2 Oxidation (M) Multi-Hit RLIQAYVQKYNLQQLQK (2) RITGLDPAKVLSK (2) GVPQEIIHLIGQGISAHVAGAAGNK (0) Multi-Hit LIQAYVQKYNLQQLQK (1) Multi-Hit SAKAASGDLIIIDLGSTLTNFKR (2) NFPAVPANSLK (0) AQPGFGEDEVTIVLTGLPKTSPAQQK (1) CGDVDFYPNGPSTGVPGSENVIEAVARATR (1) YFAESVRPGSERNFPAVPANSLK (1) Multi-Hit VIEKIYHVGQIKHDLTPSFVPSPSNVPVWIIK (2) RPQILGGLSRGDADFVDAIHTSTFAMGTPIR (1) + 1 Oxidation (M) AQPGFGEDEVTIVLTGLPKTSPAQQKAMR (2) Multi-Hit HDLTPSFVPSPSNVPVWIIKSNGQKVECK (2) CGDVDFYPNGPSTGVPGSENVIEAVAR (0) GDADFVDAIHTSTFAMGTPIRCGDVDFYPNGPSTGVPGSENVIEAVAR (1) + 1 Oxidation (M) SNGQKVECKLNNYVETAK (2) IYHVGQIKHDLTPSFVPSPSNVPVWIIK (1) VLSKRPQILGGLSRGDADFVDAIHTSTFAMGTPIR (2) + 1 Oxidation (M) ATRYFAESVRPGSER (1) | 1D: 397.85  2D: 803.16 | |
| 162 | CG12357 | cap binding protein 20 | 17718 | 2D | 3 peptides matched VDWDAGFVEGR (0) LLSQKIAPNTDNR (1) VIVMGLDKYKK (2) + 1 Oxidation (M) | 63.69 |  |
| 163 | CG17437 | will die slowly | 39041 | 2D | 6 peptides matched TLIDDDNPPVSFVK (0) LGISDVAWSSDSR (0) IWDTASGQCLK (0) YILAATLDNTLK (0) IWGAYDGKFEK (1) SSLSVKPNYTLK (0) | 249.25 |  |
| 164 | CG5519 | Gbp | 55198 | 2D | 4 peptides matched VFNDHTALATGVR (0) TIQLDDGYEVK (0) VIEKYLLENGCDPISGK (1) TPAVVKPKPPSATSIPATLK (0) | 146.36 |  |
| 165 | CG6226 | FK506 binding protein 1 | 39343 | Both | 1D: 3 peptides matched AIPQVALDLNFSK (0) IVDQVVGKGEEAK (1) FALGGGEVIK (0)  2D: 13 peptides matched AIPQVALDLNFSK (0) SFHISGVALDKGQEAK (1) AIPQVALDLNFSKGDR (1) GWDVGVAGMK (0) + 1 Oxidation (M) Multi-Hit FALGGGEVIK (0) LYLAAEKQEYIVATVTK (1) VITCPPHMAYGAR (0) Multi-Hit VSVYYIGR (0) IVDQVVGKGEEAK (1) Multi-Hit TFDSLLK (0) LQSNNKTFDSLLK (1) TITGGVKIVDQVVGKGEEAK (2) FALGGGEVIKGWDVGVAGMK (1) + 1 Oxidation (M) | 1D: 108.06  2D: 448.56 | |
| 166 | CG7111 | RACK 1 | 35617 | Both | 1D: 1 peptides matched DVLSVAFSADNR (0)  2D: 6 peptides matched ALLWDLNDGK (0) DVLSVAFSADNR (0) GTLIGHNGWVTQIATNPK (0) TVEELRPEVVSPTSK (0) Multi-Hit FSPNHSNPIIVSCGWDR (0) NNHHGHNGYLNTVTVSPDGSLCTSGGKDSK (1) | 1D: 59.95  2D: 255.81 | |
| 167 | CG7269 | Hel25E | 48651 | 2D | 4 peptides matched ILVATNLFGR (0) GLAITFVSDENDAK (0) QVMMFSATLSK (0) + 2 Oxidation (M) VAVFFGGMAIQKDEETLK (1) + 1 Oxidation (M) | 138.9 |  |
| 168 | CG8472 | Calmodulin | 16679 | 2D | 2 peptides matched VFDKDGNGFISAAELR (1) SLGQNPTEAELQDMINEVDADGNGTIDFPEFLTMMAR (0) + 3 Oxidation (M) | 87.28 |  |
|  |  |  |  |  |  |  |  |
| 169 | CG9748 | belle | 85099 | Both | 1D: 10 peptides matched SGDCPILVATAVAAR (0) ELATQIFEEAK (0) IVEQLNMPPTGQR (0) + 1 Oxidation (M) HVINFDLPSDVEEYVHR (0) ELATQIFEEAKK (1) TAAFLVPILNQMYELGHVPPPQSTR (0) + 1 Oxidation (M) MRPAVLYGGNNTSEQMR (0) + 2 Oxidation (M) MLDMGFEPQIR (0) + 2 Oxidation (M) FLVLDEADRMLDMGFEPQIR (1) + 2 Oxidation (M) SYLLDLLSSIR (0)  2D: 15 peptides matched SGDCPILVATAVAAR (0) HVINFDLPSDVEEYVHR (0) MGNLGVATSFFNEKNR (1) + 1 Oxidation (M) IVEQLNMPPTGQR (0) + 1 Oxidation (M) QTLMFSATFPK (0) + 1 Oxidation (M) HAIPIIINGR (0) TAAFLVPILNQMYELGHVPPPQSTR (0) + 1 Oxidation (M) GGGGRFEDNYNGGEFDSR (1) QYPLGLVLAPTR (0) VGSTSENITQTILWVYEPDKR (1) MRPAVLYGGNNTSEQMR (0) + 2 Oxidation (M) KGADSLEEFLYQCNHPVTSIHGDR (1) MLDMGFEPQIR (0) + 2 Oxidation (M) FLVLDEADRMLDMGFEPQIR (1) + 2 Oxidation (M) FLVLDEADRMLDMGFEPQIRR (2) + 2 Oxidation (M) | 1D: 332.27  2D: 464.97 | |
| 170 | CG9916 | Cyclophilin 1 | 20963 | Both | 1D: 1 peptides matched VFFDMTADNEPLGR (0) + 1 Oxidation (M)  2D: 3 peptides matched VFFDMTADNEPLGR (0) + 1 Oxidation (M) VIPNFMCQGGDFTNHNGTGGK (0) SIYGNKFPDENFELK (1) | 1D: 27.11  2D: 121.36 | |
| 171 | CG6143 | Protein on ecdysone puffs | 75599 | 1D | 7 peptides matched AAAPAAVASPAAAATSADASPSPAKK (1) FFDTEVTAEIHSR (0) QTLPISTEEEETR (0) LECYECSVCSK (0) EGIEESYRLK (1) NFGGGNNNYGGPMGANR (0) + 1 Oxidation (M) NRNFGGGNNNYGGPMGANR (1) + 1 Oxidation (M) | 239.23 |  |
| 172 | CG31049 | Darkener of apricot | 229251 | 2D | 3 peptides matched IIVPKNSK (1) YLKSLPDYSELDR (1) RGSTTSATGVEPPLMAAPPQPTK | 33.83 |  |
| 173 | CG10443 | Leukocyte-antigen-related-like | 182018 | 2D | 5 peptides matched LTPVNYK (0) TPGGVPVRPTVSLK (0) Multi-Hit IDHGLGSERNMTLR (1) MDSSKFVTANLPCNKHK (2) + 1 Oxidation (M) VTVRIKPEDVPLNLRAHDVSTHSMTLSWSPPIR (2) + 1 Oxidation (M) | 35.61 |  |
| 174 | CG10279 | Rm62 | 78547 | Both | 1D: 3 peptides matched GDGPIALVLAPTR (0) MLDMGFEPQIR (0) + 2 Oxidation (M) EANQEINPALENLAR (0)  2D: 1 peptides matched TLLSDIYDTSESPGK (0) | 1D: 61.41  2D: 25.8 | |
| 175 | CG18255 | Stretchin / myosin light chain kinase | 497012 | 2D | 6 peptides matched DETGEASTSCR (0) Multi-Hit ITTSAMLHVYETK (0) + 1 Oxidation (M) KPKPDEEEQTSSRR (1) SAPGQASELVQITNTPQRSTSSDASDR (1) LTATLDSNGYVELIIAEATVR (0) SKMVLQMEEQAK (1) | 35.5 |  |
| 176 | CG12819 | slender lobes | 158653 | 1D | 6 peptides matched LSLLETDSRPSTPQLDTVAELGTASPR (0) AENKILAR (1) ASLARGETPEPTTPSSVKR (2) SLSGAPEDLEEMEIKHERK (2) KIEENMDDVFETLRVESPK (2) + 1 Oxidation (M) GNKSVYEIMDSYETEDPK (1) | 36.54 |  |
| 177 | CG2216 | Ferritin 1 heavy chain | 23145 | 2D | 3 peptides matched GQLTEGVSDLINVPTVAK (0) LVEYLSMR (0) + 1 Oxidation (M) MMDTNGELGEFLFDKTL (1) + 2 Oxidation (M) | 110.58 |  |
| UNKNOWN | |  |  |  |  |  |  |
| 178 | CG1516 |  | 130863 | 1D | 2 peptides matched VGEEFDVPLER (0) GLPPVEAYLNIPELIR (0) | 34.91 |  |
| 179 | CG2213 |  | 28461 | 1D | 3 peptides matched DLSSVADYAELFK (0) LLTIAANMEQLNYLR (0) + 1 Oxidation (M) TLEEYPANLQK (0) | 129.29 |  |
| 180 | CG2852 |  | 22199 | 2D | 4 peptides matched DTNGSQFFITTK (0) IIKDFMIQGGDFTKGDGTGGR (2) + 1 Oxidation (M) ILSGMNVVR (0) DRPVKDVVIANSGTLPVSEAFSVAKADATD (2) | 134 |  |
| 181 | CG3221 |  | 62576 | Both | 1D: 4 peptides matched FLSGNITDANILTER (0) GIQFYALSYIR (0) LLSYSFQSMLNR (0) + 1 Oxidation (M) LLQIEAESPGLLNYK (0)  2D: 2 peptides matched GIQFYALSYIR (0) FLSGNITDANILTER (0) | 1D: 169.17  2D: 77.6 | |
| 182 | CG3226 |  | 25783 | 2D | 3 peptides matched DSELSKDGDNPESALVNIMKK (2) + 1 Oxidation (M) YLHELTDYGWDQSAK (0) DSELSKDGDNPESALVNIMK (1) + 1 Oxidation (M) | 81.57 |  |
| 183 | CG3229 / CG33123 | | 134872 | 1D | 2 peptides matched TFITTDANPYFDSFVR (0) VLAEVTGQDLLGVPLSAPLTK (0) | 42.26 |  |
| 184 | CG3339 |  | 521582 | 2D | 8 peptides matched VRIIEEDVSIKTK (2) Multi-Hit SVLVVAGTLKRDDHSRPEDQVLMR (2) RTVDEIEAKVR (2) WPLMIDPQLQGIK (0) VYHDRLVDPYDIKSFK (2) QFMDYRQWYDRQR (2) EWKDGLFSSIMREQANMPPGNPK (2) + 1 Oxidation (M) IIASKVDNSLAFQWQSQLRHR (2) Multi-Hit | 38.23 |  |
| 185 | CG3501 |  | 22780 | 2D | 4 peptides matched AAIQVFNCPGDSGAR (0) EGLVIAGYYAAPENFYDNQVDKTPAAK (1) YPHQAVNGLLLAEK (0) IADKIQENFK (1) | 126.75 |  |
| 186a | CG3678 |  | 32725 | 2D | 6 peptides matched LSQWALEQLLTK (0) QTIVPLEAAFGNLEIK (0) QLALEFPGSLR (0) ALYGIYLCCNHLDNSR (0) AAFCMEEVLLHNPHSHLIHQR (0) + 1 Oxidation (M) MSWSDVRDQFR (1) + 1 Oxidation (M) | 232.12 |  |
| 187 | CG3731 |  | 51874 | 2D | 5 peptides matched AVEILADIIQNSK (0) NIQSIGKADLTDYIQTHYK (1) LCTMVTEAEVER (0) + 1 Oxidation (M) NIQSIGKADLTDYIQTHYKASR (2) TLLNIPATQVTK (0) | 144.83 |  |
| 188 | CG3756 |  | 38137 | 2D | 7 peptides matched YPQLNDAVTLAR (0) FSPVATATYR (0) NVYRYPQLNDAVTLAR (1) DAGKDQSNFDDIYK (1) DQSNFDDIYK (0) NRKEPLLYMEEYR (2) + 1 Oxidation (M) DAGKDQSNFDDIYKNHK (2) | 224.77 |  |
| 189 | CG3861 |  | 51574 | 2D | 9 peptides matched HLPEDETFQLVSK (0) EMNYYTVLFGVSR (0) + 1 Oxidation (M) HLPEDETFQLVSKIYK (1) LPVVAATIYCNTYR (0) SGQVVPGYGHAVLR (0) ALVTETSVLDADEGIR (0) SFSTDLLVK (0) ALVTETSVLDADEGIRFR (1) MGETTIDMMYGGMR (0) + 4 Oxidation (M) | 356.19 |  |
| 190 | CG3950 |  | 355620 | 2D | 7 peptides matched KPSDTNGSPSIK (0) DSLVEETRITTTTTTTRQGR (2) Multi-Hit MRNADNVEEPGDSSEDR (1) + 1 Oxidation (M) KESAPVPRVTR (2) VSRFTHSAEKVK (2) QSPEKDLTNQQRR (2) Multi-Hit NPEDDVDGDSSSPDASPTR (0) | 36.92 |  |
| 191 | CG3957 |  | 36073 | 2D | 6 peptides matched FSPDGELYASGSEDGTLR (0) SVAFDRDSENIVTGSNEK (1) CIISAAEDKTVR (1) VPTNVASASLHPDK (0) VFNLEQPEAQPEEYAGHTGAIK (0) VFNLEQPEAQPEEYAGHTGAIKR (1) | 135.94 |  |
| 192 | CG4365 |  | 34183 | Both | 1D: 2 peptides matched TVQEQQLTLSK (0) SVATSVETQLTATYFR (0)  2D: 7 peptides matched SVATSVETQLTATYFR (0) ILPALQDNYMYLIVDTK (0) + 1 Oxidation (M) ASQDPTVPSTIGEEK (0) FFEGTPEEMYEALCTK (0) + 1 Oxidation (M) VFCGHEYTLQNMSFAR (0) + 1 Oxidation (M) SVATSVETQLTATYFRVQK (1) HVEPDNEVIQQRIEWAK (1) | 1D: 42.25  2D: 166.01 | |
| 193 | CG4389 |  | 84074 | 1D | 10 peptides matched NADIIIEAVFEDIKVK (1) VVITVGDGPGFYTTR (0) FSGGNLEVMNDLVLAGFLGR (0) + 1 Oxidation (M) GANAPEDLTLR (0) FGFPVGAATLADEVGIDVGSHIAVDLAK (0) TAVQVANDLASGK (0) ILSTMLSEAIR (0) + 1 Oxidation (M) ELEAVVPEHCVIATNTSAIPITK (0) LGLPEVMLGLLPGGGGTVR (0) + 1 Oxidation (M) VNSLGSEVSDEFER (0) | 430.82 |  |
| 194 | CG4865 |  | 21425 | 2D | 6 peptides matched VSDVNQVDQAAKDIASLR (1) EFAQNQEALR (0) LVEDLLPPRETNN (1) QQREFAQNQEALR (1) LGSDLRPAILDSNDVK (0) SKLGSDLRPAILDSNDVK (1) | 232.4 |  |
| 195 | CG5028 |  | 44431 | Both | 1D: 3 peptides matched LSDGLFLEVANR (0) YAFEFAR (0) HAVTMLPGGGIGPELMGYVR (0) + 2 Oxidation (M)  2D: 13 peptides matched TPDIGGTNSSTDVVENILK (0) LSDGLFLEVANR (0) VTGTDIPSAQYGGR (0) NYGDHYAIFEPGTR (0) TPDIGGTNSSTDVVENILKILSAK (1) HHDIDVVLIR (0) Multi-Hit YAFEFAR (0) RVNWPHGNYFSQI (1) HAVTMLPGGGIGPELMGYVR (0) + 2 Oxidation (M) Multi-Hit SYPGIPARHHDIDVVLIR (1) TPDIGGTNSSTDVVENILKILSAKR (2) ANIMKLSDGLFLEVANR (1) + 1 Oxidation (M) QNTDGEYAMLEHESVPGIVESMK (0) + 2 Oxidation (M) | 1D: 104.33  2D: 619.64 | |
| 196 | CG5214 |  | 49924 | 2D | 2 peptides matched TLIDALDAILPPARPTDK (0) EGNADGKTLIDALDAILPPARPTDK (1) | 39.65 |  |
| 197 | CG5384 |  | 53707 | 2D | 7 peptides matched AQLFALTGVQPDR (0) GTTVTPIVLLQALHR (0) Multi-Hit LPAYLTVQFVR (0) ESVPEVPATPVK (0) FIEDMNEAEAATAMR (0) + 2 Oxidation (M) ASPQFAQTGENGTYR (0) TALSTFSNDGTDTMSTAFSISSAMK (0) + 2 Oxidation (M) | 297.51 |  |
| 198 | CG5525 |  | 55372, 57116, 90238 | Both | 1D: 1 peptides matched AFADALEVIPSTLAENAGLNPIATVTELR (0)  2D: 7 peptides matched VVSQQSSLLAPIAVDAVLK (0) IGLIQFCISAPK (0) TDMDHNVIVSDYAAMDR (0) + 2 Oxidation (M) DAVSDLAQHFLDKIK (1) SILKIDDIVNTFS (1) QMSTPIELDDRETLIK (1) + 1 Oxidation (M) GLHPTAISDSFQR (0) | 1D: 65.38  2D: 181.81 | |
| 199 | CG5590 |  | 44354 | 2D | 8 peptides matched GAPVENEAAAEDAAAPASGGDVK (0) TLFITGASR (0) YDLMHNINTR (0) + 1 Oxidation (M) AYPCVVDVRDEQQVR (1) KPEIMADAAYAILTREPR (1) + 1 Oxidation (M) IESLLSPEIVSK (0) SNHAHILNISPPLSMKPK (0) + 1 Oxidation (M) GAPVENEAAAEDAAAPASGGDVKIPQLFR (1) | 173.99 |  |
| 200 | CG5787 |  | 100258 | 1D | 4 peptides matched LTTEQVVYAIIK (0) VAPPPPLETEAASK (0) TFLDNLTELNMVEIQDIIR (0) + 1 Oxidation (M) GGPQGGKPAGVPSRPPNAPGFPVQISQK (0) | 149.51 |  |
| 201 | CG5792 |  | 154831 |  | 4 peptides matched EWTKIYRTNNYK (2) TVDVCDIAINPK (0) Multi-Hit SNFIEGNDNFRDQNLR (1) DAAAIRKSR (2) | 35.6 |  |
| 202 | CG6311 |  | 38880 | 2D | 1 peptides matched GASDLAITLLGGAR (0) | 57.15 |  |
| 203 | CG6543 |  | 31582 | 2D | 10 peptides matched FGQPEIALGTIPGAGGTQR (0) TISAIVLTGSEK (0) VVPADQLLGEAVK (0) VVPADQLLGEAVKLGEK (1) Multi-Hit NVGVITLNRPK (0) ELSTALQQFSK (0) SKAMEMCLTGNMIGAQEAEKLGLASK (2) + 3 Oxidation (M) FGQPEIALGTIPGAGGTQRLTRVVGK (2) AMEMCLTGNMIGAQEAEKLGLASK (1) + 3 Oxidation (M) EGMTAFAEKRPAKFTNE (2) + 1 Oxidation (M) | 347.65 |  |
| 204 | CG6793 |  | 67553 | 2D | 1 peptides matched LQMLEEMEQVR (0) + 1 Oxidation (M) Multi-Hit | 34.33 |  |
| 205 | CG7033 |  | 58063 | 2D | 27 peptides matched VQDEEVGDGTTSVTVLASELLR (0) Multi-Hit ALLSLPTAIADNAGYDSAQLVSELR (0) Multi-Hit ILVDMSRVQDEEVGDGTTSVTVLASELLR (1) + 1 Oxidation (M) Multi-Hit LSSFIGAIAIGDLVK (0) NAGHVEVTNDGATILR (0) Multi-Hit KAAETPGKEAIAIEAFAR (2) QLIYNYPEQLFADAR (0) SLHDALCVLAATVKESR (1) VMSIEHADFDGIER (0) + 1 Oxidation (M) Multi-Hit TTLSSKILHQHKDFFANLAVDAVMR (2) Multi-Hit GATQQILDEADR (0) ELGITESFAVKR (1) LAYCTGGEIVSTFENPSLVK (0) Multi-Hit QTLGLDMELGK (0) + 1 Oxidation (M) LGECDVIEQVMIGEDTLLR (0) + 1 Oxidation (M) LSSFIGAIAIGDLVKSTLGPK (1) AAETPGKEAIAIEAFAR (1) IIFGGGCSEALMATAVLKK (1) + 1 Oxidation (M) QATQVALEALTAAAQDNSSSDEKFRNDLLNIAR (2) GATQQILDEADRSLHDALCVLAATVKESR (2) NAGHVEVTNDGATILRAVGVDNPAAK (1) Multi-Hit NDLLNIAR (0) ALLSLPTAIADNAGYDSAQLVSELRAGHAQGK (1) ILHQHKDFFANLAVDAVMR (1) + 1 Oxidation (M) QVLMSASEAAEMILR (0) + 1 Oxidation (M) Multi-Hit QVLMSASEAAEMILRVDNIIR (1) + 2 Oxidation (M) Multi-Hit ILIANTPMDTDKIK (1) + 1 Oxidation (M) | 1339.65 |  |
| 206 | CG7461 |  | 71326 | 1D | 9 peptides matched IFEGTNDILR (0) FFSDVNDAAR (0) NIVNEQILLTR (0) ASENESFMANIFR (0) + 1 Oxidation (M) ELTNSLIDPFER (0) GILLYGTPEQK (0) LANAAIDIYAMVVTQSR (0) + 1 Oxidation (M) ASNTAEVYFEDVKIPIENVLGK (1) AVNLNLPTAQHELNMTK (0) + 1 Oxidation (M) | 365.91 |  |
| 207 | CG7488 |  | 41674 | 2D | 3 peptides matched TLTNDTLTVGK (0) SLHIAVIGVPNVGK (0) Multi-Hit LLLDLIK (0) | 149.85 |  |
| 208 | CG7504 |  | 161600 | 1D | 4 peptides matched LELLSTIER (0) LILHNGTNLMGRHSR (1) RTLRISSSSDEDSADEVIVHQTK (2) EKKLQLISNQLSPPLTQR (2) | 47.72 |  |
| 209 | CG7834 |  | 27345 | 2D | 18 peptides matched QAGATVADVDALVAK (0) KLAEEVIAVSVGPAQSQEVIR (1) GVHVEIPAAEYELLQPIHVSK (0) TALAMGADRGVHVEIPAAEYELLQPIHVSK (1) + 1 Oxidation (M) IEVISVEDPPVR (0) EIDGGLETIKTKTPAVLSADLR (2) LALDEKADLVILGK (1) QAGATVADVDALVAKLK (1) GVHVEIPAAEYELLQPIHVSKILAK (1) TPAVLSADLR (0) YATLPNIMK (0) + 1 Oxidation (M) IEVISVEDPPVRQAGATVADVDALVAK (1) HSMNPFDEIAVEEAVKLK (1) + 1 Oxidation (M) QAGATVADVDALVAKLKEGGHI (2) VTAKDLGVDTSPRIEVISVEDPPVR (2) YATLPNIMKAK (1) + 1 Oxidation (M) TDAGLTITREIDGGLETIK (1) TPAVLSADLRLNTPR (1) | 597.22 |  |
| 210 | CG8036 |  | 68031 | 2D | 7 peptides matched TIPGSTIFYPSDAVSTER (0) HVVNAVNEILKD (1) VVVVEDHYQQGGLGEAVLSALAGER (0) TSRPNTCVIYDNEEPFTIGR (0) HLYVPTVPR (0) TGAAPDVDINNIK (0) TVQDLKDLAQK (1) | 375.33 |  |
| 211 | CG8142 |  | 39554 | 1D | 4 peptides matched NVDDVVEQSEVVAVLR (0) NVDDVVEQSEVVAVLRK (1) IIILDEADSMTHAAQSALR (0) + 1 Oxidation (M) EIGFSAYSVGQMMEQFVEFIVHHPGLNDPQK (0) + 2 Oxidation (M) | 119.2 |  |
| 212 | CG8231 |  | 58246 | Both | 1D: 4 peptides matched TLAVNSGYDAQDTIVK (0) AAQALAINISAAK (0) MLVSGAGDIKITK (1) + 1 Oxidation (M) Multi-Hit LAVQAFADALLVIPK (0)  2D: 17 peptides matched TLAVNSGYDAQDTIVK (0) AAQALAINISAAKGLQDVMR (1) ALVPGAGAFEVR (0) SVCDGTDKTFVLINQK (1) Multi-Hit GIDPISLDALAKEGILALR (1) Multi-Hit MLVSGAGDIKITK (1) + 1 Oxidation (M) Multi-Hit GIDPISLDALAK (0) DGNVLLHEMQIQHPTASMIAR (0) Multi-Hit LAVQAFADALLVIPK (0) AAQALAINISAAK (0) AINNTIADKALVPGAGAFEVR (1) GIDPISLDALAKEGILALRR (2) ASTAQDDSTGDGTTTTVMLIGELLK (0) + 1 Oxidation (M) Multi-Hit ALEVLDQVKVPVEINKK (2) LSPELVGLDLATGEPMKPVDLGVYDNYIVKK (1) + 1 Oxidation (M) AYNELVAFKDTIKGK (2) MLVSGAGDIKITKDGNVLLHEMQIQHPTASMIAR (2) + 3 Oxidation (M) | 1D: 146.77  2D: 760.99 | |
| 213 | CG8258 |  | 59433 | 1D | 2 peptides matched LGITTAEISDGYEK (0) FVEGDVTYAEK (0) | 66.91 |  |
| 214 | CG8351 |  | 59386 | 2D | 2D: 28 peptides matched EGTDSSQGKPQLVSNINACQSIVDAVR (0) SQDAEVGDGTTSVVLLAGEFLK (0) LPIGDVATQYFADR (0) QLCDNAGFDATNILNK (0) TIAGKEQLLIAAIAKGLEIIPR (2) TIAGKEQLLIAAIAK (1) INEMAVQIVEQSK (0) + 1 Oxidation (M) QLCDNAGFDATNILNKLR (1) INEMAVQIVEQSKDQQR (1) + 1 Oxidation (M) Multi-Hit KVTGGSLEESQLVSGVAFK (1) Multi-Hit TSTLILRGGAEQFLEETER (1) LIHQQKDFFSR (1) FNLFQGCPNAK (0) ACGGAVMTTANDIKPNVLGLCEHFEER (0) + 1 Oxidation (M) INALTAAAEAACMILSVDETIKSPK (1) + 1 Oxidation (M) Multi-Hit MQPQIVLLK (0) Multi-Hit MQPQIVLLKEGTDSSQGKPQLVSNINACQSIVDAVR (1) + 1 Oxidation (M) VVDAEWQILYNKLAK (1) EGTDSSQGKPQLVSNINACQSIVDAVRTTLGPR (1) VTGGSLEESQLVSGVAFK (0) QVGGERFNLFQGCPNAK (1) ALQLCMEKINEMAVQIVEQSKDQQR (2) + 2 Oxidation (M) VTGGSLEESQLVSGVAFKKTFSYAGFEMAPK (2) + 1 Oxidation (M) DNAEIRVDNVKEYQK (2) LLEIIHPAAK (0) SLHDAIMIVRR (1) INALTAAAEAACMILSVDETIK (0) + 1 Oxidation (M) TIKHDSVVAGGGAIEMELSK (1) + 1 Oxidation (M) | 2D: 1346.54 | |
| 215 | CG8507 |  | 44603 | 2D | 11 peptides matched AEISGFTAEELK (0) LNLVWAK (0) SFENDLNTLR (0) LVSSGPHSQDFIEPK (0) SLYMELK (0) + 1 Oxidation (M) LNRLWEKAEISGFTAEELK (2) ELESIKTELHHFESR (1) HRNKSLFK (2) LVSSGPHSQDFIEPKVQGLWR (1) EANDPHFQQVKQEKYDPDFK (2) SIQRPFRMAK (1) + 1 Oxidation (M) | 257.87 |  |
| 216 | CG8778 |  | 31909 | 2D | 8 peptides matched KGMTPEEATEFVKELR (2) + 1 Oxidation (M) Multi-Hit SLSPGIFCAGADLKER (1) Multi-Hit ILSPALAKELIFTAR (1) ELIFTAR (0) QGISVIGLNRPAAK (0) Multi-Hit LAIIPGAGGTQRLPR (1) GMVETFNDVLEDIKKDNGSR (2) + 1 Oxidation (M) NSFSRGMVETFNDVLEDIK (1) | 289.29 |  |
| 217 | CG8828 |  | 77978 | Both | 1D: 5 peptides matched SGSSLLLNQLQSR (0) ENLLIAQVAR (0) NLLDDMQAATQK (0) + 1 Oxidation (M) IQPVDYVR (0) YNNGSFADYESEFNLYYR (0)  2D: 8 peptides matched SGSSLLLNQLQSR (0) IYQFLLENPLR (0) QVYGDINSSINR (0) ENLLIAQVAR (0) IQPVDYVR (0) STFADIPNSLLLNVVMK (0) + 1 Oxidation (M) FCQDYSVFVDKLQSK (1) QHEEIDQLLDNLMAIGVK (0) + 1 Oxidation (M) | 1D: 168.05  2D: 304.93 | |
| 218 | CG9135 |  | 53459 | 2D | 4 peptides matched LPEDLLATLEK (0) YVASGPSAAHTILINMDR (0) + 1 Oxidation (M) ALGFGRNPSGQLGLSQDIK (1) YVASGPSAAHTILINMDRK (1) + 1 Oxidation (M) | 115.64 |  |
| 219 | CG9492 |  | 535381 | 2D | 11 peptides matched FYTLYK (0) Multi-Hit QIIIRVKLASCGFLENITLAR (2) TVIMKNFMK (1) + 1 Oxidation (M) LDIELPIVAATLMSR (0) DPKLYECPIYR (1) Multi-Hit AMQAEIVKNQVLIVKDK (2) ATHVTPKSYLNFIAGYKNIYQMK (2) QLPNKRTGYEYQTLFALTNQK (2) LKLAMDDLAGAEEQLR (1) + 1 Oxidation (M) TPFVCLLSIGSDPTTQIGALAK (0) EKLLPTPAKFHYVFSLR (2) | 39.43 |  |
| 220 | CG9547 |  | 45756 | 2D | 4 peptides matched DMLGANGISDEYHVIR (0) + 1 Oxidation (M) GYGCAGVSSVAYGLLTR (0) ASPTGMILMDEVR (0) + 2 Oxidation (M) QFGRPLAANQLIQK (0) | 117.32 |  |
| 221 | CG9615 |  | 36405 | 2D | 4 peptides matched DAGNVKIYGFSE (1) QYLLAYACDEK (0) TNPDQFATASGDKTVR (1) QYLLAYACDEKDNDR (1) | 162.86 |  |
| 222 | CG9945 |  | 58590 | 2D | 4 peptides matched IFVTKFNR (1) Multi-Hit IIGGSNNSTVIVTDIR (0) ATRLMSLESKIFVTK (2) + 1 Oxidation (M) YSPTGDKIIGGSNNSTVIVTDIRTR (2) | 33.46 |  |
| 223 | CG10077 |  | 88210 | 1D | 1Da: 2 peptides matched MLDMGFEPQIR (0) + 2 Oxidation (M) QVLMWSATWPK (0) + 1 Oxidation (M)  1Db: 3 peptides matched GDGPIALVLAPTR (0) MLDMGFEPQIR (0) + 2 Oxidation (M) NNNGPGATMNRNSFNGGSAGGPPR (1) + 1 Oxidation (M) | 1Da: 37.59  1Db: 66.42 | |
| 224 | CG10132 |  | 152356 | 2D | 1 peptides matched LSHVYSGLKSQLSEKNLIR (2) | 38.29 |  |
| 225 | CG10399 |  | 33770, 34173 | 2D | 3 peptides matched VVDSSVSGLGGCPYAK (0) NVNCTAAEAIER (0) GASGNAATEDVVYLLHGMGLDTGVNLDKLIQVGR (1) + 1 Oxidation (M) | 126.19 |  |
| 226 | CG10685 |  | 50147 | 2D | 1 peptides matched GALAELAGDEKNGEGSR (1) Multi-Hit | 35.9 |  |
| 227 | CG10932 |  | 43470 | 2D | 9 peptides matched TPIGSFQSQLAPLTATQLGAR (0) QQQDDFAIESYKR (1) IAEVVVVSAAR (0) SAAAWANKVFQDEIAPVK (1) QAAIFAGLPTNVCCTTVNK (0) QAAIFAGLPTNVCCTTVNKVCSSGMK (1) + 1 Oxidation (M) VNVHGGAVSIGHPIGMSGAR (0) + 1 Oxidation (M) Multi-Hit KLDVDPAKVNVHGGAVSIGHPIGMSGAR (2) + 1 Oxidation (M) RKPEIVISEDEEYKR (2) | 312.59 |  |
| 228 | CG11122 |  | 384998 | 2D | 9 peptides matched ISDCIAMLTGKLEEK (1) TGSRSSSSSSSR (1) NPGTGVVVPSSGPPAVSASAAVAAPVHPVK (0) Multi-Hit SSVVATGSSAAAVAAQQQR (0) Multi-Hit EEAAGLGGAAHSK (0) Multi-Hit TKAAMKGYENVNLQMDMLELAK (2) + 1 Oxidation (M) RALGR (1) QDKTPQPVDHQSPAAAVVAEK (1) NQPPPPAPAHVPPVVQAPPAPAPEVLTPPAR (0) | 35.79 |  |
| 123e* | CG11700 |  | 34335 | 1D | 2 peptides matched TITLEVEPSDTIENVK (0) TLSDYNIQKESTLHLVLR (1) | 61.34 |  |
| 229 | CG11876 |  | 39351 | 2D | 10 peptides matched AVETSLLAAAELAK (0) KGIEAEVINLR (1) TFYMSAGAVNVPIVFR (0) + 1 Oxidation (M) DALNSALDDELAR (0) GIEAEVINLR (0) VLSPYDAEDAR (0) SIRPLDTATIFASVR (0) DALNSALDDELARDDR (1) CAGVDVPMPYAK (0) Multi-Hit IMEDQTFFELDAPVWR (0) + 1 Oxidation (M) | 509.58 |  |
| 230 | CG11881 |  | 72826 | 1D | 2 peptides matched SLGLEAGTPNVSAK (0) SYLVVMPGGMR (0) + 2 Oxidation (M) | 65.9 |  |
| 231 | CG11905 |  | 65947 | 2D | 2 peptides matched EGGIIIQRLK (1) TTPKYHLFFLPRELALNEDGTPK (2) | 39.24 |  |
| 232 | CG11963 |  | 54910 | Both | 1D: 4 peptides matched VVYDPQTAEELSSK (0) DLNLNMPVVVR (0) + 1 Oxidation (M) DDLDKAADLAVHLAQIVK (1) EGGVDIEEVAASSPDAILYEPIDIGTGLTSEQAEK (0)  2D: 18 peptides matched LYGGEPANFLDVGGGATAEAVKAAFK (1) MDVNFEIPDAQK (0) + 1 Oxidation (M) Multi-Hit VVYDPQTAEELSSK (0) CDVIAEGIISATK (0) MIDQLLVTK (0) + 1 Oxidation (M) DLNLNMPVVVR (0) + 1 Oxidation (M) Multi-Hit LKTDNLVLKAQVLAGGR (2) AFNGPVLIASK (0) DDLDKAADLAVHLAQIVK (1) LRFDDNAEFR (1) LRFDDNAEFRQK (2) EANDIATKLKTDNLVLK (2) EMKMDVNFEIPDAQK (1) + 2 Oxidation (M) Multi-Hit REFYFAVMMER (1) + 2 Oxidation (M) EFYFAVMMER (0) + 2 Oxidation (M) VVYDPQTAEELSSKMIDQLLVTKQTGAAGR (2) + 1 Oxidation (M) VVYDPQTAEELSSKMIDQLLVTK (1) + 1 Oxidation (M) LYGGEPANFLDVGGGATAEAVK (0) | 1D: 88  2D: 678.80 | |
| 233 | CG12018 |  | 48008 | 2D | 6 peptides matched DISEENQLAPQPPR (0) ASAMAHVPTLQVAR (0) + 1 Oxidation (M) LDEALVVGTSGQNVSDLLR (0) IVLEDELQR (0) YENLSSIFR (0) TQSVAVVDLDTLDCR (0) | 337.95 |  |
| 234 | CG12140 |  | 65988 | 2D | 1 peptides matched AINEGGFQSLPQK (0) | 73.4 |  |
| 235 | CG12233 |  | 38584, 40844 | Both | 1D: 6 peptides matched VAEYAFQYAK (0) HMELNTYADKIER (1) + 1 Oxidation (M) MSDGLFLR (0) + 1 Oxidation (M) FGIPQAAIDSVNTNK (0) KVTLIPGDGIGPEISAAVQK (1) DLANPTALLLSAVMMLR (0) + 2 Oxidation (M)  2D: 20 peptides matched FGIPQAAIDSVNTNK (0) HMELNTYADKIER (1) + 1 Oxidation (M) Multi-Hit KVTLIPGDGIGPEISAAVQK (1) Multi-Hit CSEFTNEICAKL (1) MSDGLFLR (0) + 1 Oxidation (M) VAEYAFQYAKNNNR (1) Multi-Hit SLEGYKTLYDDVDVVTIR (1) IFTAANVPIEWEAVDVTPVRGPDGK (1) IGLKGPLMTPVGK (1) + 1 Oxidation (M) VTLIPGDGIGPEISAAVQK (0) TLYDDVDVVTIR (0) ENTEGEYSGIEHEIVDGVVQSIK (0) RVAEYAFQYAK (1) ANIMRMSDGLFLR (1) + 2 Oxidation (M) AAFETIKEGKYLTGDLGGR (2) YLDTVCLNMVQNPGK (0) + 1 Oxidation (M) VAEYAFQYAKNNNRK (2) IFTAANVPIEWEAVDVTPVR (0) DLANPTALLLSAVMMLR (0) + 2 Oxidation (M) CVRDMAQKFPEIQFEEK (2) + 1 Oxidation (M) | 1D: 208.64  2D: 661.93 | |
| 236 | CG12262 |  | 45871 | 2D | 13 peptides matched LLEEPLVAAYCVTEPGAGSDVSGIKTR (1) IYQIYEGTSQIQR (0) TRPPVAAGAVGLAQR (0) ENVLIGEGAGFK (0) AFTGFIVER (0) NSYYASIAK (0) AFTGFIVERDSPGLTPGRK (2) GITFEDVRVPK (1) KFTREEIIPVAAQYDK (2) AFTGFIVERDSPGLTPGR (1) LSAWEIDQGRR (1) LLEEPLVAAYCVTEPGAGSDVSGIK (0) IASDAVQIFGGNGFNSEYPVEK (0) Multi-Hit | 422.28 |  |
| 237 | CG12264 |  | 51074 | 2D | 1 peptides matched VLDAMLPYLTNFYGNPHSR (0) + 1 Oxidation (M) | 34.36 |  |
| 238 | CG12288 |  | 47937 | 1D | 3 peptides matched YQVKKLGAK (2) ENGSVKKEGAPTGQK (2) DAAAASAASKTSSKTK (2) | 41.01 |  |
| 239 | CG12512 |  | 65201 | 2D | 2 peptides matched GATMVLPAAGFSPK (0) + 1 Oxidation (M) YVIPIDAFPK (0) | 67.36 |  |
| 240 | CG13879 |  | 17525 | 1D | 3 peptides matched ILSSIEEATR (0) DFSIESVIEKVDSLEQR (1) LIPDIVVMQR (0) + 1 Oxidation (M) | 122.55 |  |
| 241 | CG13914 |  | 13776, 15668 | 2D | 5 peptides matched AFSQNVALSETQK (0) LVALYDTVGQMK (0) + 1 Oxidation (M) KLVALYDTVGQMK (1) + 1 Oxidation (M) EETYSALEGCLAYR (0) SNNTLLHIESNSK (0) | 170.79 |  |
| 242 | CG14100 |  | 45043 | 1D | 1 peptides matched VAPTPAPVIKDNELNLEFVR (1) | 42.8 |  |
| 243 | CG15100 |  | 112484 | 1D | 6 peptides matched NNSELLNNLGNFVNR (0) YKDLLVLPTLELDNGLR (1) GTDVELFQFMAK (0) + 1 Oxidation (M) SSDQLFIDLPK (0) KVFYVWFDAPFGYVSMTK (1) + 1 Oxidation (M) AGYIITESVEQLLCQK (0) | 78.33 |  |
| 244 | CG15356 |  | 108136 | 2D | 3 peptides matched KENDSGIDGKPNKK (2) Multi-Hit SGRRAQK (2) VTPLSSASTVNIIK (0) | 36.52 |  |
| 245 | CG15828 |  | 495242 | 2D | 7 peptides matched FFHGESLVYISGLLEK (0) VNEKNGIKQHDVDLSFETK (2) DPSQRLGLLAEYNSPGTK (1) VMQTLETFHSILEYAK (0) APTAALGVEVLADIKNLLNFDVK (1) EITGLFSVHLPR (0) ALDGMCAASKEIAPIPK (1) | 35.49 |  |
| 246 | CG16837 |  | 14815 | 2D | 2 peptides matched DLDPSNDITFMR (0) STPVVKKAVIR (2) | 40.84 |  |
| 247 | CG16935 |  | 33085 | 2D | 9 peptides matched VGLAEAATSTVNPTTAYR (0) EPVTVATGPLIFK (0) AWGINSVGIVR (0) KVGLAEAATSTVNPTTAYR (1) FVAPNHEMVPLAK (0) + 1 Oxidation (M) HLDNGGVLVTYGGMSR (0) + 1 Oxidation (M) ILAAPINPADINTIQGKYPVKPK (1) ILAAPINPADINTIQGK (0) FKDAAAAALSFK (1) | 330.35 |  |
| 248 | CG16969 |  | 25844 | 2D | 6 peptides matched RLDVGPNPSDEIKR (2) TTLQPIAELCDR (0) LVSIGSYAR (0) LDVGPNPSDEIKR (1) LQQAQAQLVQTKAELLR (1) AHKEVEAHLDELLAAKK (2) | 226.65 |  |
| 186b | CG17556 |  | 32744 | 2D | 6 peptides matched LSQWALEQLLTK (0) QTIVPLEAAFGNLEIK (0) QLALEFPGSLR (0) ALYGIYLCCNHLDNSR (0) AAFCMEEVLLHNPHSHLIHQR (0) + 1 Oxidation (M) MSWSDVRDQFR (1) + 1 Oxidation (M) | 232.12 |  |
| 249 | CG18190 |  | 26940 | Both | 1D: 3 peptides matched FFDSQAPGLENIK (0) LFQEAFNR (0) SLANAPLAKPIKPR (0)  2D: 8 peptides matched LVEDLVNDMINNNQLVELCKR (1) + 1 Oxidation (M) EDIMEASNQIYNK (0) + 1 Oxidation (M) FFDSQAPGLENIK (0) LFQEAFNR (0) LKLDKTVPIDR (2) KIEELCSGAAYCQMMEMIFPNCINLKR (2) + 3 Oxidation (M) EDIMEASNQIYNKLR (1) + 1 Oxidation (M) REDIMEASNQIYNKLR (2) + 1 Oxidation (M) | 1D: 131.35  2D: 369.26 | |
| 250 | CG30185 |  | 19979, 22261 | 2D | 3 peptides matched SLSPVDKEVYLNLSR (1) VQLNEEQVVTR (0) IANCLGVNPGKVQLNEEQVVTR (1) | 202.39 |  |
| 251 | CG31305 |  | 12817, 2D  14502,  17137 | | 2 peptides matched LGVEFDEETLDGR (0) SIITLDGNKLTQEQKGDKPTTIVR (2) | 92.54 |  |
| 252 | CG32005 |  | 77070 | 2D | 3 peptides matched KSIENLLEEVK (1) SNAIRGNVDNEITNQLK (1) KSIENLLEEVKR (2) | 44.25 |  |
| 253 | CG32026 |  | 77803 | 1D | 2 peptides matched MSDGLFLR (0) + 1 Oxidation (M) Multi-Hit SPAGGQGQKGGAGGK (1) | 57.93 |  |
| 254 | CG32094 |  | 89803 | 2D | 1 peptides matched IEIFDGR (0) | 39.6 |  |
| 255 | CG32113 |  | 442222 | 1D | 2 peptides matched IKNLAEDDR (1) LEQTSSIQFNCLNLAICVER (0) | 35.09 |  |
| 123d* | CG32744 |  | 60030 | 1D | 2 peptides matched TITLEVEPSDTIENVK (0) TLSDYNIQKESTLHLVLR (1) | 58.36 |  |
| 256 | CG33553 |  | 229251 | 2D | 3 peptides matched IIVPKNSK (1) YLKSLPDYSELDR (1) RGSTTSATGVEPPLMAAPPQPTK (1) + 1 Oxidation (M) | 33.83 |  |
| 257 | CG34001 |  | 16003 | 2D | 2 peptides matched LLETSMASQDVSMLNATTGAER (0) + 2 Oxidation (M) Multi-Hit SKTNEGERLLETSMASQDVSMLNATTGAER (2) + 2 Oxidation (M) | 46.6 |  |
