# Supplementary material for: A Microtubule Interactome: Complexes with Roles in Cell Cycle and Mitosis
Source: PLoS Biol. 2008 Apr 22;6(4):e98. doi: 10.1371/journal.pbio.0060098 (PMC2323305; doi:10.1371/journal.pbio.0060098)
Supplement: Table S2 — All 270 MAPs were classified into functional groups according to GO (Figure 2). Each number assigned relates to those numbers given in Table S1. Where a protein possesses more than one GO, the primary functional GO based on mutational analysis was used. Each GO code and associated descriptions are listed to show the justification for assignment of a particular functional group. (351 KB DOC) [file pbio.0060098.st002.doc]

Supplementary Table S2. **Table of 270 MAPs, classified in functional groups, according to gene ontology (GO).** All 270 MAPs were classified into functional groups according to GO (Figure 2). Each number assigned relates to those numbers given in Supplementary Table S1. Where a protein possesses more than one GO, the primary functional GO based on mutational analysis was used. Each GO code and associated descriptions are listed to show the justification for assignment of a particular functional group.

|  | MITOSIS/CELL CYCLE | |  |  |
| --- | --- | --- | --- | --- |
| 1 | CG1404 | Ran | GO:0007049 | cell cycle. |
| 2 | CG16983 | SkpA | GO:0007067 | mitosis. |
| 3 | CG17498 | Mad2 | GO:0007049 | cell cycle. |
| 4 | CG31196 | 14-3-3-ε | GO:0007088 | regulation of mitosis. |
| 5 | CG17870 | 14-3-3ζ | GO:0045448 | mitotic cell cycle, embryonic |
| 6 | CG8705 | Peanut | GO:0007067 | mitosis. |
| 7 | CG1403 | Septin 1 | GO:0007067 | mitosis. |
| 8 | CG4173 | Septin 2 | GO:0007067 | mitosis. |
| 9 | CG2916 | Septin 5 | GO:0007067 | mitosis. |
| 10 | CG3265 | EB1 | GO:0051227 | mitotic spindle assembly |
| 11 | CG5363 | cdc2 | GO:0007067 | mitosis. |
| 12 | CG4488 | wee1 | GO:0007049 | cell cycle. |
| 13 | CG10498 | cdc2c | GO:0007067 | mitosis. |
| 14 | CG6235 | twins / aar | GO:0007088 | regulation of mitosis. |
| 15 | CG7425 | meiotic 68 / UbcD1 | GO:0007067 | mitosis. |
| 16 | CG7581 | Bub3 | GO:0007093 | mitotic checkpoint |
| 17 | CG9193 | mus 209 | GO:0007052 | mitotic spindle organization and biogenesis. |
| 18 | CG9765 | D-TACC | GO:0007067 | mitosis. |
| 19 | CG4254 | twinstar | GO:0007067 | mitosis. |
| 20 | CG10212 | SMC2 | GO:0007067 | mitosis. |
| 21 | CG5000 | mini spindles | GO:0007067 | mitosis. |
| 22 | CG18242 | D-Titin | GO:0007076 | mitotic chromosome condensation |
| 23 | CG2210 | abnormal wing discs | GO:0007067 | mitosis. |
| 24 | CG3210 | Dynamin related protein 1 | GO:0000910 | cytokinesis |
| 25 | CG17566 | γ-tubulin at 37C | GO:0008275 | gamma-tubulin small complex. |
|  |  |  |  |  |
|  | CYTOSKELETON BINDING | |  |  |
| 26 | CG5020 | CLIP-190 | GO:0005875 | microtubule associated complex. |
| 27 | CG6384 | CP190 | GO:0005875 | microtubule associated complex. |
| 28 | CG5981 | Stathmin | GO:0005875 | microtubule associated complex. |
| 29a | CG4027 | Actin 5C | GO:0005884 | actin filament. |
| 29b | CG12051 | Actin 42A | GO:0005884 | actin filament. |
| 30 | CG10067 | Actin 57A | GO:0005884 | actin filament. |
| 31 | CG10540 | F-actin capping protein α | GO:0005875 | microtubule associated complex. |
| 32 | CG6174 | Actin-related protein 87C | GO:0005884 | actin filament. |
| 33 | CG54125 | myosin III | GO:0007010 | cytoskeleton organization and biogenesis |
| 34 | CG5336 | Ced-12 | GO:0007015 | actin filament organization. |
| 35 | CG6450 | lava lamp | GO:0008017 | microtubule binding. |
| 36 | CG10966 | receptor-degeneration A | GO:0007015 | actin filament organization |
| 39 | CG2331 | TER94 | GO:0000226 | microtubule cytoskeleton organization and biogenesis |
| 38 | CG11064 | retinoid-fatty acid binding protein (Rfabp) | GO:0008017 | microtubule binding. |
|  |  |  |  |  |
|  | MOTORS | |  |  |
| 39 | CG7507 | Dynein heavy chain 64C | GO:0007018 | microtubule-based movement. |
| 40 | CG18000 | Dynein intermediate chain (short wing) | GO:0007018 | microtubule-based movement. |
| 41 | CG10751 | Dynein light chain (roadblock) | GO:0007018 | microtubule-based movement. |
| 42 | CG7765 | Kinesin heavy chain | GO:0007018 | microtubule-based movement. |
| 43 | CG5433 | Kinesin light chain | GO:0007018 | microtubule-based movement. |
| 44 | CG8590 | Klp3A | GO:0007018 | microtubule-based movement. |
| 45 | CG6392 | CENP-meta | GO:0007018 | microtubule-based movement. |
| 46 | CG1453 | Klp10A | GO:0007018 | microtubule-based movement. |
| 47 | CG9191 | Klp61F | GO:0007018 | microtubule-based movement. |
| 48 | CG10923 | Klp67A | GO:0007018 | microtubule-based movement. |
| 49 | CG12298 | subito / KIF20A | GO:0007018 | microtubule-based movement. |
|  |  |  |  |  |
|  | VESICLE MEDIATED TRANSPORT | |  |  |
| 50 | CG9543 | ε COP | GO:0030126 | COPI vesicle coat |
| 51 | CG8014 | Receptor mediated endocytosis 8 | GO:0006897 | endocytosis |
| 52 | CG4422 | GDP dissociation inhibitor | GO:0016192 | vesicle-mediated transport. |
| 53 | CG8309 | Tango7 | GO:0007030 | Golgi organization and biogenesis |
| 54 | CG10686 | trailer hitch | GO:0006888 | ER to Golgi vesicle-mediated transport |
| 55 | CG8983 | ERp60 | GO:0005811 | lipid particle transport along microtubule |
| 56 | CG9057 | Lipid storage droplet-2 | GO:0031877 | lipid particle transport along microtubule |
| 57 | CG5474 | Signal sequence receptor β | GO:0016192 | vesicle-mediated transport. |
|  |  | |  |  |
|  | POLARITY | |  |  |
| 58 | CG12047 | Mud | GO:0016319 | mushroom body development |
| 59 | CG10545 | Gβ13F | GO:0045176 | apical protein localization |
| 60 | CG12021 | Patj | GO:0045176 | apical protein localization |
|  |  |  |  |  |
|  | DNA REPLICATION / REPAIR | |  |  |
| 61 | CG6258 | RfC 38kD subunit | GO:0006260 | DNA replication |
| 62 | CG14999 | RfC 40kD subunit | GO:0006260 | DNA replication |
| 63 | CG5313 | RfC3 | GO:0006260 | DNA replication |
| 64 | CG1119 | RfC140 | GO:0006260 | DNA replication |
| 65 | CG9633 | Replication protein A70 | GO:0005662 | DNA replication factor A complex |
| 66 | CG4978 | Mcm7 | GO:0003688 | DNA replication origin binding. |
| 67 | CG5949 | DNA polymerase δ | GO:0006281 | DNA repair. |
| 68 | CG6146 | Topoisomerase 1 | GO:0006260 | DNA replication |
| 69 | CG4003 | Pontin | GO:0006281 | DNA repair. |
| 70 | CG9750 | Reptin | GO:0006281 | DNA repair. |
| 71 | CG2905 | Nipped / Tra1 | GO:0006281 | DNA repair. |
| 72 | CG8169 | pms2 | GO:0006298 | mismatch repair |
|  |  |  |  |  |
|  | TRANSCRIPTION | |  |  |
| 73 | CG17228 | prospero | GO:0003700 | transcription factor activity |
| 74 | CG7008 | tudor sn | GO:0003713 | transcriptional coactivation |
| 75 | CG3644 | bicaudal | GO:0006357 | regulation of transcription from RNA polymerase II promoter. |
| 76 | CG4204 | Elongin B | GO:0003711 | transcriptional elongation regulator activity. |
| 77 | CG7885 | RNAP II 33kD subunit | GO:0009299 | mRNA transcription. |
| 78 | CG8651 | trithorax | GO:0006367 | transcription initiation from RNA polymerase |
| 79 | CG3696 | kismet | GO:0006357 | regulation of transcription from RNA polymerase II promoter. |
|  |  |  |  |  |
|  | TRANSLATION | |  |  |
| 80 | CG9946 | eIF2α | GO:0006413 | translational initiation. |
| 81 | CG4153 | eIF2β | GO:0006413 | translational initiation. |
| 82 | CG8882 | eIF3-S2 / Trip1 | GO:0006413 | translational initiation. |
| 83 | CG9075 | eIF4A | GO:0006413 | translational initiation. |
| 84 | CG4035 | eIF4E | GO:0006413 | translational initiation. |
| 85 | CG4429 | eIF4H / RNA-bp2 | GO:0003723 | RNA binding. |
| 86 | CG8280 | eEF1α 48D | GO:0003746 | translation elongation factor activity. |
| 87 | CG11901 | eEF1γ | GO:0003746 | translation elongation factor activity. |
| 88 | CG4912 | eEF1δ | GO:0003746 | translation elongation factor activity. |
| 89 | CG2238 | eEF2B | GO:0003746 | translation elongation factor activity. |
| 90 | CG12141 | Lysyl-tRNA synthetase | GO:0004824 | lysine-tRNA ligase activity |
| 91 | CG10506 | Glutaminyl-tRNA synthetase | GO:0004819 | glutamine-tRNA ligase activity |
| 92 | CG3821 | Aspartyl-tRNA synthetase | GO:0004815 | aspartate-tRNA ligase activity |
| 93 | CG14792 | Ribosomal protein L40 (Stubarista/Laminin R) | GO:0003735 | structural constituent of ribosome. |
| 94 | CG5119 | PABP / duo | GO:0045946 | positive regulation of translation |
| 95 | CG6779 | Ribosomal protein S3 | GO:0003735 | structural constituent of ribosome. |
| 96 | CG7014 | Ribosomal protein S5b | GO:0003735 | structural constituent of ribosome. |
| 97 | CG14206 | Ribosomal protein S10b | GO:0003735 | structural constituent of ribosome. |
| 98 | CG4916 | maternal expression at 31B | GO:0004004 | ATP-dependent RNA helicase activity |
| 99 | CG6137 | aubergine | GO:0006446 | regulation of translational initiation |
| 100 | CG11181 | cup | GO:0006446 | regulation of translational initiation |
|  |  |  |  |  |
|  | PROTEIN FOLDING | |  |  |
| 101 | CG10578 | Hsp-40 / DnaJ-like-1 | GO:0006457 | protein folding. |
| 102 | CG4183 | hsp-26 | GO:0006457 | protein folding. |
| 103 | CG8937 | Hsp-c1 | GO:0006457 | protein folding. |
| 104 | CG1579 | Hsp-c3 | GO:0006457 | protein folding. |
| 105 | CG4264 | Hsp-c4 | GO:0006457 | protein folding. |
| 106 | CG8542 | Hsp-c5 | GO:0006457 | protein folding. |
| 107 | CG4463 | Hsp-23 | GO:0006457 | protein folding. |
| 108 | CG4466 | Hsp-27 | GO:0006457 | protein folding. |
| 109 | CG12101 | Hsp-60 | GO:0006457 | protein folding. |
| 110a | CG5436 | Hsp-68 | GO:0006457 | protein folding. |
| 110b | CG31366 | Hsp-70Aa | GO:0006457 | protein folding. |
| 110c | CG18743 | Hsp-70Ab | GO:0006457 | protein folding. |
| 110d | CG31449 | Hsp-70Ba | GO:0006457 | protein folding. |
| 110e | CG31359 | Hsp-70Bb | GO:0006457 | protein folding. |
| 110f | CG6489 | Hsp-70Bc | GO:0006457 | protein folding. |
| 110g | CG5834 | Hsp-70Bbb | GO:0006457 | protein folding. |
| 111 | CG5374 | Tcp1-like | GO:0005832 | chaperonin-containing T-complex. |
| 112 | CG8439 | Cct5 | GO:0005832 | chaperonin-containing T-complex. |
| 113 | CG8977 | Cctγ | GO:0005832 | chaperonin-containing T-complex. |
|  |  |  |  |  |
|  | PROTEIN DEGRADATION | |  |  |
| 114 | CG1519 | Pros-α7 | GO:0006508 | proteolysis. |
| 115 | CG3422 | Pros-28.1 | GO:0005839 | proteasome core complex (sensu Eukaryota). |
| 116 | CG9327 | Pros 29 | GO:0005839 | proteasome core complex (sensu Eukaryota). |
| 117 | CG4904 | Pros-35 | GO:0005839 | proteasome core complex (sensu Eukaryota). |
| 118 | CG1489 | Pros-45 / Rpt6 | GO:0006508 | proteolysis. |
| 119 | CG3455 | Rpt4b | GO:0006508 | proteolysis. |
| 120 | CG2241 | Rpt6b | GO:0006508 | proteolysis. |
| 121 | CG10149 | Pros 44.5 / Rpn6 | GO:0006508 | proteolysis. |
| 122 | CG18174 | Rpn11 | GO:0006508 | proteolysis. |
| 123a | CG11624 | Ubiquitin p / Ubi63E | GO:0006512 | ubiquitin cycle. |
| 123b | CG2960 | Ubiquitin f / Ribosomal protein L40 | GO:0006512 | ubiquitin cycle. |
| 123c | CG5271 | Ubiquitin m / Ribosomal protein S27A | GO:0006512 | ubiquitin cycle. |
| 124 | CG4265 | Ubiquitin c-terminal hydrolase | GO:0006508 | proteolysis. |
| 125 | CG1548 | cathepsin D | GO:0006508 | proteolysis. |
| 126 | CG8947 | 26/29kD-proteinase | GO:0006508 | proteolysis. |
| 127a | CG7486 | caspase / dredd | GO:0006508 | proteolysis. |
|  |  |  |  |  |
|  | METABOLISM | |  |  |
| 127b | CG2140 | Cytochrome b5 | GO:0008202 | steroid metabolism |
| 128 | CG7113 | scully | GO:0006637 | acyl-CoA metabolism |
| 129 | CG8893 | GAP Dehydrogenase 2 | GO:0006096 | glycolysis. |
| 130 | CG14476 | Glucosidase II | GO:0005976 | polysaccharide metabolism |
| 131 | CG1633 | Thioredoxin Peroxidase 1 | GO:0006800 | oxygen and reactive oxygen species metabolism. |
| 132 | CG3593 | rudimentary-like | GO:0009116 | nucleoside metabolism |
| 133 | CG3481 | Alcohol Dehydrogenase | GO:0004022 | alcohol dehydrogenase activity |
| 134 | CG4581 | Thiolase | GO:0016507 | fatty acid beta-oxidation multienzyme complex |
| 135 | CG7660 | Dpxt | GO:0006800 | oxygen and reactive oxygen species metabolism. |
| 136 | CG9681 | PGRP-SB1 | GO:0009253 | peptidoglycan catabolism |
| 137 | CG11661 | Nc73EF | GO:0006096 | glycolysis |
| 138 | CG7642 | rosy | GO:0006800 | oxygen and reactive oxygen species metabolism. |
|  |  |  |  |  |
|  | NUCLEAR ENVELOPE | |  |  |
| 139 | CG4799 | Pendulin / Importin α2 | GO:0005643 | nuclear pore |
| 140 | CG2637 | fs(2)Ketel / Importin β | GO:0005643 | nuclear pore |
| 141 | CG1059 | Karyopherin β3 | GO:0005643 | nuclear pore |
| 142 | CG3820 | Nup214 / CAN | GO:0005643 | nuclear pore |
| 143 | CG11856 | Nup358 / RanBP2 | GO:0005643 | nuclear pore |
| 144 | CG9710 | nudC | GO:0007097 | nuclear migration. |
| 145 | CG6944 | Lamin | GO:0007097 | nuclear migration. |
|  |  |  |  |  |
|  | MITOCHONDRIA | |  |  |
| 146 | CG10691 | lethal (2) 37Cc | GO:0005739 | mitochondrion. |
| 147 | CG2151 | Thioredoxin Reductase-1 | GO:0005739 | mitochondrion. |
| 148 | CG3283 | Succinate Dehydrogenase | GO:0006099 | tricarboxylic acid cycle. |
| 149 | CG4169 | Ubiquinol-Cytochrome c Reductase | GO:0006122 | mitochondrial electron transport, ubiquinol to cytochrome c. |
| 150 | CG4600 | yippee interacting protein 2 | GO:0005739 | mitochondrion. |
| 151 | CG6439 | Isocitrate Dehydrogenase | GO:0005739 | mitochondrion. |
| 152 | CG6647 | Porin | GO:0006839 | mitochondrial transport. |
| 153 | CG7361 | Phosphodiesterase 9 | GO:0006122 | mitochondrial electron transport, ubiquinol to cytochrome c. |
| 154 | CG8470 | mitochondrial ribosomal protein S30 | GO:0005763 | mitochondrial small ribosomal subunit |
| 155 | CG2098 | Ferrochelatase | GO:0005743 | mitochondrial inner membrane |
| 156 | CG3612 | ATP synthase α / bellwether | GO:0015986 | ATP synthesis coupled proton transport |
| 157 | CG6030 | ATP synthase δ | GO:0015986 | ATP synthesis coupled proton transport |
|  |  |  |  |  |
|  | OTHER | |  |  |
| 158 | CG10844 | Ryanodine Receptor | GO:0006936 | muscle contraction |
| 159 | CG2985 | Yolk protein 1 | GO:0007296 | vitellogenesis |
| 160 | CG2979 | Yolk protein 2 | GO:0007296 | vitellogenesis |
| 161 | CG11129 | Yolk protein 3 | GO:0007296 | vitellogenesis |
| 162 | CG12357 | cap binding protein 20 | GO:0003723 | RNA binding |
| 163 | CG17437 | will die slowly | GO:0008283 | cell proliferation |
| 164 | CG5519 | Gbp | GO:0005525 | GTP binding |
| 165 | CG6226 | FK506 binding protein 1 | GO:0006457 | protein folding |
| 166 | CG7111 | RACK 1 | GO:0005080 | protein kinase C binding |
| 167 | CG7269 | Hel25E | GO:0006406 | mRNA export from nucleus |
| 168 | CG8472 | Calmodulin | GO:0006468 | protein amino acid phosphorylation |
| 169 | CG9748 | belle | GO:0004004 | ATP-dependent RNA helicase activity |
| 170 | CG9916 | Cyclophilin 1 | GO:0006457 | protein folding |
| 171 | CG6143 | Protein on ecdysone puffs | GO:0048024 | regulation of nuclear mRNA splicing |
| 172 | CG31049 | Darkener of apricot | GO:0004672 | protein kinase activity |
| 173 | CG10443 | Leukocyte-antigen-related-like | GO:0008045 | axon guidance |
| 174 | CG10279 | Rm62 | GO:0003723 | RNA binding |
| 175 | CG18255 | Stretchin / Myosin light chain kinase | GO:0004687 | myosin light chain kinase activity |
| 176 | CG12819 | slender lobes | GO:0007000 | nucleolus organisation and biogenesis |
| 177 | CG2216 | Ferritin 1 heavy chain | GO:0008043 | ferritin complex |
|  |  |  |  |  |
|  | UNCHARACTERISED | |  |  |
| 178 | CG1516 |  |  |  |
| 179 | CG2213 |  |  |  |
| 180 | CG2852 |  |  |  |
| 181 | CG3221 |  |  |  |
| 182 | CG3226 |  |  |  |
| 183 | CG3229 |  |  |  |
| 184 | CG3339 |  |  |  |
| 185 | CG3501 |  |  |  |
| 186a | CG3678 |  |  |  |
| 187 | CG3731 |  |  |  |
| 188 | CG3756 |  |  |  |
| 189 | CG3861 |  |  |  |
| 190 | CG3950 |  |  |  |
| 191 | CG3957 |  |  |  |
| 192 | CG4365 |  |  |  |
| 193 | CG4389 |  |  |  |
| 194 | CG4865 |  |  |  |
| 195 | CG5028 |  |  |  |
| 196 | CG5214 |  |  |  |
| 197 | CG5384 |  |  |  |
| 198 | CG5525 |  |  |  |
| 199 | CG5590 |  |  |  |
| 200 | CG5787 |  |  |  |
| 201 | CG5792 |  |  |  |
| 202 | CG6311 |  |  |  |
| 203 | CG6543 |  |  |  |
| 204 | CG6793 |  |  |  |
| 205 | CG7033 |  |  |  |
| 206 | CG7461 |  |  |  |
| 207 | CG7488 |  |  |  |
| 208 | CG7504 |  |  |  |
| 209 | CG7834 |  |  |  |
| 210 | CG8036 |  |  |  |
| 211 | CG8142 |  |  |  |
| 212 | CG8231 |  |  |  |
| 213 | CG8258 |  |  |  |
| 214 | CG8351 |  |  |  |
| 215 | CG8507 |  |  |  |
| 216 | CG8778 |  |  |  |
| 217 | CG8828 |  |  |  |
| 218 | CG9135 |  |  |  |
| 219 | CG9492 |  |  |  |
| 220 | CG9547 |  |  |  |
| 221 | CG9615 |  |  |  |
| 222 | CG9945 |  |  |  |
| 223 | CG10077 |  |  |  |
| 224 | CG10132 |  |  |  |
| 225 | CG10399 |  |  |  |
| 226 | CG10685 |  |  |  |
| 227 | CG10932 |  |  |  |
| 228 | CG11122 |  |  |  |
| 123e | CG11700 |  |  |  |
| 229 | CG11876 |  |  |  |
| 230 | CG11881 |  |  |  |
| 231 | CG11905 |  |  |  |
| 232 | CG11963 |  |  |  |
| 233 | CG12018 |  |  |  |
| 234 | CG12140 |  |  |  |
| 235 | CG12233 |  |  |  |
| 236 | CG12262 |  |  |  |
| 237 | CG12264 |  |  |  |
| 238 | CG12288 |  |  |  |
| 239 | CG12304 |  |  |  |
| 240 | CG13879 |  |  |  |
| 241 | CG13914 |  |  |  |
| 242 | CG14100 |  |  |  |
| 243 | CG15100 |  |  |  |
| 244 | CG15356 |  |  |  |
| 245 | CG15828 |  |  |  |
| 246 | CG16837 |  |  |  |
| 247 | CG16935 |  |  |  |
| 248 | CG16969 |  |  |  |
| 186b | CG17556 |  |  |  |
| 249 | CG18190 |  |  |  |
| 250 | CG30185 |  |  |  |
| 251 | CG31305 |  |  |  |
| 252 | CG32005 |  |  |  |
| 253 | CG32026 |  |  |  |
| 254 | CG32094 |  |  |  |
| 255 | CG32113 |  |  |  |
| 123d | CG32744 |  |  |  |
| 256 | CG33553 |  |  |  |
| 257 | CG34001 |  |  |  |
