# Supplementary material for: A Microtubule Interactome: Complexes with Roles in Cell Cycle and Mitosis
Source: PLoS Biol. 2008 Apr 22;6(4):e98. doi: 10.1371/journal.pbio.0060098 (PMC2323305; doi:10.1371/journal.pbio.0060098)
Supplement: Table S3 — The table includes all the primers used for dsRNA generation. CG numbers are indicated along side with a number that relates to those given in Table S1. More than one set of primers for genes are indicated when the phenotype was rechecked or when more than one transcript was known to exist. (139 KB DOC) [file pbio.0060098.st003.doc]

Supplementary Table S3. **List of primers used for generation of dsRNA.** The table includes all the primers used for dsRNA generation. CG numbers are indicated along side with a number that relates to those given in Supplementary Table S1. More than one set of primers for genes are indicated when the phenotype was rechecked or when more than one transcript was known to exist.

| ***Primers*** | ***Primers*** | ***Forward*** | ***Reverse*** |
| --- | --- | --- | --- |
|  | T7primerfordsRNAi | GAATTAATACGACTCACTATAGGGAGA | GAATTAATACGACTCACTATAGGGAGA |
|  | β-lactamase | CGACTCACTATAGGGAGATTCCTGTTTTTGCTCACC | CGACTCACTATAGGGAGAAGTGAGGCACCTATCTCA |
| 178 | CG1516 | CGACTCACTATAGGGAGACGGTCGCTCGATGAACTT | CGACTCACTATAGGGAGAATCGCCATCCGAGTGTTC |
| 179 | CG2213 | CGACTCACTATAGGGAGACCAGCTTTCGCTGGAAAC | CGACTCACTATAGGGAGAACGCGCAACATGTGAGAA |
| 180 | CG2852 | CGACTCACTATAGGGAGATTCCACCGCATCATCAAG | CGACTCACTATAGGGAGATGTTGGCGATGACCACAT |
| 181 | CG3221 | CGACTCACTATAGGGAGAAGGCCACGCAGTGTCTTA | CGACTCACTATAGGGAGATGGCAATCTCCTGCACAA |
| 182 | CG3226 | CGACTCACTATAGGGAGACCCAATCGACCGAAACAC | CGACTCACTATAGGGAGACCAAGCTTTGGCGATCAT |
| 183 | CG3229 | CGACTCACTATAGGGAGACAATCCCGTGCGAAACTT | CGACTCACTATAGGGAGATCTGCGGGTGCAGAAGAT |
| 184 | CG3339 | CGACTCACTATAGGGAGAAATGGCCAATCCGCACTA | CGACTCACTATAGGGAGAAGACCGCGCAACGTATAG |
| 185 | CG3501 | CGACTCACTATAGGGAGAGCCGAACGGGAAGGATTA | CGACTCACTATAGGGAGAGATCGTTGAGCGGCTGAT |
| 186a | CG3678 | CGACTCACTATAGGGAGAACGGCTCGCTTTGACATC | CGACTCACTATAGGGAGATGCCAAACTCGCCTTCAG |
| 187 | CG3731 | CGACTCACTATAGGGAGACGAGCGTTCGGTGATTCT | CGACTCACTATAGGGAGAAGGTTGGAGGCGTTGTTG |
|  | CG3731b | CGACTCACTATAGGGAGACGGACTCTGGGGCATTTA | CGACTCACTATAGGGAGAAGTCGGGCAGGTTCTCAA |
| 188 | CG3756 | CGACTCACTATAGGGAGAATACCGCACCGAGGAAAG | CGACTCACTATAGGGAGACCCGGCACTTTCTCTTCA |
| 189 | CG3861 | CGACTCACTATAGGGAGAAGCAGGAGCGCGTAAAGA | CGACTCACTATAGGGAGAAAAGTTCGCCGACCAATC |
| 190 | CG3950 | CGACTCACTATAGGGAGAATGGACCCAACCCCAAGT | CGACTCACTATAGGGAGAGGGGCAATCCTCGATCTT |
| 191 | CG3957 | CGACTCACTATAGGGAGAGCCACGCTGAACCGAAAT | CGACTCACTATAGGGAGAACGTTCGTCGGCACTTTC |
| 192 | CG4365 | CGACTCACTATAGGGAGATGACCACGCACCATCACT | CGACTCACTATAGGGAGATTCGTGAACGCGCATAAA |
| 193 | CG4389 | CGACTCACTATAGGGAGACCCTGTTCCGTGGACAAA | CGACTCACTATAGGGAGACGACCTTGCCTTGCTTGA |
| 194 | CG4865 | CGACTCACTATAGGGAGAGACCACACCGCCATCATT | CGACTCACTATAGGGAGATTGGTTCTGGGCGAACTC |
| 195 | CG5028 | CGACTCACTATAGGGAGACGGCATTGGTCCTGAACT | CGACTCACTATAGGGAGATGGCAACCTCCAGGAAGA |
| 196 | CG5214 | CGACTCACTATAGGGAGATTGCAAGGTGGGTGACTC | CGACTCACTATAGGGAGAGCATGCGGTTCATCTTGA |
| 197 | CG5384 | CGACTCACTATAGGGAGAGGGACGCGATGCAAAGTA | CGACTCACTATAGGGAGAATAGGCGCAATGCCAGTC |
| 198 | CG5525 | CGACTCACTATAGGGAGAATCGCTTCGCTGGATCAC | CGACTCACTATAGGGAGACGTTCTCCGCGAAAATGT |
| 199 | CG5590 | CGACTCACTATAGGGAGATGCTGACGGGTCCAGATT | CGACTCACTATAGGGAGACTTCAGCTTGCCGGTCAT |
| 200 | CG5787 | CGACTCACTATAGGGAGAGCCAAAGTTGCCGTGATT | CGACTCACTATAGGGAGACAGCGTTTCGGCGATAAT |
| 201 | CG5792 | CGACTCACTATAGGGAGAGTTGCGCACACGATGAGT | CGACTCACTATAGGGAGATCACCGAGGAGGAACTGA |
| 202 | CG6311 | CGACTCACTATAGGGAGAGCGAGGCAACTGGTTTGT | CGACTCACTATAGGGAGACCGCCTTATCGGCACTTA |
| 203 | CG6543 | CGACTCACTATAGGGAGATCTGCAATGGCCTGATGA | CGACTCACTATAGGGAGATCTTCTCGCCCAGCTTGA |
| 204 | CG6793 | CGACTCACTATAGGGAGATGGCCTCCTTGGTCATGT | CGACTCACTATAGGGAGATTGGGCCTCCCTGTTGTA |
| 205 | CG7033 | CGACTCACTATAGGGAGATTCAGCCCAACTGGTTTC | CGACTCACTATAGGGAGAGGTTTGATCGCAGCACTC |
| 206 | CG7461 | CGACTCACTATAGGGAGAATTCGAGGGCACCAATGA | CGACTCACTATAGGGAGATGGCGATGGTGGAAATCT |
| 207 | CG7488 | CGACTCACTATAGGGAGAGTGCCCAATGTGGGAAAG | CGACTCACTATAGGGAGAGGTCAAGGCGGAAACAAG |
| 208 | CG7504 | CGACTCACTATAGGGAGACGGAAATGCTGCCAAAAC | CGACTCACTATAGGGAGATTCGCTGAATGCGATCAA |
| 209 | CG7834 | CGACTCACTATAGGGAGAGCGTGAAGCACTCGATGA | CGACTCACTATAGGGAGATGGCCGTAACCTTCTTCA |
| 210 | CG8036 | CGACTCACTATAGGGAGACCGACGTGGACATCAACA | CGACTCACTATAGGGAGAACGGGACGTACGGATGAA |
| 211 | CG8142 | CGACTCACTATAGGGAGAGAGCGTGGCATCAATGTG | CGACTCACTATAGGGAGAAGGCGGAGAAGCCAATCT |
| 212 | CG8231 | CGACTCACTATAGGGAGATCGACCAGCGTGTGAAGA | CGACTCACTATAGGGAGACCAGCGTCTTGGGAATGA |
| 213 | CG8258 | CGACTCACTATAGGGAGAGCCAAATGGCATGAACAA | CGACTCACTATAGGGAGAACGGGGCAGGAGAAAATC |
| 214 | CG8351 | CGACTCACTATAGGGAGAATGGCCGTCCAAATTGTC | CGACTCACTATAGGGAGACGGTCGGCGAAATACTGT |
| 215 | CG8507 | CGACTCACTATAGGGAGATCGCAGCACAAGGACAAA | CGACTCACTATAGGGAGAGTTTTGCGCATGGGTTTT |
| 216 | CG8778 | CGACTCACTATAGGGAGAGGAAGCCACCGAATTTGT | CGACTCACTATAGGGAGAACCTGCATGCCCTTGTCA |
| 217 | CG8828 | CGACTCACTATAGGGAGAGAGGCCAAGCAGCGATTA | CGACTCACTATAGGGAGATGGGACTTGGCGTTTTGT |
| 218 | CG9135 | CGACTCACTATAGGGAGACGGAGAAGCCCACCATTA | CGACTCACTATAGGGAGAGCCGCAAGCAAAGTCAAC |
| 219 | CG9492 | CGACTCACTATAGGGAGAGCTACGGCGCTGATCAAT | CGACTCACTATAGGGAGACATAAAGCCCGGCATCAC |
| 220 | CG9547 | CGACTCACTATAGGGAGACCTGGTGGATCGCAAGAT | CGACTCACTATAGGGAGATAGCAAATGCCGCCAGTC |
| 221 | CG9615 | CGACTCACTATAGGGAGATGCCGATGGTCGTTACCT | CGACTCACTATAGGGAGACGTGGGTCCGAACTCAAT |
| 222 | CG9945 | CGACTCACTATAGGGAGACCGACATTCGCACAAGAA | CGACTCACTATAGGGAGATGCTCGGCTGCCTTATGT |
| 223 | CG10077 | CGACTCACTATAGGGAGATGGCCACCAGTATCGAGT | CGACTCACTATAGGGAGATCAGCTACCTGGCCCAAA |
| 224 | CG10132 | CGACTCACTATAGGGAGAATGTGGCCAATGCCAATC | CGACTCACTATAGGGAGATCCAGGAGCAAACGCTCT |
| 225 | CG10399 | CGACTCACTATAGGGAGAGCGAATTGTGGAGGTTGG | CGACTCACTATAGGGAGAGGGCTTCCACCACTTTGA |
| 226 | CG10685 | CGACTCACTATAGGGAGAACGGCGAAGGATCTCGTA | CGACTCACTATAGGGAGACTCGGCCATCTCCTTTTC |
| 227 | CG10932 | CGACTCACTATAGGGAGACTATGGCGGCGTCAATCT | CGACTCACTATAGGGAGAACGTCTTCCTTGCGAACG |
| 228 | CG11122 | CGACTCACTATAGGGAGACCACCGCTTTGGTTCATC | CGACTCACTATAGGGAGACGGCTCCATTTCCATGTC |
| 123e | CG11700 | CGACTCACTATAGGGAGACGGAAAGCACCTGGAGAA | CGACTCACTATAGGGAGATTCCGGCGAAAATCAAAC |
| 229 | CG11876 | CGACTCACTATAGGGAGAATACTCGATTGCGCCAAG | CGACTCACTATAGGGAGATGCTGCTCCGCACTGAAT |
| 230 | CG11881 | CGACTCACTATAGGGAGATCGGTGGGTTGGGTAATG | CGACTCACTATAGGGAGATTCAAACGCCGCTTCTTC |
| 231 | CG11905A | CGACTCACTATAGGGAGACCTTCGCCTTGCCATAGA | CGACTCACTATAGGGAGATGCCACCGATCCTTGATT |
|  | CG11905B | CGACTCACTATAGGGAGAGGCAATCGCCTTCTTCAA | CGACTCACTATAGGGAGAAATCTGCCCCTGGCCTAT |
|  | CG11905C | CGACTCACTATAGGGAGACCTGCCTGGCTGCATAAT | CGACTCACTATAGGGAGAACAATTCTCGCCGGATGA |
| 232 | CG11963 | CGACTCACTATAGGGAGAAACGCCGAGTTTCGTCAG | CGACTCACTATAGGGAGAACAACCACGGGCATGTTC |
| 233 | CG12018 | CGACTCACTATAGGGAGACCGCCTGGAGACCAAATA | CGACTCACTATAGGGAGATTTTAATGGGGGCCAGTG |
| 234 | CG12140 | CGACTCACTATAGGGAGAAATCCGCGTCTGCGTAGT | CGACTCACTATAGGGAGATTCGCTGCCCTCGTTAAG |
| 235 | CG12233 | CGACTCACTATAGGGAGAGCGTCGTTCAGAGCATCA | CGACTCACTATAGGGAGAAACAGGGCAGTGGGATTG |
| 236 | CG12262 | CGACTCACTATAGGGAGACTTGTCGCCGCCTATTGT | CGACTCACTATAGGGAGATCAGCCAGCATGAACTGC |
| 237 | CG12264 | CGACTCACTATAGGGAGATACGGCCCAAAGGGTGTA | CGACTCACTATAGGGAGAATGCTGCGACCATTGGAT |
| 238 | CG12288 | CGACTCACTATAGGGAGAAGAGGCGTCCACCGTTTT | CGACTCACTATAGGGAGATCCTTTGCAGCCCTTGTC |
| 239 | CG12304 | CGACTCACTATAGGGAGAAAATGCCTGGCGCAATCT | CGACTCACTATAGGGAGACAGCTGGCATGCGTATCA |
| 240 | CG13879 | CGACTCACTATAGGGAGATGACAAATGCGCCCAATA | CGACTCACTATAGGGAGAGCTGCTCCAGCGAATCTA |
| 241 | CG13914 | CGACTCACTATAGGGAGAAATGGAGCGGTCGAACAA | CGACTCACTATAGGGAGAATCCTCGCTGGCATACTG |
| 242 | CG14100 | CGACTCACTATAGGGAGAAGGTGCCATTTGCCTCAG | CGACTCACTATAGGGAGATGGGCTCCCTGATGTTGT |
| 243 | CG15100 | CGACTCACTATAGGGAGAATCCTGGTTGCGGCTATG | CGACTCACTATAGGGAGACTCGGTTGCCATGATGTG |
| 244 | CG15356 | CGACTCACTATAGGGAGATGAGGGAAGCGGTGTGAT | CGACTCACTATAGGGAGACGCCCACCAAGAAGTTTG |
| 245 | CG15828 | CGACTCACTATAGGGAGATGGCTCTGCTCGATGTGA | CGACTCACTATAGGGAGATGCCTGCCACAGTTCAGA |
| 246 | CG16837 | CGACTCACTATAGGGAGAGGGATGCGTTCGTCAATC | CGACTCACTATAGGGAGAGAGCGAATCCGCATGAAG |
| 247 | CG16935 | CGACTCACTATAGGGAGACGGTGAAGCCCAAGTTTC | CGACTCACTATAGGGAGATTTTCCGCCCACACAGTT |
| 248 | CG16969 | CGACTCACTATAGGGAGACCCGAACACTCGGAAGAT | CGACTCACTATAGGGAGATTTCAATTCGGCGGTCTC |
| 186b | CG17556 | CGACTCACTATAGGGAGAAACGAGCGGCATCTCATC | CGACTCACTATAGGGAGACTGCCAAACGCTGGTGTA |
| 249 | CG18190 | CGACTCACTATAGGGAGATGCTGCCTACTGCCAGAT | CGACTCACTATAGGGAGAGATTGCTGGCCTCCATGA |
| 250 | CG30185 | CGACTCACTATAGGGAGAGCACGAGTGGGCAAAAGA | CGACTCACTATAGGGAGACGGCGAAAGGGATTTCTG |
| 251 | CG31305** | CGACTCACTATAGGGAGACGGGACCTTCGTTCTTCA | CGACTCACTATAGGGAGAAGGAGCTGGCGTATGTGA |
|  | CG31305* | CGACTCACTATAGGGAGAACAGCGGAACGGAATCTG | CGACTCACTATAGGGAGATCCTGCGTCAGCTTGTTG |
| 252 | CG32005 | CGACTCACTATAGGGAGAGCAAACCTTTCGCCACAA | CGACTCACTATAGGGAGATCCGGCATTTCCCTGTAA |
| 253 | CG32026 | CGACTCACTATAGGGAGACGGGACCAGGGAAAAAGT | CGACTCACTATAGGGAGACGAGGCATTCCGAGTGAT |
| 254 | CG32094 | CGACTCACTATAGGGAGAGCTATTCCGGCGTTGTGT | CGACTCACTATAGGGAGAGCAGGAGAAGGGCAATGA |
| 255 | CG32113 | CGACTCACTATAGGGAGATCGCAAAGTGCTGCCATA | CGACTCACTATAGGGAGATGATTGCGTTCGAGTCCA |
| 123d | CG32744 | CGACTCACTATAGGGAGAGGCCAAGATCCAGGACAA | CGACTCACTATAGGGAGAGACGCAGGACCAAGTGAA |
| 256 | CG33553 | CGACTCACTATAGGGAGACTTCCAAACGCACGACAA | CGACTCACTATAGGGAGACGAAAGGGGCTGCTTGTT |
| 257 | CG34001 | CGACTCACTATAGGGAGAAGGCGATTGGGCCATTAT | CGACTCACTATAGGGAGATTGGGTCGCGATTTGAAG |
|  | CG16983 (SkpA) | CGACTCACTATAGGGAGACCGTTGCCCAATGTGAAT | CGACTCACTATAGGGAGATGCCGCGCTTTACTTCTC |
|  |  |  |  |
| Notes: |  |  |  |
| * | recognises both CG6783 and CG31305 | |  |
| ** | specific to CG6782 | |  |
| b | new pair of primers for confirmation | |  |
| A,B,C | primers for same gene but different regions | |  |
