# Supplementary material for: A Microtubule Interactome: Complexes with Roles in Cell Cycle and Mitosis
Source: PLoS Biol. 2008 Apr 22;6(4):e98. doi: 10.1371/journal.pbio.0060098 (PMC2323305; doi:10.1371/journal.pbio.0060098)
Supplement: Table S4 — The initial RNAi data collection for previously uncharacterised MAPs showing a phenotype based on the categories stated. Only the data for strong hits and hits are shown. All comparisons were made in relation to the negative control (β-lactamase dsRNA) present in a particular experiment. Each RNAi experiment was subsequently repeated for both strong hits and hits, in order to confirm the phenotype (data not shown). (255 KB DOC) [file pbio.0060098.st004.doc]

| ***Mitosis Analysis*** | | | | | | | | ***Abnormal spindle classification*** | | | | | | ***Chromosome problems in normal bipolar spindles*** | | |  |
| --- | --- | --- | --- | --- | --- | --- | --- | --- | --- | --- | --- | --- | --- | --- | --- | --- | --- |
| ***Strong hits*** | |  |  |  | |  |  |  |  |  |  |  |  |  |  |  |  |
| ***Gene*** | ***Total cells*** | ***Mitotic cells*** | ***Mitotic***  ***Index*** | ***Mitotic***  ***cells*** | | ***Normal mitotic cells*** | ***% Abnor mitotic cells*** | ***Mono***  ***polar*** | ***Bipolar*** | | ***Trip*** | ***Multi***  ***polar*** | ***Abnol*** | ***Lag*** | ***Align*** | ***Bridge*** | ***Comments*** |
|  | |  |  | | |  |  | | no aster | mono aster |  |  |  | |  |  | |
| control | 503 | 11 | 2.2% | 60 | | 44 | 26.7% | 3 |  | 3 | 6 | 3 | 1 | 2 | 1 |  |  |
| CG13879 | 509 | 28 | 5.5% | 66 | | 23 | 65.2% | 34 |  |  |  | 1 | 8 |  |  |  |  |
| CG16969 | 505 | 16 | 3.2% | 61 | | 28 | 54.1% | 33 |  |  |  |  |  |  |  |  |  |
|  | |  |  | | |  |  | |  |  |  |  |  | |  |  | |
| control | 524 | 11 | 2.1% | 62 | | 50 | 19.4% | 1 |  | 1 | 3 | 4 | 3 |  |  |  |  |
| CG3221 | 520 | 18 | 3.5% | 60 | | 36 | 40.0% | 18 |  |  | 5 |  | 1 |  |  | 3 |  |
| CG3731 | 504 | 6 | 1.2% | 51 | | 42 | 17.6% | 2 |  | 3 |  | 3 | 1 | 1 | 2 |  |  |
| CG4865 | 503 | 16 | 3.2% | 61 | | 34 | 44.3% | 13 |  |  | 1 |  | 13* |  | 1 |  | *low spindle density |
|  | |  |  | | |  |  | |  |  |  |  |  | |  |  | |
| control | 511 | 12 | 2.3% | 62 | | 53 | 14.5% | 2 |  | 5 | 2 |  |  | 1 |  |  |  |
| CG8351 | 516 | 38 | 7.4% | 61 | |  | 100.0% | 56 |  |  | 2 |  | 3 |  |  |  |  |
|  | |  |  | | |  |  | |  |  |  |  |  | |  |  | |
| control | 546 | 11 | 2.0% | 63 | | 53 | 15.9% | 1 | 1 | 2 | 6 |  |  | 1 | 1 | 1 |  |
| CG8828 | 538 | 19 | 3.5% | 60 | | 11 | 81.7% | 21 |  | 1 | 1 |  | 26* | 2 | 3 |  | *low spindle density |
| CG7033 | 540 | 17 | 3.1% | 63 | | 16 | 74.6% | 43 |  | 1 | 1 | 1 | 1 |  |  |  |  |
|  | |  |  | | |  |  | |  |  |  |  |  | |  |  | |
| control | 508 | 10 | 2.0% | 61 | | 48 | 21.3% | 1 |  | 2 | 5 | 1 | 4 | 1 | 1 | 2 |  |
| CG8258 | 515 | 21 | 4.1% | 60 | | 6 | 90.0% | 51 | 1 | 1 | 1 |  |  |  |  |  |  |
| CG8231 | 514 | 25 | 4.9% | 61 | | 11 | 82.0% | 49 |  |  | 1 |  |  |  |  |  |  |
|  | |  |  | | |  |  | |  |  |  |  |  | |  |  | |
| control | 518 | 14 | 2.7% | 60 | | 46 | 23.3% | 2 | 1 | 5 | 3 | 1 | 3 |  |  |  |  |
| CG5525 | 537 | 23 | 4.3% | 61 | | 15 | 75.4% | 42 |  |  | 1 |  | 3 |  | 1 |  |  |
| CG13914 | 510 | 15 | 2.9% | 64 | | 32 | 50.0% | 9 |  | 2 | 1 |  | 20* |  | 3 |  | *low spindle density |
|  | |  |  | | |  |  | |  |  |  |  |  | |  |  |  |
| control | 531 | 12 | 2.3% | 61 | | 50 | 18.0% | 2 | 1 | 3 | 2 | 3 |  | 2 | 2 |  |
| CG11881 | 520 | 27 | 5.2% | 62 | | 30 | 51.6% | 14 |  | 2 |  |  | 16* |  | 4 |  | *low spindle density |
| CG2213 | 517 | 17 | 3.3% | 60 | | 28 | 53.3% | 15 | 1 | 1 | 3 |  | 12* |  | 2 |  | *low spindle density |
| CG11700 | 520 | 2 | 0.4% | 60 | | 28 | 53.3% | 3 | 3 | 4 | 12 | 6 | 4 |  |  |  |  |
|  | |  |  | | |  |  | |  |  |  |  |  | |  |  | |
| ***hits*** | |  |  | | | |  | |  |  |  |  |  | |  |  | |
| control | 531 | 12 | 2.3% | 61 50 | | | 18.0% | 2 | 1 | 3 | 2 | 3 |  | 2 | 2 |  |  |
| CG11963 | 506 | 13 | 2.6% | 61 | 51 | | 16.4% |  |  | 1 | 8 | 8 | 3 |  | 3 | 1 |  |
|  | |  |  | |  | |  | |  |  |  |  |  | |  |  | |
| control | 505 | 10 | 2.0% | 63 | 46 | | 27.0% |  | 2 | 1 | 9 | 4 | 1 | 2 |  |  |  |
| CG8036 | 513 | 11 | 2.1% | 60 | 35 | | 41.7% | 1 | 1 | 3 | 17 | 3 |  |  | 2 |  |  |
| CG12018 | 503 | 8 | 1.6% | 60 | 40 | | 33.3% | 1 | 1 | 10 | 6 | 2 |  |  |  |  |  |
| CG8142 | 510 | 7 | 1.4% | 55 | 36 | | 34.5% |  | 1 | 4 | 10 | 3 | 1 | 3 | 7 | 1 |  |
| CG11876 | 511 | 10 | 2.0% | 61 | 40 | | 34.4% | 1 |  | 5 | 10 | 4 | 1 |  | 7 | 2 |  |
|  | |  |  | |  | |  | |  |  |  |  |  | |  |  | |
| control | 505 | 11 | 2.2% | 60 | 47 | | 21.7% |  |  | 2 | 9 |  | 2 | 1 | 2 | 1 |  |
| CG10932 | 527 | 9 | 1.7% | 60 | 40 | | 33.3% |  |  | 9 | 8 | 1 | 2 |  | 3 | 2 |  |

Supplementary Table S4**. Raw data of scored RNAi phenotypes for Strong Hits and Hits.** The initial RNAi data collection for MAPs showing a phenotype, based on the categories stated. Only the data for strong hits and hits are shown. All comparisons were made in relation to the negative control (β-lactamase dsRNA) present in a particular experiment. Each RNAi experiment was subsequently repeated for both strong hits and hits, in order to confirm the phenotype (data not shown).

| ***Interphase analysis*** | |  |  | ***Abnormal classification*** | | | | ***Binucleation analysis*** | | |  |
| --- | --- | --- | --- | --- | --- | --- | --- | --- | --- | --- | --- |
| ***Strong hits*** |  |  |  |  |  |  |  |  |  |  |  |
| ***Gene*** | ***Total cells*** | ***Normal*** | ***% abnormal*** | **Bundling** | **Compact** | **Curvy** | ***Abnormal*** | ***Total cells*** | ***Binucleated cells*** | ***% Binucleated cells*** | ***Comments*** |
| control | 101 | 84 | 16.8% | 5 | 5 | 6 |  | 105 | 6 | 5.7% |  |
| CG13879 | 111 | 78 | 29.7% | 1 | 26 | 6 |  | 105 | 6 | 5.7% |  |
| CG16969 | 102 | 79 | 22.5% |  | 23 |  |  | 109 | 5 | 4.6% |  |
|  | |  |  | |  |  |  | |  |  | |
| control | 210 | 195 | 7.1% |  | 8 | 5 |  | 200 | 8 | 4.0% |  |
| CG3221 | 209 | 188 | 10.0% | 1 | 4 | 15 |  | 206 | 12 | 5.8% |  |
| CG3731 | 201 | 165 | 17.9% |  | 2 | 34 |  | 207 | 6 | 2.9% |  |
| CG4865 | 220 | 194 | 11.8% |  | 17 | 8 |  | 202 | 7 | 3.5% |  |
|  | |  |  | |  |  |  | |  |  | |
| control | 207 | 201 | 2.9% | 1 | 1 | 4 |  | 210 | 7 | 3.3% |  |
| CG8351 | 204 | 66 | 67.6% | 1 |  | 26 | 111* | 214 | 7 | 3.3% | *low density |
|  | |  |  | |  |  |  | |  |  | |
| control | 204 | 193 | 5.4% |  | 4 | 7 |  | 216 | 6 | 2.8% |  |
| CG8828 | 211 | 192 | 9.0% | 3 | 11 | 4 | 1 | 206 | 5 | 2.4% |  |
| CG7033 | 201 | 139 | 30.8% |  | 1 | 12 | 49* | 205 | 8 | 3.9% | *low density |
|  | |  |  | |  |  |  | |  |  | |
| control | 233 | 215 | 7.7% |  | 4 | 14 |  | 213 | 7 | 3.3% |  |
| CG8258 | 200 | 102 | 49.0% |  | 2 | 1 | 95* | 216 | 2 | 0.9% | *low density |
| CG8231 | 207 | 103 | 50.2% | 1 |  | 3 | 100* | 217 | 2 | 0.9% | *low density |
|  | |  |  | |  |  |  | |  |  | |
| control | 205 | 197 | 3.9% | 1 | 3 | 4 |  | 202 | 5 | 2.5% |  |
| CG5525 | 210 | 142 | 32.4% |  | 1 | 2 | 65 * | 207 | 4 | 1.9% | *low density |
| CG13914 | 206 | 194 | 5.8% |  | 5 | 6 | 1 | 201 | 6 | 3.0% |  |
|  | |  |  | |  |  |  | 216 |  |  | |
| control | 204 | 193 | 5.4% | 2 | 7 | 2 |  |  | 8 | 3.7% |  |
| CG11881 | 214 | 197 | 7.9% | 1 | 12 | 4 |  | 212 | 10 | 4.7% |  |
| CG2213 | 203 | 180 | 11.3% | 2 | 16 | 4 | 1 | 230 | 8 | 3.5% |  |
| CG11700 | 201 | 151 | 24.9% | 8 | 2 | 2 | 38* | 221 | 6 | 2.7% | * straight MT |
|  | |  |  | |  |  |  |  |  |  | |
| ***hits*** | |  |  | |  |  |  |  |  |  | |
| control | 204 | 193 | 5.4% | 2 | 7 | 2 |  | 216 | 8 | 3.7% |  |
| CG11963 | 201 | 194 | 3.5% |  | 5 | 2 |  | 209 | 12 | 5.7% |  |
|  | |  |  | |  |  |  | |  |  | |
| control | 212 | 193 | 9.0% |  | 16 | 3 |  | 223 | 5 | 2.2% |  |
| CG8036 | 205 | 175 | 14.6% |  | 28 | 2 |  | 207 | 7 | 3.4% |  |
| CG12018 | 204 | 192 | 5.9% |  | 11 | 1 |  | 211 | 3 | 1.4% |  |
| CG8142 | 201 | 175 | 12.9% | 2 | 21 | 3 |  | 203 | 6 | 3.0% |  |
| CG11876 | 209 | 187 | 10.5% | 1 | 14 | 7 |  | 209 | 6 | 2.9% |  |
|  | |  |  | |  |  |  | |  |  | |
| control | 201 | 183 | 9.0% |  | 7 | 11 |  | 246 | 7 | 2.8% |  |
| CG10932 | 211 | 192 | 9.0% |  | 7 | 12 |  | 228 | 10 | 4.4% |  |
